# A novel Torque teno virus qPCR assay reveals longitudinal viral load variability among non-immunosuppressed subjects

**DOI:** 10.1038/s44321-026-00540-3

**Published:** 2026-10-08

**Authors:** Lara Valder, Thomas G Grigore, Christian Schaaf, Quirin Bachmann, Lutz Renders, Samuel D Jeske, Marc Lütgehetmann, Sibylle Bierbaum, Hartmut Hengel, Christopher Dächert, Ulrike Protzer, Jochen M Wettengel

**Affiliations:** 1 Technical University of Munich, School of Medicine and Health, Institute of Virology, Munich, Germany; 2 Technical University of Munich, School of Medicine and Health, Department of Nephrology, TUM University Hospital, Munich, Germany; 3 German Center for Infection Research (DZIF), Munich Partner Site, Munich, Germany; 4 Center for Diagnostics, Institute of Medical Microbiology, Virology and Hygiene, University Medical Center Hamburg-Eppendorf, Hamburg, Germany; 5 Institute of Virology, University Medical Center, Freiburg, and Faculty of Medicine, University of Freiburg, Freiburg im Breisgau, Germany; 6 Ludwig Maximilian University, Max von Pettenkofer Institute and Gene Center, Virology, National Reference Center for Retroviruses, Munich, Germany; 7 Helmholtz Munich, Institute of Virology, Munich, Germany

**Keywords:** Immunology, Methods & Resources, Stem Cells & Regenerative Medicine

## Abstract

Torque teno virus (TTV) DNA is an emerging biomarker of the individual immune status with considerable potential for guiding immunosuppressive therapy. However, widespread clinical implementation has been hampered by the lack of standardized, high-throughput diagnostic assays and limited understanding of the physiological variability of TTV viral loads over time. Here, we developed and analytically validated a novel fully automated high-throughput quantitative PCR assay for standardized TTV-DNA measurement in routine diagnostics and large-scale clinical studies. The assay demonstrated high analytical sensitivity and specificity, with a limit of detection of 32.3 copies/ml. Using this platform, we performed longitudinal profiling of plasma and serum samples collected over up to 51 months from non-immunosuppressed individuals. We observed substantial inter- and intra-individual fluctuations in TTV-DNA levels in a subset of healthy subjects, revealing intrinsic biological variability independent of immunosuppressive therapy. These findings establish a robust platform for standardized TTV monitoring while demonstrating that longitudinal viral load dynamics must be interpreted in the context of natural biological variation. Our study provides an important framework for the clinical implementation of TTV-guided immune monitoring and the need for development of evidence-based thresholds for individualized immunosuppressive therapies.

## Synopsis

A novel qPCR assay enables sensitive and specific TTV-DNA quantification. Longitudinal analysis of patient samples revealed pronounced viral load variability among non-immunosuppressed individuals, revealing intrinsic fluctuation as an important factor to consider in TTV-based immune monitoring.

- Standardized TTV-DNA quantification in routine diagnostics and large-scale studies was enabled by a novel fully automated high-throughput qPCR assay.
- High analytical sensitivity and specificity were achieved, with a limit of detection of 32.3 TTV-DNA copies/ml.
- Pronounced intra-individual TTV-DNA fluctuations independently of iatrogenic immunosuppression were observed in a subset of non-immunosuppressed individuals during longitudinal follow-up over periods of up to 51 months.
- Individual TTV-DNA variability was identified as an important factor to consider when interpreting longitudinal viral load changes for immune monitoring.

## Abstract (web summary)

A novel qPCR assay enables sensitive and specific TTV-DNA quantification. Longitudinal analysis of patient samples revealed pronounced viral load variability among non-immunosuppressed individuals, revealing intrinsic fluctuation as an important factor to consider in TTV-based immune monitoring.

## Graphical abstract

**Figure:**
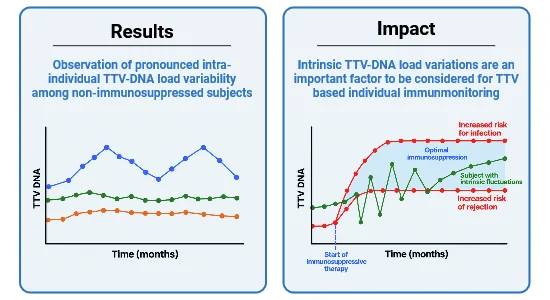

The paper explained **Problem**

Torque teno virus (TTV) DNA is considered a novel surrogate biomarker of the individual's immune status, particularly for monitoring patients receiving immunosuppressive therapy. However, its clinical implementation is limited by the lack of standardized, fully automated high-throughput assays and by insufficient knowledge of the intrinsic longitudinal variability of TTV-DNA levels. This is particularly relevant because changes in TTV-DNA load attributed to immunosuppressive therapy may overlap with physiological intra-individual fluctuations. Reliable clinical interpretation, therefore, requires both sensitive and standardized TTV-DNA quantification and a better understanding of TTV-DNA dynamics in individuals without iatrogenic immunosuppression.

**Results**

The authors developed and analytically validated a novel, fully automated high-throughput TTV-DNA-qPCR assay for standardized application in routine diagnostics and large-scale clinical studies. The assay demonstrated robust analytical performance, including high precision and linearity, no relevant matrix effects, and a limit of detection of 32.3 copies/ml. Compared with an adapted established TTV assay, the newly developed assay provided higher fluorescence signals and improved detection of low-positive samples on the cobas x800 platform. Longitudinal analysis of two independent cohorts of non-immunosuppressed individuals revealed pronounced inter- and intra-individual TTV-DNA variability in a subset of subjects, including substantial short-term fluctuations and long-term trends. Comparable viral load trajectories obtained with three different TTV assays supported that these fluctuations were not assay-specific artifacts.

**Impact**

This study establishes a sensitive and robust fully automated high-throughput platform for standardized TTV-DNA quantification and identifies intrinsic longitudinal TTV-DNA variability as an important factor for clinical immune monitoring. The observation that substantial short- and long-term fluctuations can occur independently of iatrogenic immunosuppression indicates that changes in TTV-DNA load cannot necessarily be attributed to therapeutic immune modulation alone. Consequently, reliable application of TTV as an individualized biomarker will require consideration of patient-specific baseline variability and clinical context rather than relying exclusively on universal absolute thresholds. Larger prospective studies will be required to distinguish physiological variation from therapy-related changes and to establish clinically actionable thresholds for TTV-guided immunosuppressive management.

## Introduction

Torque teno virus (TTV) is a single-stranded DNA virus belonging to the genus of *Alphatorqueviruses* in the family of *Anelloviridae* (Biagini, 2009; Cancela et al, 2023; Hino and Miyata, 2007; Sabbaghian et al, 2024; Spezia et al, 2023). It has been detected in a wide range of mammalian species and is particularly prevalent in humans, in whom infection is typically acquired early in life (Väisänen et al, 2022). During this early phase of infection, individuals acquire multiple variants of anelloviruses, including TTV, which together constitute the individual “anellome” that differs between persons and stabilizes in adulthood (Kaczorowska et al, 2022; Timmerman et al, 2024).

Following primary infection, TTV establishes long-term persistence with ongoing viral replication. Accordingly, TTV-DNA is detectable in ~70–100% of the global population (Jarkasi et al, 2019; Saback et al, 1999; Väisänen et al, 2022). Despite this high prevalence and persistent replication, TTV infection is generally assumed to be asymptomatic and has not been causally linked to any specific disease entity to date (Vietzen et al, 2024). TTV-DNA levels have been reported to show interindividual variability, and persistent viremia has been observed in healthy individuals without apparent clinical consequences (Vietzen et al, 2024). TTV viremia has been shown to remain relatively stable within individuals over time, with only modest fluctuations reflecting a dynamic balance between viral replication and immune control, the longitudinal data remain scarce (Focosi et al, 2020; Kaczorowska et al, 2022; Kyathanahalli et al, 2021; Saláková et al, 2004). In contrast, consistently higher TTV-DNA blood levels have been observed in individuals with impaired immune function, including people living with untreated HIV infection, patients receiving chemotherapy for malignant disease, or solid organ transplant (SOT) recipients receiving immunosuppressive therapy (Campo et al, 2000; Gallian et al, 1999; Schmidt et al, 2021; Shibayama et al, 2001; Spandole et al, 2015; Takemoto et al, 2015). TTV viral load analyses in immunocompromised cohorts after SOT have demonstrated that TTV-DNA levels correlate with the patient-related immune status, encompassing iatrogenic immunosuppression. In these settings, increases in TTV-DNA levels have been reported following initiation or increased dosing of immunosuppressive therapy, whereas decreases have been observed after dose reduction or discontinuation (Haupenthal and Bond, 2023; Querido et al, 2023). Together, these observations have led to the assessment of blood TTV-DNA levels as a dynamic readout of immune modulation, with potential utility as a surrogate marker of the individual’s immune status, particularly in immunocompromised populations (Fernández-Ruiz et al, 2019; Roberto et al, 2023; van Rijn et al, 2023). However, due to the considerable heterogeneity in study designs and methodological approaches, there are currently no standardized reference values for TTV-DNA loads during immunosuppression.

The TTV genome comprises ~3.8–3.9 kilobases and contains multiple open reading frames (ORFs) as well as a 1.0–1.2 kb non-coding untranslated region (UTR) that is essential for viral replication and regulation of gene expression (Halfon and Cacoub, 1999; Hino and Miyata, 2007; Liou et al, 2024; Okamoto et al, 1999; Roberto et al, 2023). TTV exhibits pronounced genetic diversity across coding and non-coding regions, complicating genotype classification and comparative analyses (Kraberger et al, 2021; Liou et al, 2024). Notably, a ~150 bp short segment within the UTR represents the most conserved genomic region across TTV genotypes and related human torque viruses, making it a preferred target for quantitative polymerase chain reaction (qPCR)-based detection and quantification (Arze et al, 2021; Hallett et al, 2000). Blood-derived specimens, including serum or plasma, are most frequently used for TTV-DNA detection or analysis because they provide reproducible quantification and are less affected by pre-analytical variability than alternative specimen types (Batista et al, 2022; Chan et al, 2001; Wolff et al, 2000).

At present, only a limited number of commercially available in vitro diagnostic (IVD)-certified assays for TTV-DNA detection are available, and these are typically restricted to specific analytical platforms or predefined analytical processes. Consequently, many laboratories rely on laboratory-developed tests (LDT) or published protocols (Itoh et al, 1999; Kato et al, 2000). Among these, the TTV-DNA-specific qPCR assay described initially by Maggi et al, targeting the conserved segment in the UTR region, is one of the most widely used approaches and has demonstrated broad genotype coverage and robust analytical performance (Maggi et al, 2001). While an adaptation of this assay has been implemented on a fully automated, random-access platform, previously described workflows have not been optimized for sustained high-throughput testing (Spezia et al, 2024). Platforms such as the cobas x800 system provide shorter setup times, reduced hands-on time, and faster times to first and last results than random-access platforms. Performance comparisons demonstrate high concordance of diagnostic results between the cobas x800 system and other random-access systems, indicating comparable clinical accuracy while achieving higher sample throughput and a more streamlined workflow (Aretzweiler et al, 2019; Craney et al, 2020; Frontzek et al, 2019).

In this study, we adapted the previously published and commonly used TTV-DNA-qPCR assay (Maggi et al, 2001) for implementation on the cobas x800 systems and validated it within a fully automated high-throughput workflow. However, this assay showed low relative fluorescence intensities (RFI), which compromised reliable signal detection and automated result interpretation. Therefore, an alternative TTV-DNA-qPCR assay was developed, optimized, and validated. In direct comparative analyses, the newly developed qPCR assay demonstrated increased analytical sensitivity while maintaining high specificity. Using this assay, we assessed factors affecting the biomarker potential of TTV, including plasma stability and age-related variation. Remarkably, we observed pronounced longitudinal fluctuations in TTV-DNA levels in a subset of patients without iatrogenic immunosuppression, suggesting that individual factors beyond immunosuppressive medication contribute to viral load variability. In summary, these findings demonstrate the assay’s suitability as a fully automated, high-throughput platform for clinical applications, but also indicate the need for longitudinal intra-individual monitoring to interpret the clinical significance of TTV-DNA levels.

## Results

### Adaptation of the originally published TTV-DNA-qPCR assay for implementation on the cobas x800 systems

To establish a fully automated, high-throughput TTV-DNA-qPCR assay for the cobas x800 systems, the most widely used TTV-DNA-specific qPCR assay, described initially by Maggi et al, was selected (Maggi et al, 2001). As this assay had previously been described for use with an annealing/elongation temperature of 55 °C (Spezia et al, 2024), its performance was evaluated in the context of the standard cobas x800 temperature profile, which specifies an annealing/cycling temperature of 58 °C. To assess the impact of this temperature adjustment, qPCR reactions were performed at both annealing temperatures using four different TTV-positive patient samples. Comparative analysis of the calculated viral loads revealed no significant differences between the two temperature conditions (Appendix Fig. S1). In addition, high-resolution melting analysis demonstrated specific amplification without evidence of unspecific products or primer-dimer formation (Appendix Fig. S2).

#### Modification of primers

Following the confirmation that the assay was compatible with the standard cobas x800 temperature profile, primer and probe modifications were evaluated to optimize assay performance for the cobas x800 system. For this, unmodified primers (n) and primers containing 2′-*O*-methyl modification variants at the 3′ end, with a single 2′-*O*-methyl modification at the penultimate base (m) or double modifications at both the terminal and penultimate bases (2 m), were generated (Schoenbrunner et al, 2017; Unat, 2022).

**Figure EV1:**
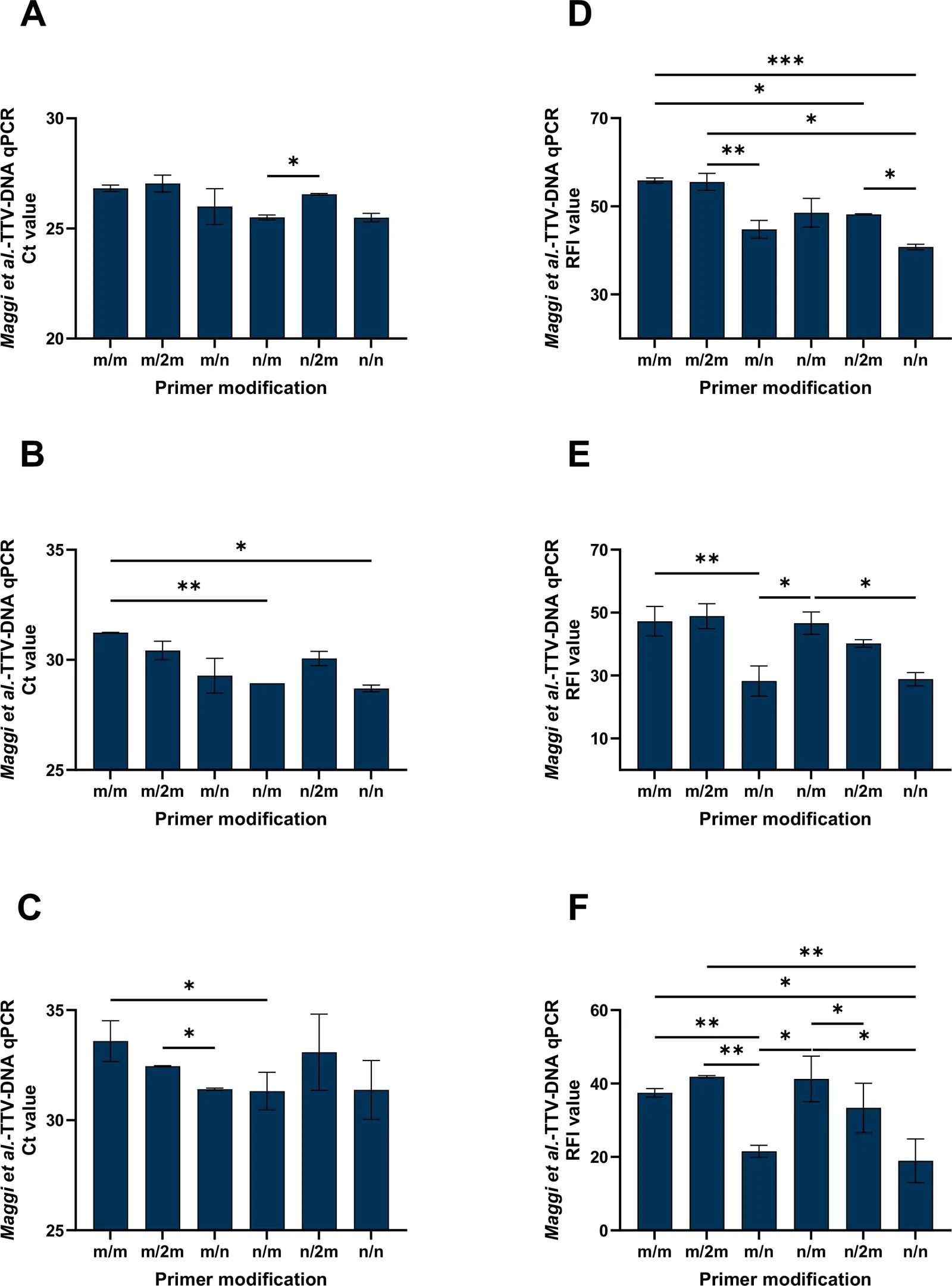
Comparison of modified primers for TTV detection in a serial dilution. (**A**–**C**) Ct values for the Maggi et al primer set and sample dilution. (**D**–**F**) RFI for each primer set and dilution. Three different sample dilutions were used to cover high and low viral titers: (**A**, **D**) Sample dilution 1:10; (**B**, **E**) Sample dilution 1:100; (**C**, **F**) Sample dilution 1:500. Measurement of a plasma sample serial dilution in triplicate. Data were shown as mean ± SD of triplicate measurements (*n* = 3). Statistical significance was assessed using a paired *t*-test. *P* < 0.05 is indicated by *, *P* < 0.01 by **, *P* < 0.001 by ***, and all comparisons not indicated were considered non-significant (*P* > 0.05). Exact *p* values are described in Appendix Table S4.

The primer variants were combined in all possible combinations and compared using a dilution series of a TTV-positive sample. PCR performance was evaluated based on Ct values and RFI (Fig. EV1).

In this analysis, only minor improvements in Ct values and RFI were observed among the modified primer sets, indicating that modifications to this particular primer set had only a limited impact on overall PCR performance and that most tested primer combinations would likely be suitable for the assay.

Nevertheless, among the evaluated combinations, the n/m primer set consistently demonstrated the best overall performance. Although the observed differences were relatively small, this primer set showed a slight trend for lower Ct values and higher RFIs, particularly in samples with low viral loads. Therefore, the n/m primer set was selected for all subsequent experiments.

#### Optimization of primer and probe concentration

Based on the previous results, the n/m primer-probe set was selected for further optimization, and reaction conditions were refined by adjusting primer and probe concentrations for use on the cobas x800 system. Primer concentration was optimized first by testing four different concentrations (0.1, 0.2, 0.3, and 0.4 µM) while maintaining a constant probe concentration of 0.05 µM (Appendix Fig. S3A,B), using a serial dilution of TTV-DNA-positive plasma samples. Among the tested concentrations, only minor differences in Ct values and RFI were observed. As the primer concentration of 0.4 µM was the assay’s standard starting concentration and yielded the highest RFI, it was used in subsequent experiments.

Next, probe concentration was optimized by testing four different concentrations (0.05, 0.1, 0.2, and 0.3 µM) at a constant primer concentration of 0.4 µM, corresponding to the previously optimized primer concentration (Appendix Fig. S3C,D), using a serial dilution of a TTV-DNA-positive plasma sample. Owing to pronounced differences in RFI and only minor differences in Ct values between probe concentrations, a probe concentration of 0.05 µM was selected for further TTV-DNA-qPCR assays.

### Validation on the cobas 5800

After adapting the assay to the cobas z 480/LC 480 II and optimizing it for use on the cobas x800 systems, the TTV-DNA LDT was validated on a cobas 5800 platform. Validation comprised assessment of intra-assay and inter-assay precision, linearity, and matrix dependency, and was performed in accordance with the EU In Vitro Diagnostic Regulation (IVDR, EU 2017/746) and the German Rili-BÄK guidelines.

Intra- and inter-assay precision were evaluated using multiple TTV-DNA-positive plasma samples with high (H1–H3), medium (M1–M3), and low (L1–L3) TTV-DNA loads, each analyzed in replicate measurements. Hereby, we observed that low-positive samples were classified as negative because their RFI values were below the standard minimum RFI (RFI_min_) threshold of 2.5. Therefore, the RFI_min_ threshold was set to 2.0, as low-positive samples exhibited RFIs between 2.0 and 2.2. This threshold allowed these samples to be correctly classified as positive; however, this adjustment also increased the risk of false-positive results.

**Figure 1:**
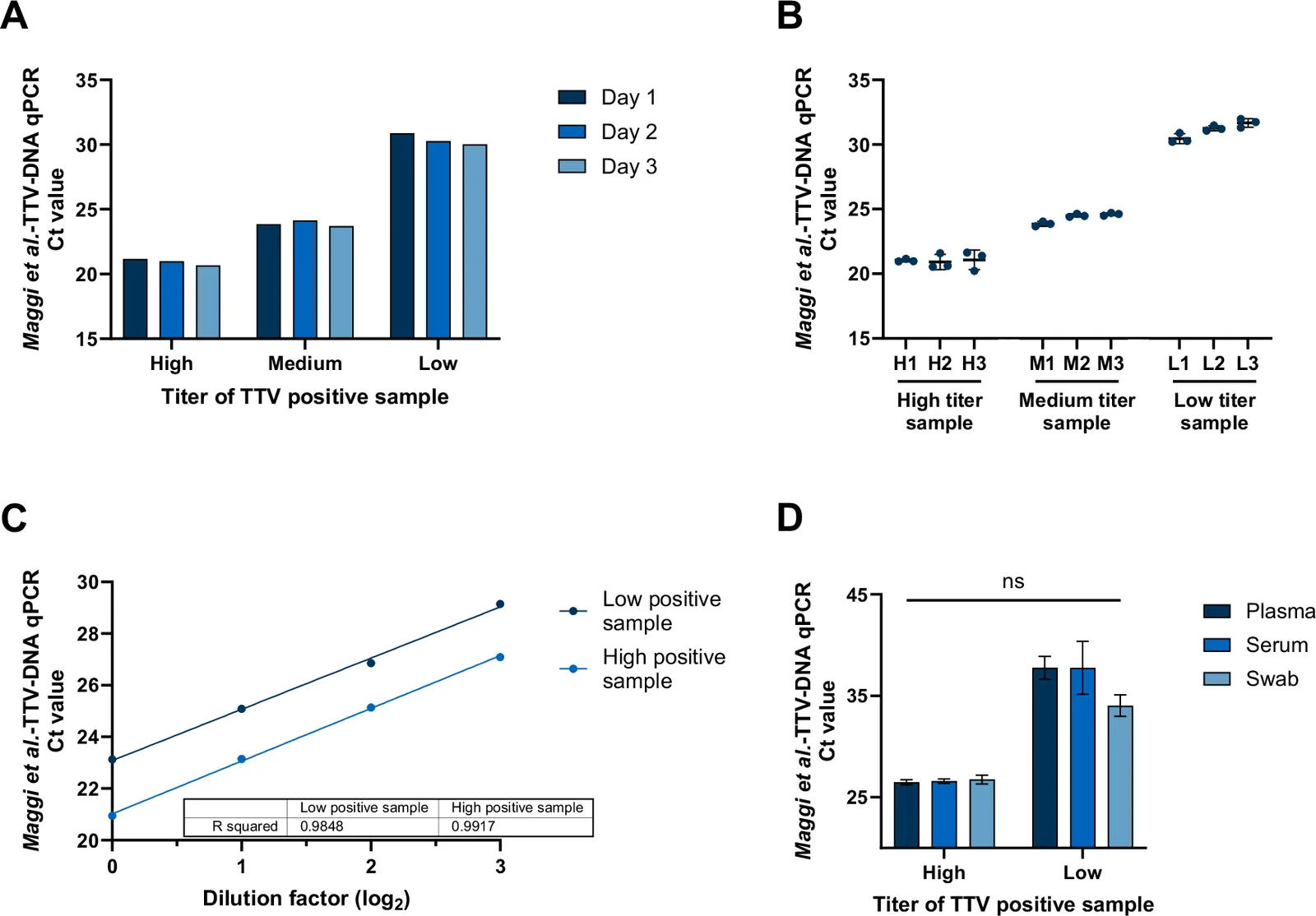
Analytical performance characteristics of the Maggi et al. TTV-DNA-qPCR assay (Maggi et al, 2001) on the cobas 5800. (**A**) Inter-assay precision of Ct values for high, medium, and low-positive plasma samples measured over 3 days. Each point represents the Ct value for a sample on a specific day, showing day-to-day variability. (**B**) Intra-assay precision of Ct values for high (H1–H3), medium (M1–M3), and low (L1–L3) positive samples within the same assay run. Measurement in triplicate (*n* = 3), with low variability indicating high intra-assay reliability. (**C**) Ct measurements in duplicates (*n* = 2) across serial dilutions for two different samples (high and low positive). Dilution series showed linear coefficients of determination (*R*²) of 0.9848 and 0.9917, respectively. (**D**) Three different negative matrices (plasma, serum, and swab) were spiked with TTV-DNA at high and low concentrations (*n* = 3). Statistical analysis was performed using a paired *t*-test. All comparisons were non-significant (ns, *P* > 0.05). Data information: In (**B**, **D**), data were presented as mean ± SD of triplicate measurements. Exact *p* values are described in Appendix Table S4. Source data are available online for this figure.

Inter-assay precision (Fig. 1A) showed low variability across the tested concentration ranges, with coefficients of variation of 1.16, 0.95, and 1.49% for samples with high, medium, and low TTV-DNA load, respectively. Samples with high, medium, and low TTV-DNA load also showed robust intra-assay precision, with a maximum coefficient of variation of 3.59% across technical triplicates (Fig. 1B).

Linearity of the assay was assessed using two TTV-DNA-positive plasma samples representing different viral load ranges. Both samples were analyzed using a four-step serial dilution (Fig. 1C). Across the tested dilution range, a strong linear relationship between input viral load and measured signal was observed for both samples. Regression analysis yielded coefficients of determination (*R*²) of 0.9848 and 0.9917, respectively, indicating excellent linearity of the assay over the evaluated concentration ranges.

To assess the assay’s matrix dependencies, three materials (plasma, serum, and swab) were selected. Negative samples were spiked with the same high and low TTV-DNA-positive plasma samples. Minor deviations were observed in the low-positive swab sample; however, these differences were not statistically significant. Overall, the assay showed no significant matrix-dependent effects in plasma, serum, or swab samples (Fig. 1D).

The assay specificity was confirmed by in silico analysis of the primer and probe sequences against the NCBI Virus/Gene database, demonstrating the absence of cross-reactivity with the human genome, human-pathogenic viruses, or bacteria.

The Maggi et al assay demonstrated robust analytical performance when used with a reduced RFI_min_ of 2.0, including precision across all concentration ranges, linearity over the tested dynamic range, and no significant, detectable matrix effects. Based on these validation results, the assay is suitable for use in routine diagnostic testing at an RFI_min_ of 2.0. However, due to the relatively low RFI, samples classified as negative on the cobas x800 platform may require manual review of amplification curves with the external cobas Omni Utility Channel Optimization Tool to reliably exclude false-negative results which limits the suitability of this assay for application on the cobas x800 platform in particular.

### Development of TTV-specific oligonucleotides optimized for the cobas x800 system

**Figure EV2:**
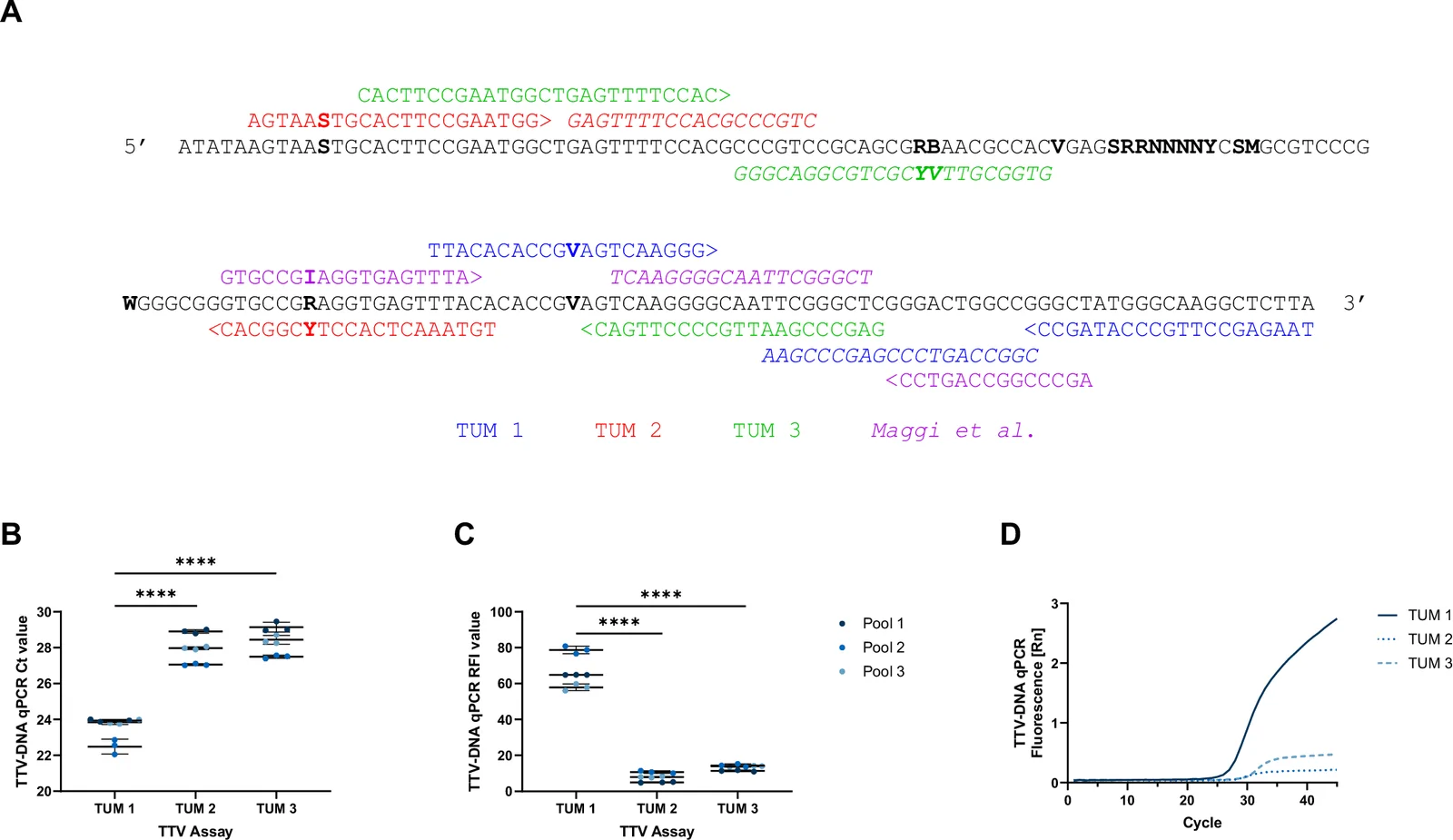
Selection of TTV-DNA-specific primers and probes. (**A**) Various primer pairs and probes are mapped across the TTV consensus sequence with 75% sequence similarity to facilitate detection and quantification in diagnostic assays. Degenerate bases are indicated in bold. Forward and reverse primers are marked with arrows in the direction of binding. Probes are indicated in italic. Blue: TUM 1, Red: TUM 2, Green: TUM 3; Purple: Maggi et al. (Maggi et al, 2001). (**B**–**D**) Comparison of different primer/probe sets for TTV-DNA detection. (**B**) Ct values and (**C**) RFI for each primer/probe set (*n* = 9), showing variation in sensitivity and signal strength. Sets with lower Ct values and higher RFI demonstrate superior sensitivity for TTV-DNA detection. (**D**) qPCR amplification curves demonstrate the fluorescence increase per cycle. (**B**, **C**) Data were shown as single measurements with mean ± SD of triplicate measurements. Statistical significance was assessed using a paired *t*-test. *P* < 0.0001 is indicated by ****, and all comparisons not indicated were considered non-significant (*P* > 0.05). Exact *p* values are described in Appendix Table S4.

As the PCR assay described by Maggi et al (Maggi et al, 2001) exhibited low RFI on the cobas 5800 platform and required manual review of results to maintain sufficient sensitivity for low-positive samples, we decided to develop a new assay for the cobas x800 system. First, all suitable TTV sequences were selected (Appendix Table S1), and a consensus sequence at 75% agreement was generated (Appendix Fig. S4). Consistent with the known high genetic heterogeneity of TTV, only a single highly conserved region was identified, located within the UTR comprising 172 bp (Appendix Fig. S5). Targeting this conserved region, oligonucleotides were designed applying strict design criteria (Appendix Table S2). The three best primer and probe sets were selected for further analysis. Interestingly, the alignment of these three oligonucleotides with the 75% consensus sequence showed overlapping regions with the primer and probe sequences previously described by Maggi et al (Maggi et al, 2001) (Fig. EV2A).

All alternative TTV-specific primer and probe sets selected were compared to identify the best-performing set for TTV-DNA amplification. Evaluation focused on comparison of Ct and RFI values (Fig. EV2). For this purpose, three different samples, each consisting of pooled plasma from ten individual donors, were analyzed on the cobas z 480/LC 480 II.

Comparative analysis showed that the TUM 2 and TUM 3 primer and probe sets yielded significantly higher Ct values than the TUM 1 set (Fig. EV2B). In line with this observation, RFI values obtained with TUM 2 and TUM 3 were significantly lower (Fig. EV2C,D).

To further investigate the PCR performance of the three primer/probe sets as well as to evaluate assay specificity and exclude primer-dimer formation, all primer pairs were assessed by high-resolution melting (HRM) analysis (Appendix Fig. S6). A pooled sample consisting of plasma from ten TTV-positive subjects was used for this HRM analysis. Specific TTV-DNA amplification was observed only for both TUM 1 and TUM 3 primer sets. For the TUM 3 primers, an additional non-specific amplicon with a higher melting temperature was detected. No melting peaks indicative of primer-dimer formation were observed for any of the tested primer sets.

Based on these results, only the TUM 1 primer and probe set demonstrated high sensitivity and signal intensity, meeting the performance requirements of this TTV-DNA-qPCR assay, and was therefore selected for subsequent experiments.

#### Modification of primers

**Figure EV3:**
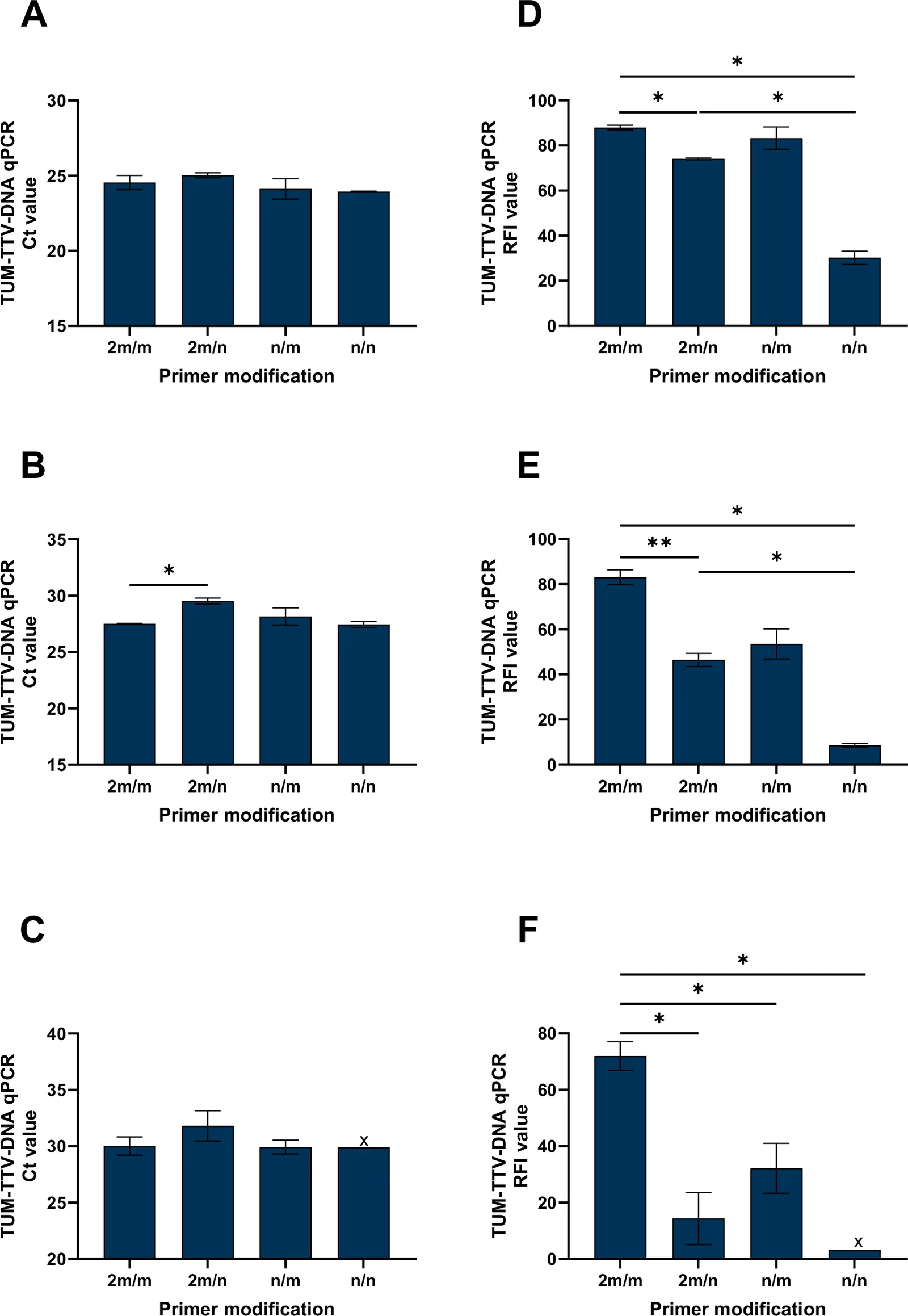
Comparison of modified primers for TTV detection in a serial dilution. (**A**–**C**) Ct values for the TUM 1 primer set and sample dilution. (**D**–**F**) RFI for each primer set and dilution. Three different sample dilutions were used to cover high and low viral titers: (**A**, **D**) Sample dilution 1:10; (**B**, **E**) Sample dilution 1:100; (**C**, **F**) Sample dilution 1:500. Measurement of a plasma sample serial dilution in triplicate. Data were shown as mean ± SD of triplicate measurements (*n* = 3). Statistical significance was assessed using a paired *t*-test. *P* < 0.05 is indicated by *, *P* < 0.01 by **, and all comparisons not indicated were considered non-significant (ns, *P* > 0.05). x indicates that only one replicate could be detected. Exact *p* values are described in Appendix Table S4.

The TUM 1 primer and probe set shows the best overall PCR performance. Therefore, the primers were modified with 2′-*O*-methyl groups at the 3′ end. All primer variants were subsequently tested by PCR using all possible forward and reverse primer combinations. Also, a pooled sample consisting of plasma from ten TTV-positive subjects was used for this analysis. PCR performance was evaluated based on Ct and RFI values (Appendix Fig. S7). Three primer modifications showed significantly higher RFI values without an associated increase in Ct values. They were therefore selected for further evaluation in a following experiment using a dilution series (Fig. EV3). In this analysis, no significant differences in Ct values were observed between the primer modifications, whereas significant differences in RFI were detected (Fig. EV3D–F).

In summary, the preceding experiments demonstrate that modification of the TUM 1 primers resulted in a substantial increase in relative fluorescence intensity while maintaining low Ct values, i.e., high sensitivity. Among the tested variants, the TUM 1 (2 m/m) primer configuration showed the most favorable overall PCR performance as indicated by Ct values and RFI, and was therefore selected for the alternative TTV-DNA-qPCR assay.

#### Optimization of primer and probe concentration

In a subsequent step, the primer and probe concentrations of the TUM 1 (2 m/m) primer and probe set were optimized under the same conditions as described previously. Primer concentration was evaluated, with 0.4 µM resulting in the most favorable PCR performance in terms of Ct values and RFI and therefore selected for all experiments (Appendix Fig. S8A,B). Subsequently, probe concentration was optimized under the same experimental conditions (Appendix Fig. S8C,D). A probe concentration of 0.05 µM yielded markedly higher RFI values with only minor differences in Ct values compared to the higher probe concentrations and was therefore selected for further evaluation.

#### Sequence ambiguities in the target sequence

Since the TUM 1 primer and probe set was designed based on a consensus sequence generated at 75% sequence identity across all TTV sequences included in the analysis, increasing the sequence similarity threshold to 85 or 95% resulted in a marked increase in sequence ambiguity, characterized by multiple degenerate bases within the consensus sequence (Appendix Fig. S5). A single degenerate base (V, representing A, C, or G) was already present in the TUM 1 forward primer sequence and was accounted for during primer design. When the sequence identity threshold was increased to 95%, nine additional degenerate bases were present within the target region of the TUM 1 primer and probe set that had not been considered in the original design (Appendix Fig. S9).

**Figure EV4:**
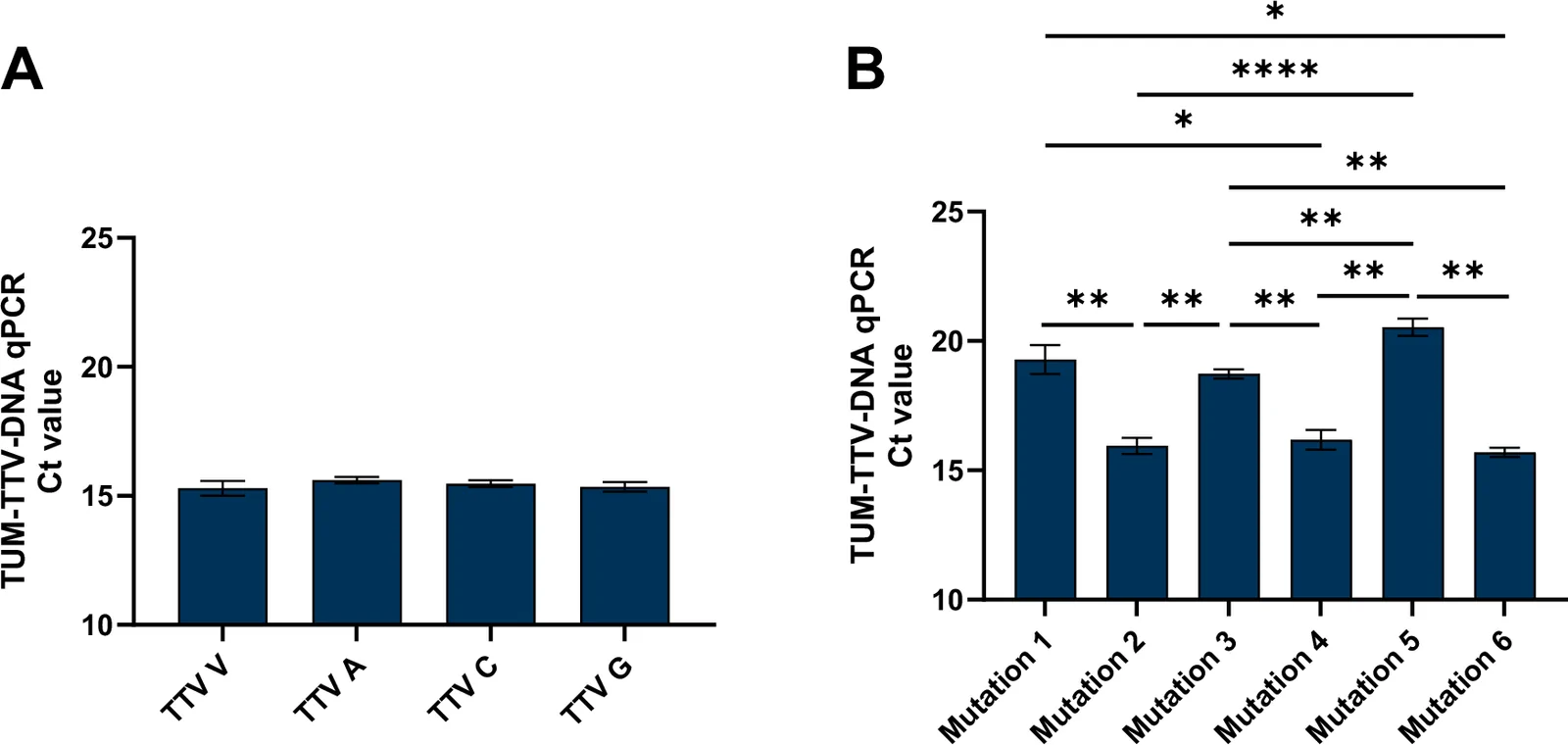
Comparison of TTV detection of different TTV constructs and mutations. (**A**) As a reference, TTV V represents the standard target sequence. TTV A, C, and G each represent the individual sequences contained within TTV V. (**B**) The following six mutations represent the most common mutations. Plasmids were measured in triplicate and the mean and standard deviation were calculated. Data were shown as mean ± SD of triplicate measurements (*n* = 3). Statistical significance was assessed using a paired *t*-test. *P* < 0.05 is indicated by *, *P* < 0.01 by **, *P* < 0.0001 by ****, and all comparisons not indicated were considered non-significant (*P* > 0.05). Exact *p* values are described in Appendix Table S4.

At these positions, 10–25% of the analyzed TTV sequences contained nucleotide variants differing from the bases targeted by the TUM 1 primers and probe. As such mismatches could potentially reduce primer and probe binding efficiency and thereby impair PCR sensitivity, the performance of the TUM 1 (2 m/m) primer and probe set was evaluated in the presence of defined sequence variability. For this purpose, nine plasmids containing the TTV target sequence with specific point mutations were generated (Appendix Fig. S10) and analyzed using the TUM 1 (2 m/m) qPCR assay (Fig. EV4). Mutations 1, 3, and 5 resulted in significantly increased Ct values, indicating reduced primer and probe binding efficiency and decreased detection sensitivity. Nevertheless, the assay detected all tested mutant TTV sequences; however, analytical sensitivity was approximately tenfold lower for some variants.

Taken together, these results demonstrate that the TUM 1 (2 m/m) primer and probe set, in combination with the optimized primer and probe concentrations, enables detection of more than 95% of the evaluated TTV sequence variants, although sensitivity is reduced at lower concentrations for a subset of variants. The assay is hereafter referred to as the TUM-TTV-DNA-qPCR assay.

### Validation of the TUM-TTV-DNA-qPCR assay on cobas 5800

After successful establishment of the TUM-TTV-DNA-qPCR assay on the cobas z 480/LC 480 II, the performance of the TTV LDT was validated on the fully automated cobas 5800. Validation included assessment of intra- and inter-assay precision, linearity, matrix dependency, and analytical specificity and sensitivity and was performed in accordance with the EU In Vitro Diagnostic Regulation (IVDR, EU 2017/746) and the German Rili-BÄK guidelines. The assay exhibited sufficient RFI to allow the RFI_min_ to be raised from the standard 2.5 to 2.7, thereby significantly reducing the likelihood of false-positive sample detection.

**Figure 2:**
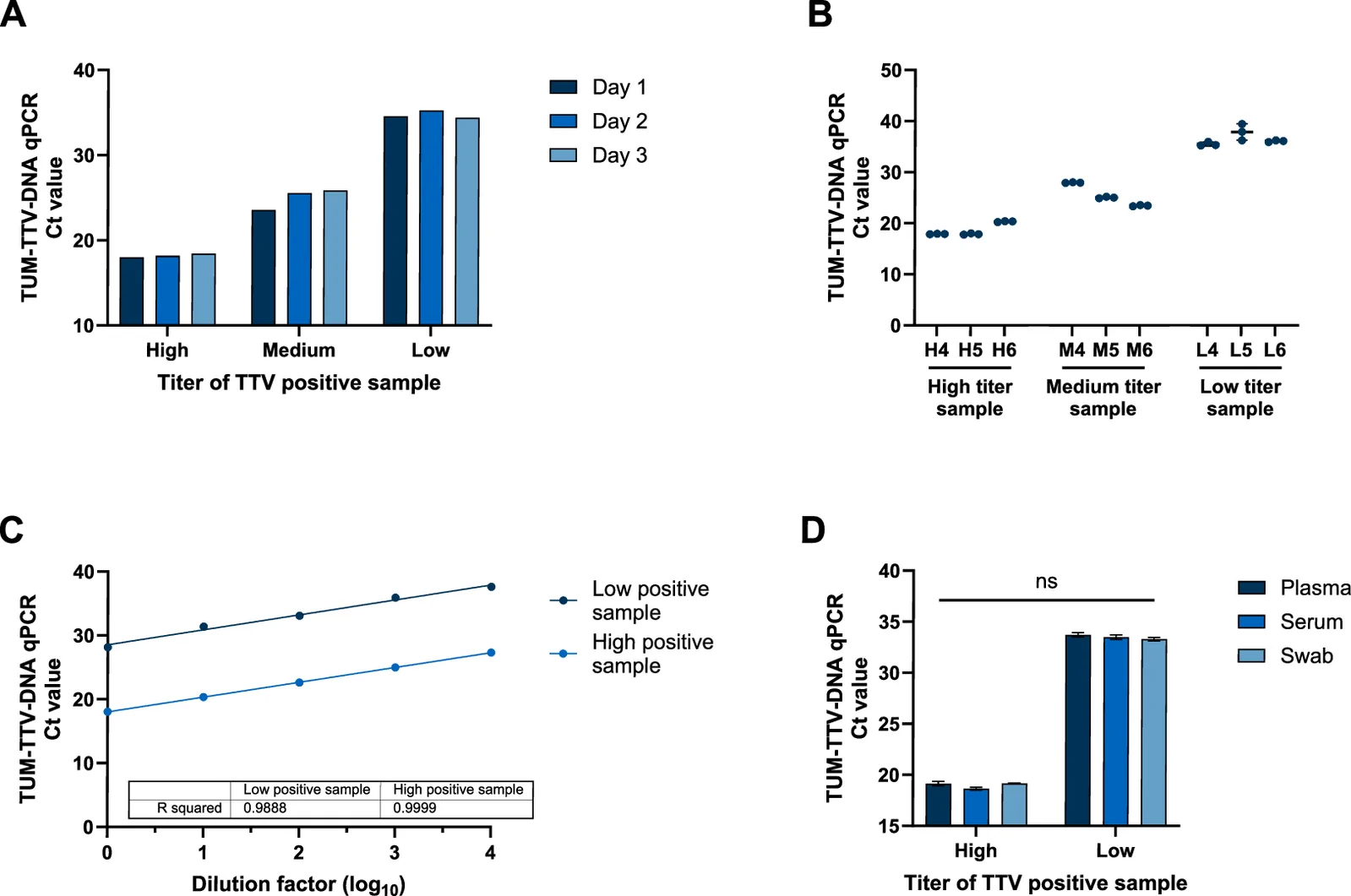
Analytical validation of the TUM-TTV-DNA-qPCR assay on the cobas 5800 system. (**A**) Inter-assay precision of Ct values for high, medium, and low TTV-DNA-positive plasma samples measured over three consecutive days, illustrating day-to-day variability. (**B**) Intra-assay precision of Ct values for high (H4–H6), medium (M4–M6), and low (L4–L6) TTV-DNA-positive plasma samples measured in triplicate (*n* = 3) within a single assay run, demonstrating low intra-assay variability. (**C**) Linearity of the qPCR assay was assessed using five-step serial dilutions of two TTV-DNA-positive plasma samples representing different viral load ranges. Ct values were measured in duplicate (*n* = 2) across the dilution series. Both samples showed linear coefficients of determination (*R*²) of 0.9888 (low positive) and 0.9999 (high positive). (**D**) Three different negative matrices (plasma, serum, swab) were spiked and measured at high and low TTV-DNA concentrations (*n* = 3). Statistical analysis was performed using a paired *t*-test. All comparisons were non-significant (ns, *P* > 0.05). Data information: In (**B**, **D**), data were presented as mean ± SD of triplicate measurements. Exact *p* values are described in Appendix Table S4. Source data are available online for this figure.

Inter-assay and intra-assay precision were evaluated using multiple new TTV-DNA-positive plasma samples covering a broad range of high (H4–H6), medium (M4–M6) and low (L4–L6) TTV-DNA load levels (Fig. 2A,B). Inter-assay precision remained robust across the tested concentration ranges, with coefficients of variation of 1.28, 4.98, and 1.16% for high, medium, and low TTV-DNA load samples. All concentration levels also demonstrated robust intra-assay precision, with a maximum coefficient of variation of 4.25%.

Linearity of the qPCR assay was assessed using two TTV-DNA-positive plasma samples representing different viral load ranges. Both samples were analyzed using a five-step serial dilution (Fig. 2C). Across the tested dilution range, a linear relationship between input viral load and measured signal was observed, with coefficients of determination (*R*²) of 0.9888 and 0.9999, respectively.

Matrix dependency of the assay was evaluated using three different sample materials: plasma, serum, and swab medium. Matrix-negative samples were spiked with the same high TTV-DNA-positive plasma sample and analyzed in parallel (Fig. 2D). No significant differences in assay performance were observed between the tested matrices.

**Figure EV5:**
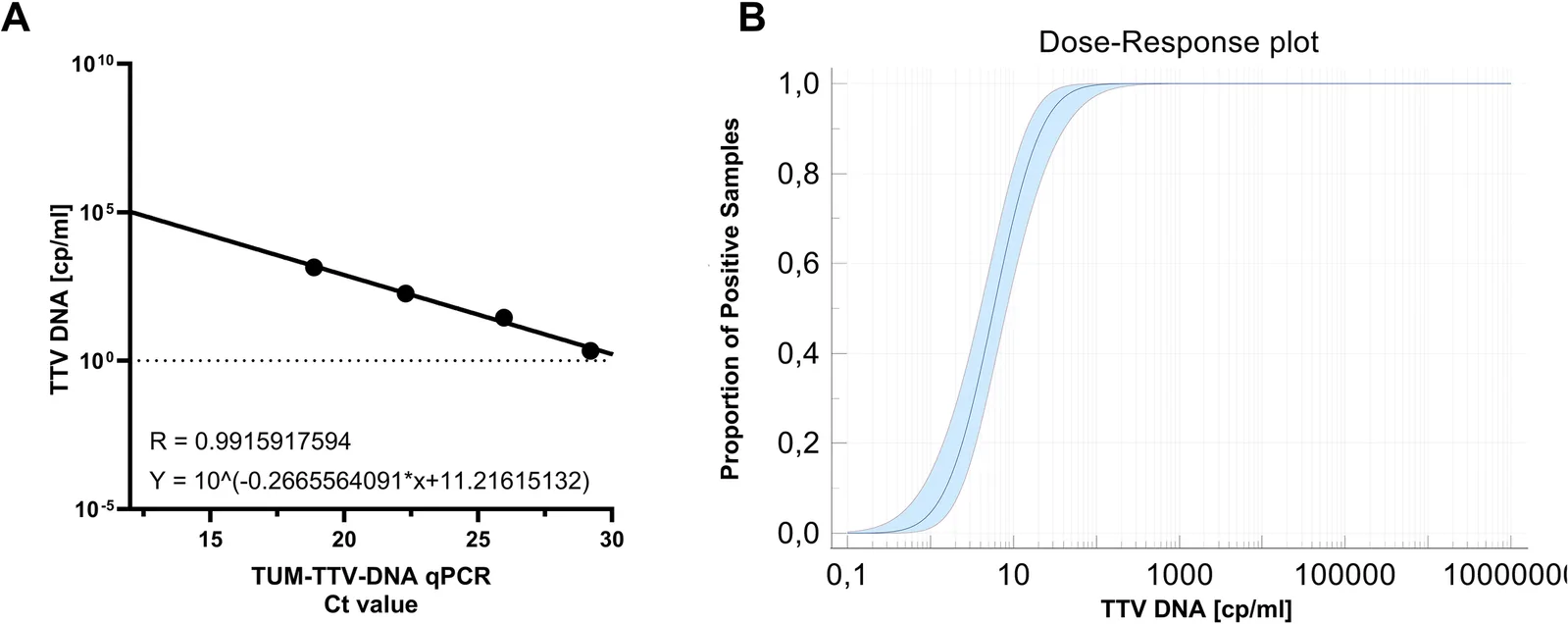
Analytical performance of the TUM 1 TTV PCR Assay on the cobas 5800. (**A**) Standard curve for assay quantification. Reference samples quantified by digital PCR (dPCR) and expressed as viral load (copies/mL) were plotted against the corresponding cycle threshold (Ct) values obtained on the cobas 5800. Nonlinear regression analysis was performed to derive the conversion equation used to calculate viral load (cp/ml) from measured Ct values. (**B**) Determination of the limit of detection (LOD). A serial dilution of a positive clinical sample was prepared, and each dilution level was measured 15 times (*n* = 15). The LOD was defined as the lowest concentration at which ≥95% of replicates were detected, based on probit analysis.

Analytical sensitivity of the assay was assessed using serial dilutions of TTV plasmid spiked plasma samples, with viral concentrations quantified by digital PCR. The same samples were subsequently analyzed on the cobas 5800 system and assigned to the corresponding viral load values. Based on these data, a standard curve was generated, and the regression equation was determined, which serves as the basis for converting Ct values into viral loads expressed as cp/ml (Fig. EV5A). To determine the 95% limit of detection (LOD_95_), a serial dilution series of a positive clinical plasma sample was prepared, and each dilution was tested 15 times using the assay on the cobas 5800 system (Fig. EV5B). The assay demonstrated a 95% LOD for TTV-DNA of 32.3 cp/ml (95% CI, 20.1–67.3), analyzed using Probit regression (dose-response analysis) in MedCalc.

To further evaluate the analytical performance of the TUM-TTV-DNA-qPCR assay, an inter-laboratory comparison was performed. Three independent laboratories analyzed a shared panel of ten highly positive and ten individual diluted low positive as well as four negative samples, using their own respective validated TTV-DNA PCR assay. In the comparison, assay sensitivity was evaluated by confirming the absence of false-negative results across laboratories in the 20 high and low-positive samples, while specificity was assessed by verifying the absence of false-positive detections in the four negative samples. The results were compared across the entire concentration range, and the inter-laboratory analysis demonstrated consistent detection, high specificity, and high sensitivity across the tested range, supporting the robustness and reliability of the TUM assay (Appendix Fig. S11).

The assay demonstrated robust analytical performance, including precision across all concentration ranges, linearity over the tested dynamic range, an adequate LOD comparable to other diagnostic standard tests, and no detectable matrix effects. An inter-laboratory comparison showed high concordance, indicating high sensitivity and specificity. In addition, assay specificity was supported by in silico analysis of the primer and probe sequences against the NCBI Virus/Gene database, confirming the absence of relevant cross-reactivity with the human genome and human-pathogenic viruses or bacteria.

### Comparison of two validated TTV-DNA-qPCR assays

**Figure 3:**
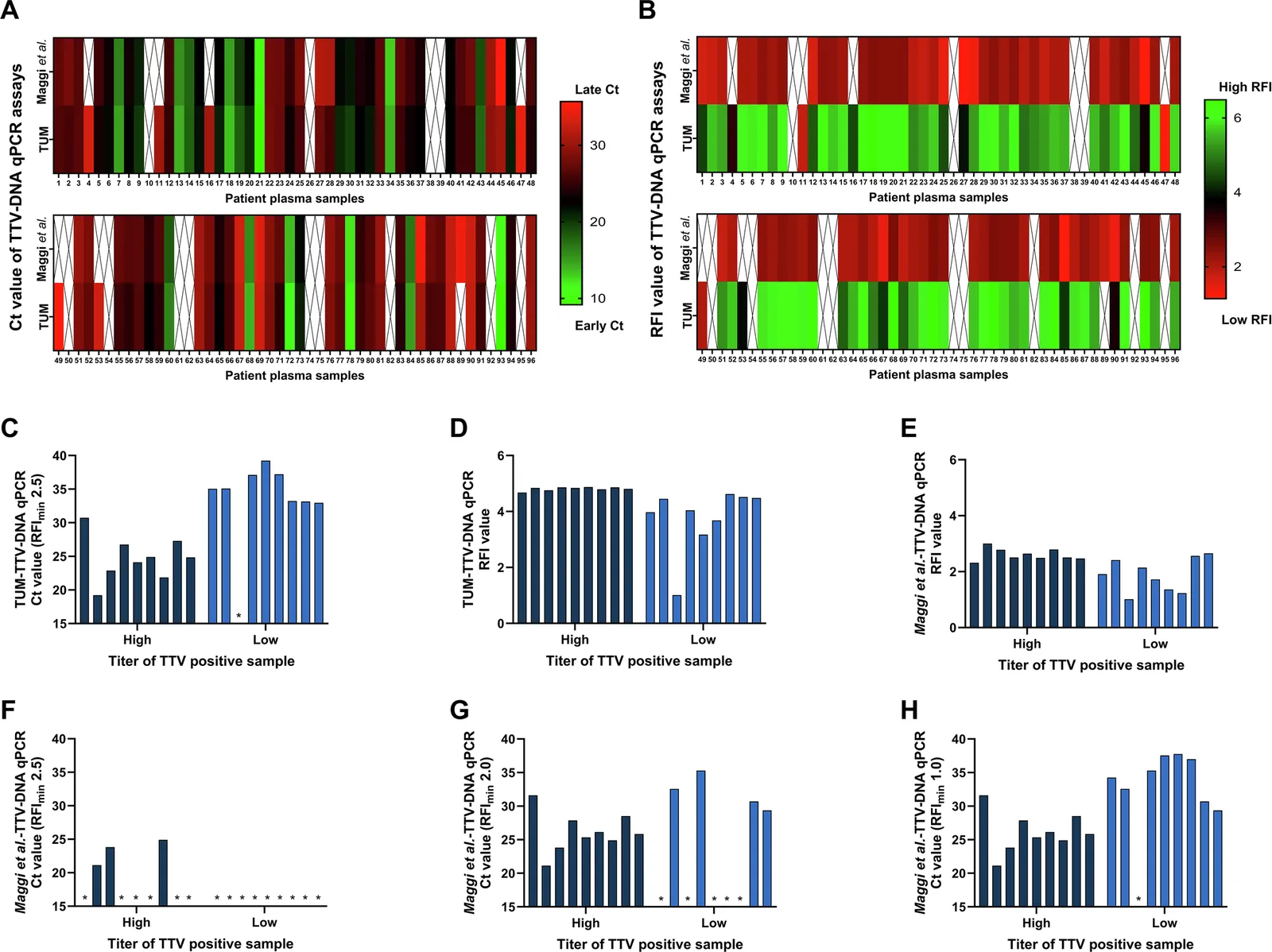
Comparison of two TTV-DNA-qPCR assays. (**A**,** B**) Comparison of 96 clinical samples. A total of 96 samples were analyzed using the two different TTV-DNA-qPCR assays. The heatmap visualizes the distributions of Ct and RFI across samples, with green indicating lower Ct values (higher viral load) and higher RFI, and red indicating higher Ct values (lower viral load) and lower RFI. Differences in color patterns between the two primer sets suggest variability in sensitivity or detection efficiency, potentially reflecting differences in primer-target binding affinity or amplification efficiency, as well as probe efficiency. (**A**) Ct values and (**B**) RFI obtained with each assay (*n* = 3) were compared to assess analytical concordance. (**C**–**H**) Impact of RFI threshold settings on detection of high and low TTV-positive samples on the cobas 5800. Nine high-positive and nine low-positive samples were analyzed using the two TTV PCR assays on the cobas 5800. The default minimum relative fluorescence intensity (RFI_min_) threshold was set to 2.5. (**C**) Ct values of all samples analyzed with the TUM assay at an RFI_min_ of 2.5 and (**D**) RFI for all samples analyzed with the TUM assay. (**E**) RFI for all samples analyzed with the Maggi et al. assay. (**F**) Ct values of all samples analyzed with the Maggi et al. assay at an RFI_min_ of 2.5. Due to lower RFI signals observed with the Maggi et al. assay, several samples were classified as negative, resulting in false-negative results. Reducing the RFI_min_ threshold to 2.0 (**G**) enabled reliable detection of high-positive samples, whereas detection of low-positive samples required a further reduction of RFI_min_ to 1.0 (**H**), representing the lowest permissible threshold setting. * indicates non-detectable TTV-DNA load. Source data are available online for this figure.

As a last step, both adapted assays, the Maggi et al qPCR assay and the TUM-TTV-DNA-qPCR, were directly compared for performance on the cobas 5800 system. For this purpose, 96 plasma samples from individual donors were analyzed in parallel using both primer and probe sets (Fig. 3A,B). Eleven samples that tested positive with only one of the assays or showed Ct value differences greater than four cycles between the two assays were re-analyzed in triplicate in a second PCR experiment to enable a more robust comparison of assay performance (Appendix Fig. S12).

Consistent with the findings described above, the Bland–Altman analysis (Appendix Fig. S13) demonstrated a mean bias of 3.370 RFI values, indicating that the TUM-TTV-DNA-qPCR assay produced, on average, higher RFI values than the Maggi et al assay. The 95% limits of agreement ranged from 2.567 to 4.174 RFI values. In contrast, differences in Ct values between the two assays were comparatively small. Here, Bland–Altman analysis revealed a mean bias of −1.319 Ct, indicating that the Ct values of the TUM-TTV-DNA-qPCR assay were, on average, 1.319 Ct lower than those obtained with the Maggi et al assay. The corresponding 95% limits of agreement ranged from −3.470 to 0.831 Ct.

To further investigate the impact of signal intensity on assay performance, the RFI_min_ was set to the recommended minimal value of 2.5, and a comparative analysis was performed using nine low-positive and nine high-positive plasma samples analyzed on the cobas 5800 system with both assays (Fig. 3C–H). In this comparison, the lower RFI generated by the Maggi et al primer and probe set resulted in a substantial number of false-negative results, especially in the low-positive sample range. This would lead to a significantly higher LOD_95_ with an RFI_min_ of 2.5. Lowering the RFI_min_ threshold for the Maggi et al. assay to the required value of 1.5 can, in some cases, result in false-positive samples (Appendix Fig. S14). Therefore, manual inspection of amplification curves would be required to exclude such false positives.

In summary, comparative analyses showed that the TUM-TTV-DNA-qPCR assay provided superior PCR performance on the cobas x800 system compared to the Maggi et al assay, primarily due to higher relative fluorescence intensities.

### Stability of TTV in blood samples

**Figure EV6:**
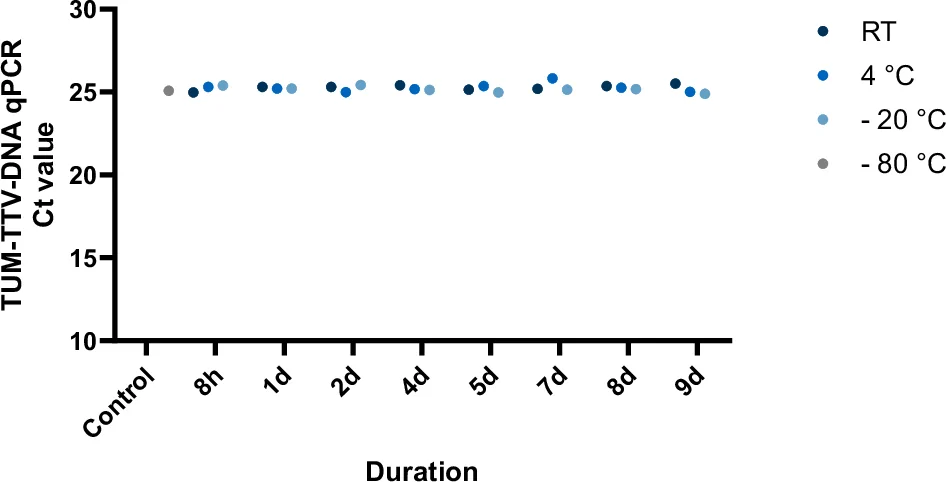
TTV stability at different temperatures. The samples were stored for a specific number of days at various temperatures (RT, 4 °C, −20 °C) and subsequently kept at −80 °C. A control sample, which was exclusively stored at −80 °C, was also used for comparison. Data were presented as the mean of triplicate measurements (*n* = 3). Two-way ANOVA revealed no significant effect of temperature (*F* (3, 21) = 1.897, *P* = 0.1611), and no significant effect of time (*F* (7, 21) = 0.3323, *P* = 0.9302).

To enable routine use of the TUM-TTV-DNA-qPCR assay, the stability of TTV-DNA in plasma for up to 9 days was assessed to ensure reliable and comparable quantification across different pre-analytical conditions, particularly for longitudinal monitoring and clinical decision-making. Plasma TTV-DNA remained stable in plasma for up to nine days at room temperature, 4 °C, and −20 °C, relative to a control sample stored at −80 °C for nine days (Fig. EV6), and two-way ANOVA revealed no significant effect of temperature or of time between all samples. These findings indicate that these pre-analytical storage conditions of plasma samples do not appear to compromise TTV-DNA detection, supporting the assay’s suitability for routine diagnostic use.

### Long-term course of TTV-DNA in subjects without iatrogenic immunosuppression

**Figure 4:**
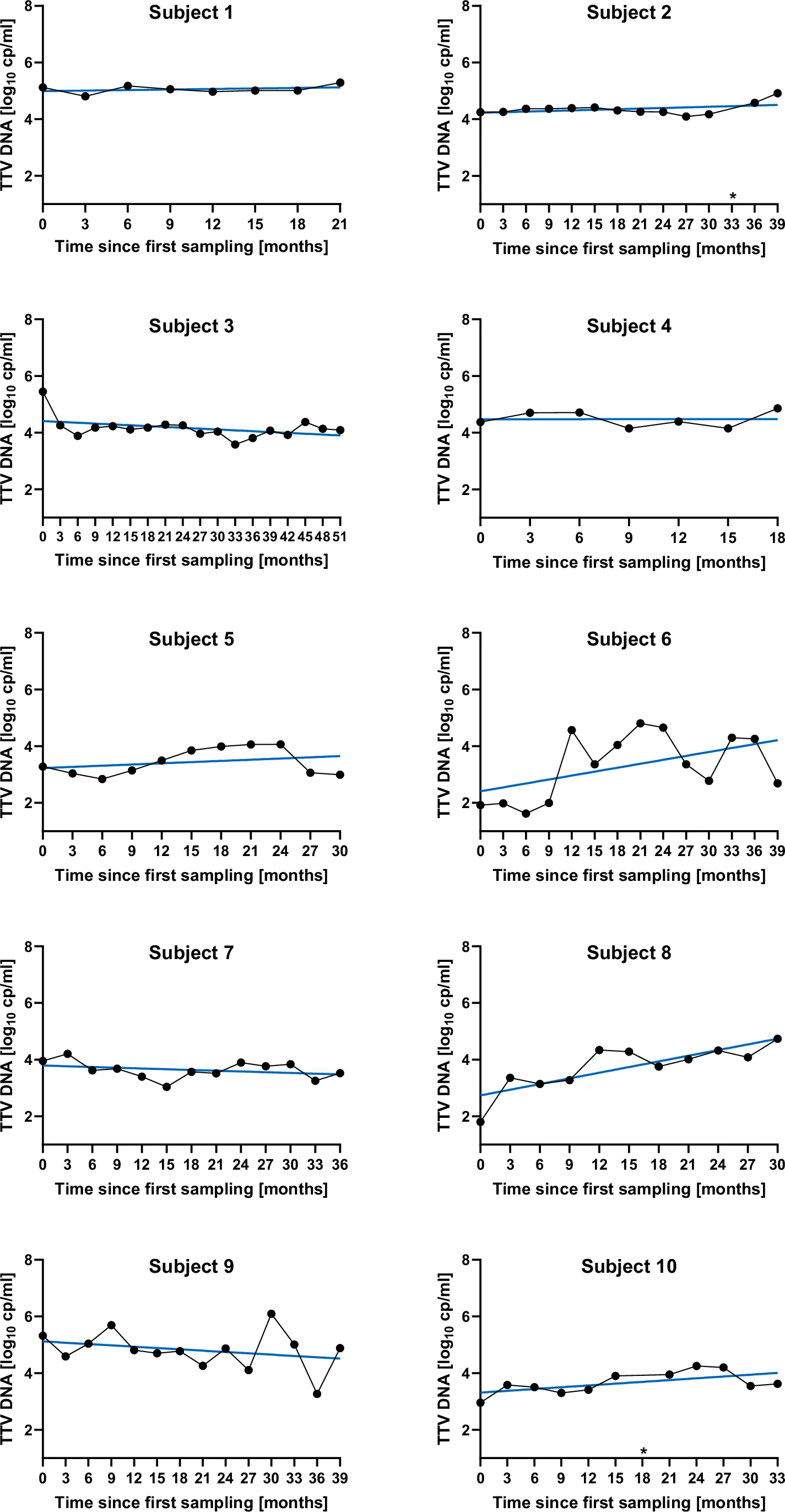
Longitudinal TTV-DNA levels in patients without iatrogenic immunosuppression. TTV-DNA concentrations were measured longitudinally in plasma samples from ten patients without any iatrogenic immunosuppressive medication. Individual patient time courses are shown (black) to illustrate intra-individual fluctuations of TTV-DNA over 18–51 months. Trend lines of TTV-DNA were generated using linear regression of the transformed data (blue). * indicates a missing data point. Source data are available online for this figure.

TTV has been proposed as an individual biomarker of immunosuppression, particularly in SOT recipients, who typically receive intensive immunosuppressive therapy immediately after transplantation, followed by long-term maintenance immunosuppression. It has been described that short-term increases in TTV-DNA followed intensification of immunosuppression. To correctly interpret such changes, it is essential to investigate the occurrence and extent of natural individual TTV-DNA variability in subjects without iatrogenic immunosuppression. However, longitudinal data on TTV dynamics in healthy individuals, particularly over extended observation periods, remain scarce. Given the limited availability of such data, we analyzed TTV-DNA dynamics over time in 10 subjects without relevant comorbidities or iatrogenic immunosuppression, approximately every three months (Fig. 4; Table 1). This cohort consisted of male subjects aged 50 to 73 years who remained free of HBV and HCV infection throughout the observation period and had no evidence of HIV infection, thereby minimizing the potential influence of chronic viral hepatitis and, in particular, HIV-associated immune dysfunction on TTV-DNA dynamics.

**Table 1:** Mathematical characterization of longitudinal variability in TTV-DNA loads in subjects with no iatrogenic-immunosuppression.

Name and observation period	Mean TTV-DNA (Min.-Max.) [cp/ml]	Dynamic range Min. to Max. [cp/ml]	Fold change Max/Min	SD	Coefficient of variation	Rate of change [cp/ml per month]	Maximum intra-quarter dynamic range [cp/ml]	Maximum intra-quarter fold change	Longitudinal slope
Subject 1 21 months	1.19E + 05 (6.43E + 04–1.94E + 05)	1.30E + 05	3.0 (0.48 log_10_)	3.96E + 04	0.36	6.18E + 03	9.10E + 04	2.3 (0.37 log_10_)	6.33E-03
Subject 2 39 months	2.60E + 04 (1.24E + 04–8.22E + 04)	6.98E + 04	6.6 (0.8 log_10_)	1.80E + 04	0.88	1.79E + 03	4.45E + 04	2.2 (0.3 log_10_)	7.00E-03
Subject 3 51 months	3.84E + 04 (7.70E + 03–2.80E + 05)	2.72E + 05	36.4 (1.56 log_10_)	8.02E + 04	5.35	5.34E + 03	2.62E + 05	15.6 (1.19 log_10_)	−9.96E-03
Subject 4 18 months	3.58E + 04 (1.42E + 04 - 7.20E + 04)	5.78E + 04	5.1 (0.71 log_10_)	2.22E + 04	0.90	3.21E + 03	5.78E + 04	5.1 (0.71 log_10_)	2.82E-04
Subject 5 30 months	4.57E + 03 (6.90E + 02–1.16E + 04)	1.09E + 04	16.8 (1.2 log_10_)	4.49E + 03	2.36	3.64E + 02	1.04E + 04	10.1 (1.0 log_10_)	1.41E-02
Subject 6 39 months	1.44E + 04 (4.20E + 01 - 6.40E + 04)	6.40E + 04	1523.8 (3.18 log_10_)	2.09E + 04	8.91	1.64E + 03	5.30E + 04	373.7 (2.57 log_10_)	4.63E-02
Subject 7 36 months	5.41E + 03 (1.10E + 03–1.60E + 04)	1.49E + 04	14.5 (1.16 log_10_)	3.95E + 03	0.94	4.14E + 02	1.18E + 04	3.9 (0.6 log_10_)	−8.71E-03
Subject 8 30 months	1.37E + 04 (6.30E + 01 - 5.45E + 04)	5.45E + 04	865.4 (2.94 log_10_)	1.58E + 04	1.53	1.82E + 03	4.24E + 04	36.5 (1.56 log_10_)	6.66E-02
Subject 9 39 months	1.83E + 05 (1.85E + 03–1.25E + 06)	1.25E + 06	673.2 (2.83 log_10_)	3.30E + 05	4.75	3.19E + 04	1.23E + 06	97.4 (1.99 log_10_)	−1.56E-02
Subject 10 33 months	6.45E + 03 (9.00E + 02–1.79E + 04)	1.70E + 04	19.9 (1.3 log_10_)	5.71E + 03	1.50	5.16E + 02	1.24E + 04	4.5 (0.7 log_10_)	2.34E-02

Interindividual mean TTV levels varied considerably as expected, ranging from 4.57 × 10^3^ cp/ml (Subject 5) to 1.8 × 10^5^ cp/ml (Subject 9). Notably, intra-individual variation showed a heterogeneous pattern. While some subjects exhibited relatively stable TTV-DNA levels over time, others demonstrated pronounced fluctuations, including marked short-term changes within quarterly intervals. Moreover, certain individuals displayed gradual trends with either increasing or decreasing TTV-DNA loads during the entire observation period.

To assess the absolute and relative span between the lowest and highest viral loads as well as the variation relative to the median TTV-DNA level within an individual, the dynamic range (min to max), the corresponding fold change (max/min) and the coefficient of variation (SD normalized to the median) were calculated. Consistent with the mean TTV-DNA load, the dynamic range of TTV-DNA load significantly differed between subjects, ranging from 1.09 × 10^4^ cp/ml (Subject 5) to 1.25 × 10^6^ cp/ml (Subject 9). While six out of the ten subjects displayed low intra-individual variability over the observation period with fold changes <20-fold (within the previously determined range of precision of the assay) and coefficients of variation <2.5%, TTV-DNA levels fluctuated considerably in the remaining four subjects (3, 6, 8, and 9). In these individuals, markedly higher changes and coefficients of variation were observed, with 36.4-fold (1.6 log_10_) and 5.35% (Subject 3), 1523.8-fold (3.2 log_10_) and 8.91% (Subject 6), 865.4-fold (2.9 log_10_) and 1.53% (Subject 8), 673.2-fold (2.8 log_10_), and 4.75% (Subject 9). The pattern observed for long-term variation across the entire observation period was also evident in short-term fluctuations within individual quarterly intervals (3-month period). The four subjects described above showed markedly higher variability, with maximum quarterly changes of 15.6-fold (1.2 log_10_) (Subject 3), 373.7-fold (2.6 log_10_) (Subject 6), 36.5-fold (1.6 log_10_) (Subject 8), and 97.4-fold (2 log_10_) (Subject 9), whereas in the remaining subjects, maximum quarterly changes did not exceed tenfold.

The longitudinal slope was stable (|slope|≤ 0.01) for subjects 1–5 and 7, coherent with a stable mean TTV-DNA load. Subjects 6, 8, 10 showed a trend which indicates an increase in mean TTV-DNA load (slope ≥0.02), whereas subject 9 showed a trend coherent with a decrease in mean TTV-DNA load (slope ≤−0.02). No relationship was observed between the mean viral load and the magnitude of fluctuations, indicating that low viral load does not necessarily correspond to high variability, or vice versa.

To confirm these findings, an independent second cohort comprising 11 subjects was analyzed. Similar to the first cohort, none of the subjects had relevant preexisting comorbidities or were receiving iatrogenic immunosuppressive therapy. This cohort consisted of three male and eight female subjects, ranging from age 21 to 64. In contrast to the first cohort, however, blood samples were not collected at fixed quarterly intervals. The observation period ranged from 559 to 1150 days (1.5–3.1 years). The pronounced inter- and intra-individual variability in TTV-DNA load observed in the first cohort was likewise evident in this second cohort (Appendix Fig. S15). Three subjects exhibited marked fluctuations in TTV-DNA load throughout the observation period, with changes exceeding 1 log_10_ (Appendix Table S3). Maximum fluctuations of 1.3 log_10_, 1.0 log_10_, and 1.6 log_10_ were observed in subjects 11, 12, and 16, respectively, over periods of 70–90 days (~3 months).

As our PCR assay relies on a single primer/probe set, we sought to exclude the possibility that the observed variability was influenced by sequence variations within the TTV target region or by other assay-specific artifacts. To further investigate this possibility, TTV-DNA levels were quantified in samples from three subjects using two additional assays (Appendix Fig. S16). These included the assay described by Maggi et al, which targets a slightly different genomic region, and a newly available commercial TTV assay. All three assays were performed in parallel on the cobas 5800 system and demonstrated highly comparable TTV-DNA kinetics throughout the observation period. This included pronounced fluctuations in TTV-DNA load in subjects 12 and 16, as well as relatively stable TTV-DNA levels in subject 17. Collectively, these findings indicate that the observed temporal dynamics are not attributable to assay-specific artifacts and are unlikely to be explained solely by sequence variation within the TTV genome. In summary, inter- and intra-individual TTV-DNA levels varied markedly and showed a heterogeneous pattern, ranging from stable viral loads to pronounced long- and short-term fluctuations. Moreover, longitudinal trends in several subjects were observed, corresponding to either increases or decreases in mean TTV-DNA load.

## Discussion

TTV is a highly prevalent virus detectable in the majority of the human population and present in a broad range of clinical specimen types, including plasma and serum. TTV-DNA has emerged as a promising biomarker of a patient-specific immune status, especially in patients with a compromised immune system or receiving immunosuppressive therapy, such as after SOT (Haupenthal and Bond, 2023; Querido et al, 2023). In these SOT patients, inadequate adjustment of immunosuppressive therapy represents a major risk factor for severe complications, including opportunistic infections or graft rejection (Oweira et al, 2022; Pagalilauan and Limaye, 2013). Consequently, there is an increasing demand for minimally invasive, standardized monitoring strategies to support an individual and optimized immunosuppressive management. Therefore, sensitive and specific quantification of TTV-DNA is of particular clinical interest, and the processing of those large sample numbers further necessitates automated workflows to reduce costs, potential sources of error, minimize hands-on time, and enable timely analysis (Behnam Sani and Sawitzki, 2017; Cabezas et al, 2024).

In this study, we successfully adapted the previously described Maggi et al assay (Maggi et al, 2001; Spezia et al, 2024) for implementation on the cobas x800 system. While analytical precision, linearity, and matrix independence were confirmed, the comparatively low RFI resulted in reduced sensitivity for low-positive samples when applying the standard RFI_min_ threshold. Lowering this threshold improved sensitivity but increased the risk of false-positive interpretation on the cobas 5800 in particular, highlighting the importance of fluorescence signal strength for reliable automated analysis. To overcome these limitations, a novel TTV-DNA-qPCR assay (TUM-TTV-DNA-qPCR) was developed and optimized specifically for the cobas x800 platform. This assay demonstrated substantially higher RFI values, allowing the RFI_min_ threshold to be increased to 2.7 while maintaining specificity, thereby further minimizing the risk of false-positive detections. Analytical validation confirmed excellent intra- and inter-assay precision, strong linearity (*R*² up to 0.9999), absence of relevant matrix effects, and a 95% LOD of 32.3 cp/ml. This LOD is comparable or superior to the automated Maggi et al based assay (40 cp/ml (Spezia et al, 2024)), the manual Maggi et al assay (100–1000 cp/ml (Maggi et al, 2001)), and to other commercially available tests such as the manual R-GENE PCR assay (159 cp/ml (Kulifaj et al, 2018)). An inter-laboratory comparison of the individual and laboratory-specific TTV-DNA assays further demonstrated consistent detection and concordant quantification across three independent laboratories, supporting excellent robustness and reproducibility. In conclusion, a direct comparison of both assays on the cobas 5800 system confirmed superior signal intensity and improved low-level detection performance of the TUM-TTV-DNA-qPCR assay. These findings emphasize that high RFI values and a low LOD are critical for reliable, high-throughput automated testing.

Application of the assay for longitudinal assessment of TTV-DNA load in 10 individuals without iatrogenic immunosuppression revealed substantial intra-individual variability in some subjects. Both short-term fluctuations (quarterly) and directional trends were observed, independent of baseline TTV-DNA levels. Importantly, no consistent relationship between mean TTV-DNA load and variability could be identified. In particular, some individuals maintained stable median levels despite short-term fluctuations, whereas others exhibited progressive increases or decreases over time. The definition of a single value or mathematical metric that meaningfully captures and compares these fluctuations is challenging, as variability has to be interpreted in the context of the individual’s average TTV-DNA load. Especially high variations in TTV-DNA were observed in four subjects (3, 6, 8, 9), with fold changes between 36.36-fold (1.56 log_10_) and 1523.81-fold (3.18 log_10_). These subjects not only exhibited high TTV-DNA load variability over the entire observation period but also displayed the highest maximum quarterly changes within a 3-month interval between 15.6-fold (1.19 log_10_) and 373.7-fold (2.57 log_10_). The fluctuations observed in this cohort could also be observed in a second independent cohort of 11 subjects, confirming our findings. Furthermore, assay-specific artifacts and methodological biases were assessed by comparing TTV-DNA kinetics obtained with two additional assays, which exhibited highly comparable trajectories and fluctuations of similar magnitude. Consequently, the observed variability is unlikely to be explained exclusively by sequence variation or mutations within the target region, as consistent fluctuation patterns were observed across all three assays. In patients with iatrogenic immunosuppression after SOT, a marked increase in TTV-DNA load following initiation of immunosuppressive therapy was described by previous reports (Cabezas et al, 2024; Fernández-Ruiz et al, 2019; Haupenthal and Bond, 2023; Querido et al, 2023). These studies have demonstrated that increases in TTV-DNA loads can become detectable within days after initiation of immunosuppressive therapy and persist during subsequent follow-up periods. However, due to the considerable heterogeneity in study designs and methodological approaches, there are currently no standardized reference values for TTV-DNA loads during immunosuppression. For example, an average increase in TTV-DNA load of more than 2 log_10_ cp/ml from baseline within the first three months after kidney transplantation has been described in 66 transplant recipients (Solis et al, 2019). Moreover, a TTV viral load >1 × 10^6^ cp/ml, or within a range of 1 × 10^6^ to 1 × 10^8^ cp/ml, has been proposed as an optimal window to minimize the risk of graft rejection and infection in kidney transplant recipients (Doberer et al, 2021; Doberer et al, 2020; Strassl et al, 2019). Conversely, lower TTV-DNA levels, such as <4.2 log_10_ cp/ml at one month post-transplantation, have been associated with an increased risk of rejection (Solis et al, 2019). Furthermore, pronounced fluctuations in TTV levels have been associated with infectious events, with viral load variations greater than 2.65 log_10_ cp/ml in the first month predicting the development of infectious events (Querido et al, 2023). Similar dynamics have been observed in heart transplant settings, with TTV-DNA levels increasing markedly after heart transplantation from ~4.4 log_10_ cp/ml pre-transplant to 9.39 log_10_ cp/ml thereafter (Uyanik-Uenal et al, 2020). Moreover, several studies have demonstrated that each 1 log_10_ increase in TTV-DNA load is associated with a significant reduction in the odds of rejection following kidney transplantation (Doberer et al, 2021; Doberer et al, 2020; Schiemann et al, 2017; Strassl et al, 2019; van Rijn et al, 2023). In a cohort of lung transplant patients, it was further demonstrated that the peak TTV-DNA load after initiation of immunosuppression is, on average, independent of the baseline TTV-DNA load. Instead, the time required to reach this median peak level of 9.4 log_10_ cp/ml appears to be the primary variable (Görzer et al, 2015). In summary, these studies report an increase in TTV load following SOT and iatrogenic immunosuppression; however, the extent of TTV load increase, baseline characterization, and proposed optimal TTV-DNA values differ between studies, so that the definition of a universal threshold proves to be critical. Eventually, a universal threshold may not be applicable across all clinical settings, as the type and intensity of immunosuppression can differ substantially. Consequently, the impact on TTV-DNA load is also likely to vary. Therefore, interpreting TTV levels within the specific clinical setting is probably more appropriate and meaningful than applying a single universal cutoff. Given our data, defining an absolute universal threshold for TTV-DNA levels as described in kidney transplants indicative of adequate immunosuppression remains challenging, as those values must always be interpreted in the context of the individual baseline viral load. This is particularly relevant as some patients may exhibit baseline TTV-DNA levels of ~1 × 10^6^ cp/ml, or may naturally fluctuate within this range independent of any iatrogenic immunosuppression. For highly immunosuppressed lung transplant recipients, this may not represent a major limitation. As demonstrated in our study, mean TTV-DNA levels differed by up to 1.6 log_10_ between subjects. Notably, individuals such as subject 9 reached maximum TTV-DNA levels of 1.25 × 10^6^ cp/ml over a 39-month period despite the absence of iatrogenic immunosuppression, thereby falling within the proposed “optimal” range described in kidney transplant cohorts. This study further shows that substantial fluctuations in TTV-DNA levels can occur independently of immunosuppressive therapy in a subset of subjects. In some non-immunosuppressed individuals, the overall intra-individual variation reached up to 3.2 log_10_, while even subjects with comparatively low overall variability exhibited changes of up to 1.2 log_10_ over 30 months. In addition, short-term variations within quarterly intervals exceeded 1 log_10_ in five subjects. In a setting where differences as small as 1 log_10_ may have clinical relevance, such natural variability must be taken into account. Therefore, interpretation of TTV-DNA dynamics during maintenance immunosuppression requires careful consideration. This is particularly relevant in kidney or heart transplant recipients, where the proposed thresholds are significantly lower than those reported for lung transplant recipients. While early post-transplantation increases in TTV-DNA are often pronounced, long-term changes are likely to occur more gradually. Reliable clinical application of TTV as a biomarker thus presupposes knowledge of patient-specific baseline variability and supports the use of relative, rather than absolute, and clinical setting-specific thresholds.

Our findings demonstrate that TTV-DNA exhibits both short-term and long-term intra-individual dynamics even in the absence of immunosuppressive therapy. From the data available in this study, it cannot be conclusively determined whether the observed changes reflect modulation of immune function by other factors affecting the immune system besides immunosuppressive medication, or whether they instead represent individual intrinsic variability in viral replication independent of immune alterations. However, this study provides one of the first long-term data on TTV-DNA loads in non-immunosuppressed individuals, and this distinction is critical for the validation of TTV-DNA as a biomarker, as therapy-guided decision-making assumes that observed changes predominantly reflect alterations in immune status. Generally, TTV-DNA load shows greater fluctuations in children, likely due to ongoing immune system development and frequent exposure to new infections with other members in the anellovirus family (McElvania TeKippe et al, 2012; Väisänen et al, 2022). In contrast, the limited studies in adults have reported relatively stable TTV viremia over time (Kaczorowska et al, 2022). However, these differences are likely explained by variations in study design. The limited number of longitudinal studies currently available either included only very small cohorts (e.g., two individuals) or assessed samples collected at widely spaced time points (~10 years apart). Consequently, the short-term fluctuations observed in ~30% of subjects in our study would largely have remained undetected and therefore could not have been described in those investigations. Nevertheless, longitudinal data remain scarce, and further studies are needed to fully understand individual and age-dependent dynamics of TTV-DNA loads. Since TTV and other anelloviruses exhibit extensive genomic heterogeneity, individuals are infected with a variety of viral variants and genotypes. It is proposed that this individual “anellome” remains largely stable during adulthood (Kaczorowska et al, 2022; Timmerman et al, 2024), therefore, new infections with different viral variants are unlikely to underlie the observed interindividual fluctuations, although the data remains scarce. Interestingly, longitudinal trajectories in several individuals appear to exhibit structured fluctuation patterns, characterized by alternating phases of increase and decrease rather than purely random variation. If such recurring patterns reflect underlying modulation of immune function, they may have important clinical relevance, as transient phases of relative immune activation or suppression could influence susceptibility to infections or other immune-related events. Future studies must therefore investigate potential temporal determinants of these fluctuations, including seasonal variation, intercurrent infections, change in medication, and other environmental or host-related factors, to determine whether TTV-DNA load dynamics can be reliably linked to changes in immune status.

In summary, this study establishes a fully automated high-throughput TTV-DNA-qPCR assay with robust analytical performance and inter-laboratory reproducibility. At the same time, the intra-individual variability in TTV-DNA levels observed in a subset of subjects highlights the need for individualized baseline characterization and cautious longitudinal interpretation for the reliable clinical implementation of TTV as a biomarker of immune status. Larger prospective cohorts and comprehensive statistical modeling will be required to define clinically actionable thresholds and to distinguish physiological variability from therapy-related immune modulation.

## Methods

**Table:** Reagents and tools table

Reagent/resource	Reference or source	Identifier or catalog number
**Recombinant DNA**		
10-beta Competent *E. coli* (High Efficiency)	New England Biolabs	C3019H
**Oligonucleotides and other sequence-based reagents**		
PCR Primers	This study	Appendix Table S2
**Chemicals, enzymes and other reagents**		
Agarose, universal, DNA pure grade	VWR International, LLC	732–2789
Aqua ad iniectabilia	B. Braun Melsungen AG	3000910
Cobas omni lysis reagent	Roche Holding AG	06997538190
Cobas omni MGP reagent	Roche Holding AG	06997546190
Cobas omni specimen diluent	Roche Holding AG	06997511190
Cobas omni wash reagent	Roche Holding AG	06997503190
Cobas buffer negative control kit	Roche Holding AG	09051953190
FastDigest Green Buffer (10X)	Thermo Fisher Scientific Inc.	B72
Fetal Bovine Serum (FBS)	Gibco	A5670701
GeneRuler DNA Ladder Mix, ready-to-use	Thermo Fisher Scientific Inc.	SM0334
ROTI GelStain	CARL ROTH	3865.1
T4 DNA Ligase Buffer (10X)	Thermo Fisher Scientific Inc.	B69
FastAP Thermosensitive Alkaline Phosphatase (1 U/μl)	Thermo Fisher Scientific Inc.	EF0651
FastDigest BpiI (Class IIs) Thermo Fisher	Thermo Fisher Scientific Inc.	FD1014
T4 DNA Ligase (5 U/μl)	Thermo Fisher Scientific Inc.	EL0011
T4 Polynucleotide Kinase	New England Biolabs	M0201S
Universal Transport Medium for viruses	COPAN ITALIA	305 C
SOC Outgrowth Medium	New England Biolabs	B9020S
50x Tris-Acetate-EDTA-Electrophoresis buffer	In-house made	
**Software**		
Cobas omni utility channel optimization tool v5.1	Roche Holding AG	
Cobas omni utility channel RFID tool 1.0.0.27	Roche Holding AG	
Eurofins qPCR Primer & Probe Design Tool	Eurofins Genomics	
Excel	Microsoft	
GenBank	NLM	
GenScript Online PCR Primers Design Tool	GenScript	
GraphPad Prism	GraphPad Software	
LightCycler 480 SW UDF 2.1.0	Roche Holding AG	
NCBI blast	NLM	
Primer3web version 4.1.0 Tool	Primer3	
SnapGene	GSL Biotech LLC	
**Other**		
Cobas omni optimization kit	Roche Holding AG	07731663190
Cobas omni utility channel reagent kit	Roche Holding AG	09052011190
GeneJET Gel Extraction kit	Thermo Scientific	K0691
LightCycler 480 high-resolution melting master	Roche Holding AG	04909631001
Plasmid Plus Midi Kit	QIAGEN	12945
STARMag 96 ProPrep (Plate type)	Seegene Inc.	EX00009P
STARMag 96 ProPrep (Tube type)	Seegene Inc.	EX00009T
96 Well Lightcycler plate	SARSTEDT AG & Co. KG	72.1982.202
Cobas z 480 Analyzer	Roche Holding AG	
Cobas 5800	Roche Holding AG	
NanoDrop™ One Microvolume UV Spectrophotometer	Thermo Fisher Scientific Inc.	ND-ONE-W
PCR Plate centrifuge II, 230 V	VWR International, LLC.	521–3497 P

### Preparation of patient samples

The study was conducted in accordance with the ethical principles of the World Medical Association Declaration of Helsinki and the Belmont Report. The use of residual diagnostic serum and EDTA plasma samples for this study was reviewed and approved by the Ethics Committee of the TUM University Hospital (reference number 2024-410-S-CB), which issued a positive vote and raised no objections to the conduct of the study. Under the approved study protocol, individual informed consent was not required. Residual samples were pseudonymized, and no blinding was applied during data collection or analysis. The used residual serum and EDTA plasma samples from routine clinical diagnostics had been centrifuged at 3000×*g* for 15 min and stored at −20 °C as part of routine clinical procedures. If needed, sample dilutions were prepared using bidistilled water with 20% FCS. Swab media were spiked with TTV-positive plasma to generate positive swabs. Based on their measured concentrations, samples were classified as high-positive (3 × 10^5^–3 × 10^6^), medium-positive (6 × 10^3^–1 × 10^5^), or low-positive (5–2 × 10^3^).

### Oligonucleotide design

For the design of TTV-specific oligonucleotides, complete human TTV genome sequences were retrieved from the NCBI Virus database on 19.06.2024 using the query “Taxonomy ID: 68887 AND complete genome”. Only sequences annotated as human TTV were included, whereas sequences assigned to Torque teno mini virus (TTMV), Torque teno midi virus (TTMDV), and other non-human anelloviruses were excluded based on their taxonomic annotation. Prior to analysis, sequences were filtered to retain only complete or near-complete genomes with a minimum sequence length of 3100 bp. Additionally, only high-quality sequences derived from Homo sapiens were included, while metagenomic, environmental, and sequences containing extensive ambiguous nucleotides (>1–2% ambiguous bases (N)) were excluded. Also, redundant entries, duplicate sequences, and laboratory constructs were excluded. Where available, RefSeq and well-annotated genome entries were preferred to ensure consistency and reliability of the dataset. After filtering, a total of 631 unique human TTV genome sequences were retained for further analysis.

Multiple sequence alignment was performed using the MAFFT algorithm as implemented in SnapGene on full-length genome sequences. Following alignment, a consensus sequence was generated, and nucleotide conservation was calculated for each genomic position. Consistent with the known genetic diversity of TTV, only a single highly conserved region was identified within the untranslated region (UTR). To facilitate primer and probe design, a consensus sequence with a conservation threshold of >75% nucleotide identity was generated, and candidate oligonucleotides were designed within the conserved UTR region.

For primer and probe design, the online in silico tools Primer3, GenScript, and Eurofins were used. The Roche guidelines for primer and probe design for the Cobas 5800 system were followed (Table 2). Proposed oligonucleotide sequences were then manually reviewed to maximize coverage across the consensus sequence. Potential sequence variants at positions lacking fully conserved nucleotides were considered, and variable sites were avoided at critical primer binding regions.

**Table 2:** Key design criteria for oligonucleotide design for the cobas x800 system.

Requirements	Primer	Probe	Amplicon
GC content	35–65% (equal)	45–65%	35–65%
Length	18–30 bp	18–37 bp	60–200 bp
Melting temperature	60– 65 °C	5 °C higher than primers	
Secondary structure	None	None	
Self-complementary	No	No	
Others	No four equal nt	No four equal nt No G at 5’ end	

### Extraction of viral DNA

#### Semi-automated extraction

For semi-automated extraction of viral nucleic acids, the STARMag 96 ProPrep Kit (Seegene) was utilized. The procedure was performed on the SEEPREP32 device (Seegene) according to the manufacturer’s instructions. After extraction, eluates (~100 μl) were stored at –20 °C until further use.

#### Automated extraction

In the fully automated workflow of the cobas x800 system (Roche), TTV-DNA-positive and negative samples were automatically extracted with the cobas omni lysis reagent on the cobas 5800 and then automatically used for PCR detection.

### Performing qPCRs on several devices

#### Semi-automated qPCR on the cobas z 480/LC 480 II

The semi-automated qPCR was performed using TaqMan qPCR on a cobas z 480/LC 480 II. For detection, the cobas omni optimization kit (Roche) was utilized according to the manufacturer’s instructions. Unless otherwise stated, the PCR master mix contained 0.4 µM of both forward and reverse primers and 0.05 µM of the probe. The reaction conditions are described in Table 3. A quantification standard was generated by cloning a TTV target sequence into a plasmid and used for inter-assay quantification comparison.

**Table 3:** Reaction conditions for Maggi et al./TUM-TTV-DNA-qPCR on cobas z 480/LC 480 II.

		Temperature	Time
**UNG-incubation**		50 °C	120 s
		94 °C	5 s
**Generic UC program**	Pre-PCR program	55 °C	120 s
		60 °C	360 s
		65 °C	240 s
	First measurement (5 cycles)	95 °C	5 s
		55 °C	30 s*
	Second measurement (45 cycles)	91 °C	5 s
		58 °C	25 s*
**Cooling**		40 °C	60 s

^*^Acquisition mode: Single.

#### Full-automated qPCR on the cobas x800

The fully automated cobas x800 qPCR was performed on the cobas 5800 (Roche). The Cobas Omni Utility Channel Reagent Kit was filled with TTV-specific primers (0.4 µM) and probe (0.05 µM) according to the manufacturer’s instructions. In addition to the in-house PCR assays, the commercially available UC-TIB-TTV assay was performed according to the manufacturer’s instructions. The cobas buffer negative control kit was used as a negative control. Samples were transferred into secondary tubes and loaded onto the cobas 5800, which required 500 µl of sample for extraction, not including dead volume. The PCR conditions were similar to those on the cobas z 480/LC 480 II, as described in Table 3.

#### Digital PCR (dPCR) on the QIAcuity One 5plex

To generate a quantitative TTV standard, dPCR on the QIAcuity One 5plex (QIAGEN) was performed. The master mix for dPCR was prepared with the QIAcuity Probe PCR Kit (QIAGEN), with primer concentrations of 0.8 µM and a probe concentration of 0.4 µM, according to the manufacturer’s instructions. dPCR conditions are described in Table 4.

**Table 4:** Reaction conditions for Maggi et al./TUM-TTV-DNA dPCR on QIAcuity One 5plex.

	Cycles	Temperature	Time
**Preincubation**	1	95 °C	2 min
**1. PCR-step**	45	95 °C	15 s
		58 °C	30 s
Measurement			
**2. PCR-step**	10	95 °C	15 s
		58 °C	30 s
Measurement			
**3. PCR-step**	10	95 °C	15 s
		58 °C	30 s
Measurement			

#### High-resolution melting PCR

High-resolution melting (HRM) analysis was performed to evaluate PCR amplicons and oligo dimer formation using the LightCycler High Resolution Melting Master (Roche) on the cobas z 480/LC 480 II system according to the manufacturer’s instructions. The PCR master mix contained 0.4 µM of both forward and reverse primers. The reaction conditions are described in Table 5.

**Table 5:** Reaction conditions for Maggi et al./TUM-HRM PCR on cobas z 480/LC 480 II.

	Cycles	Temperature	Time
**Preincubation**	1	95 °C	10 min
**Amplification**	45	95 °C	10 s
		58 °C	15 s
		72 °C	15 s
**Melting curve**	1	95 °C	1 min
		40 °C	1 min
		64–95 °C	1 °C/s
**Cooling**	1	40 °C	10 s

### Plasmid construction

Custom DNA oligonucleotides encoding variations in the TTV-DNA PCR target sequence were designed, annealed, and phosphorylated prior to insertion into a pCAGGS-derived plasmid backbone by restriction enzyme cloning. Competent *E. coli* were transformed with the ligation products and selected on ampicillin-containing agar plates. Single colonies were expanded, plasmid DNA was purified, and sequence integrity was verified by Sanger sequencing.

### Statistical and mathematical analyses

Statistical analyses were performed using GraphPad Prism (version 10.3.1), unless otherwise stated. Paired t-tests (Appendix Table S4) were used to assess differences between paired measurements. Bland–Altman analyses were performed to evaluate the agreement between PCR assays. Two-way analysis of variance (ANOVA) was used to assess the effects of multiple variables and their interactions, where appropriate. Linear regression analyses were performed to assess assay linearity, with coefficients of determination (*R*²), regression equations, and slopes being determined. The LoD was estimated by probit analysis using MedCalc.

## Supplementary information

Appendix

Peer Review File

Source data Fig. 1

Source data Fig. 2

Source data Fig. 3

Source data Fig. 4

Expanded View Figures

## Data Availability

This study includes no data deposited in external repositories. The source data of this paper are collected in the following database record: biostudies:S-SCDT-10_1038-S44321-026-00540-3.
