# Supplementary material for: A novel Torque teno virus qPCR assay reveals longitudinal viral load variability among non-immunosuppressed subjects
Source: EMBO Mol Med. 2026 Oct 8;18(11):4618–42. doi: 10.1038/s44321-026-00540-3 (PMC13748663; doi:10.1038/s44321-026-00540-3)
Supplement: Supplementary file 1 [file 44321_2026_540_MOESM1_ESM.pdf]

Appendix:

Table of content:

Appendix Figure S1: Comparison of annealing/elongation temperatures.	2
Appendix Figure S 2: HRM analysis comparing TTV-specific primers.	2
Appendix Figure S 3: Optimization of primer and probe concentration of the Maggi et al. n/m primers.	3
Appendix Table S 1: All sequences used for generation of the TTV consensus sequence.	4
Appendix Figure S 4: Consensus sequence map of the TTV genome.	7
Appendix Figure S 5: Conserved sequence of 172 bp within the UTR of the TTV genome.	7
Appendix Table S 2: All TTV specific oligos used for PCR.	8
Appendix Figure S 6: HRM analysis comparing TTV-specific primers.	9
Appendix Figure S 7: Comparison of modified TUM 1 primers for TTV detection.	9
Appendix Figure S 8: Optimization of primer and probe concentration of the TUM 1 2m/m primers.	10
Appendix Figure S 9: TTV consensus sequence with 95 % sequence homology and bound TUM 1 primers/probe.	10
Appendix Figure S 10: Different constructs of the TTV target sequence of TUM 1 primer/probe.	11
Appendix Figure S 11: Inter-laboratory comparison of TTV PCR assays.	11
Appendix Figure S 12: Comparison of Ct values for selected samples.	12
Appendix Figure S 13: Comparison of two TTV-DNA qPCR assays.	12
Appendix Figure S 14: Amplification curve of a false positive TTV sample.	13
Appendix Figure S 15: Longitudinal TTV-DNA levels in patients without iatrogenic immunosuppression.	14
Appendix Table S 3: Mathematical characterization of longitudinal variability in TTV-DNA loads in subjects (subject 11-21) with no iatrogenic-immunosuppression.	15
Appendix Figure S 16: Comparison of longitudinal TTV-DNA levels.	15
Appendix Table S 4: Exact p-values obtained from all paired t-tests performed for the statistical comparisons presented in the figures.	16

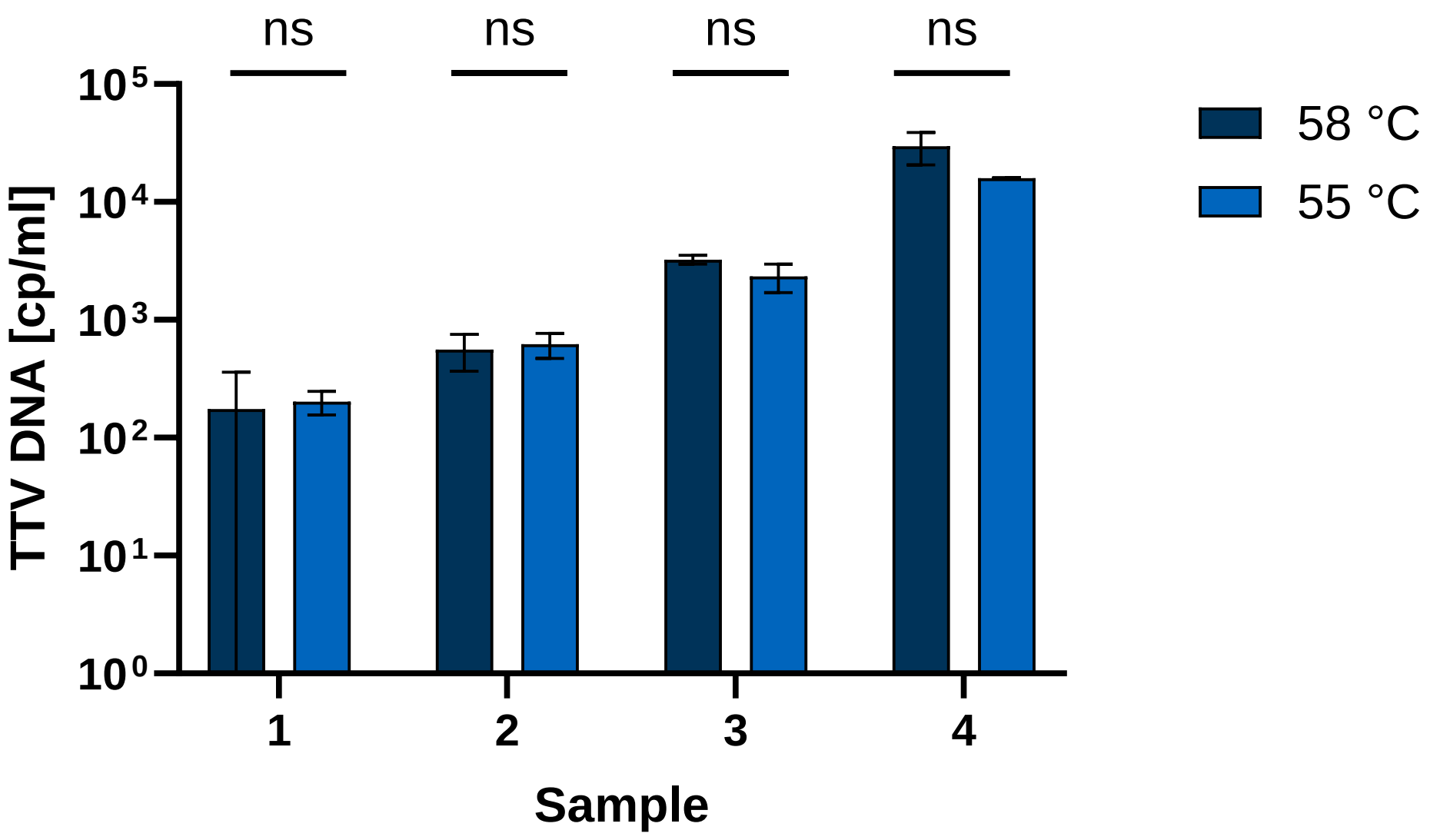

Appendix Figure S 1: Comparison of annealing/elongation temperatures.
The original described 55 °C and the proposed temperature of 58 °C for the cobas x800 system were compared for four different samples. Between all samples no significant differences were observed. Data are shown as mean ± SD of triplicate measurements (n=3). Statistical analysis was performed using a paired t-test. All comparisons were non-significant (ns, $P > 0.05$). Exact p-values are described in Appendix Table S 4.

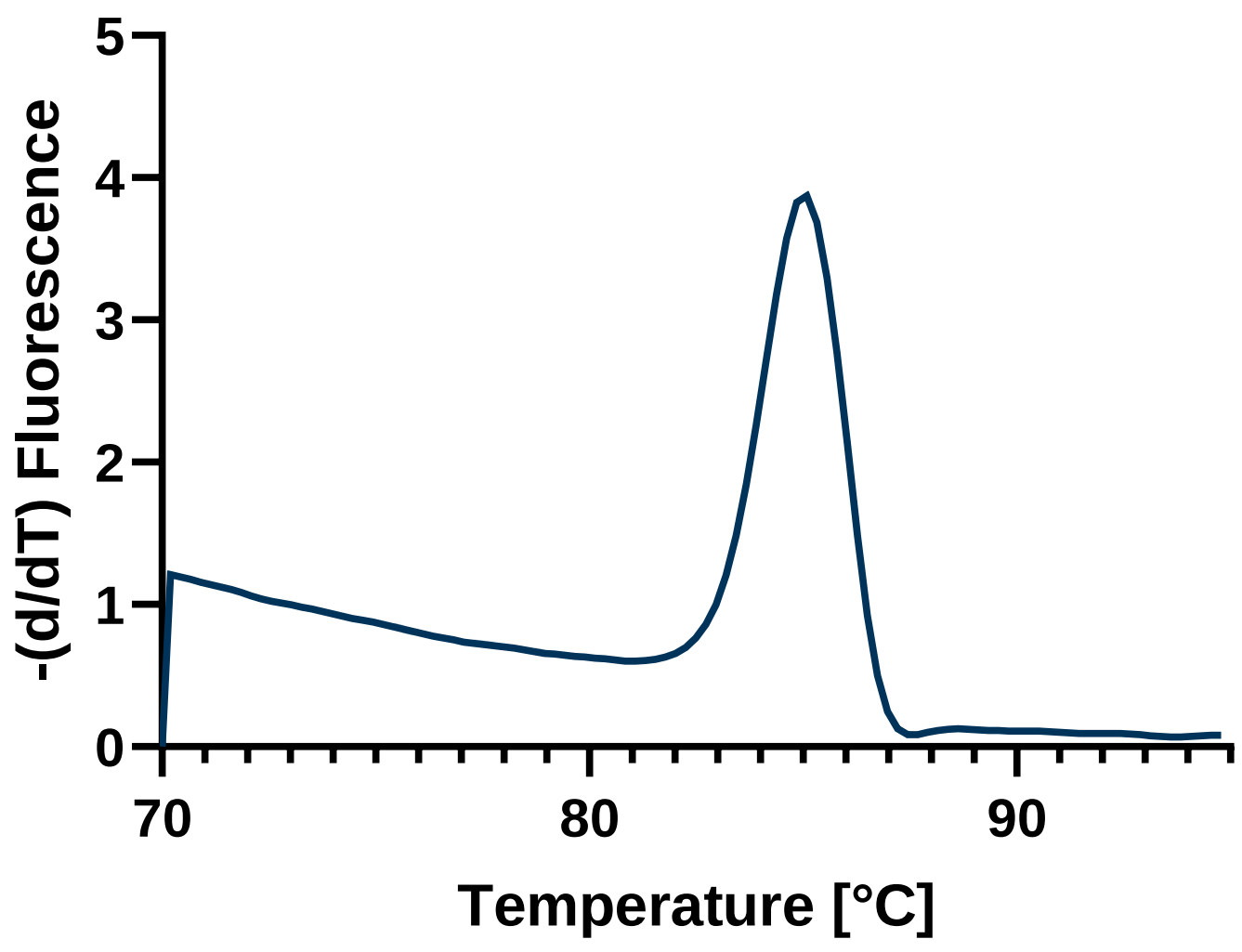

Appendix Figure S 2: HRM analysis comparing TTV-specific primers.
Melting profiles for *Maggi et al.* Primer set showing distinct melting temperatures and peak shapes. Indicating varying levels of specificity and potential binding efficiency, with distinct melting peaks corresponding to each pri-mer set. Primers showing just the specific TTV amplicon peak.

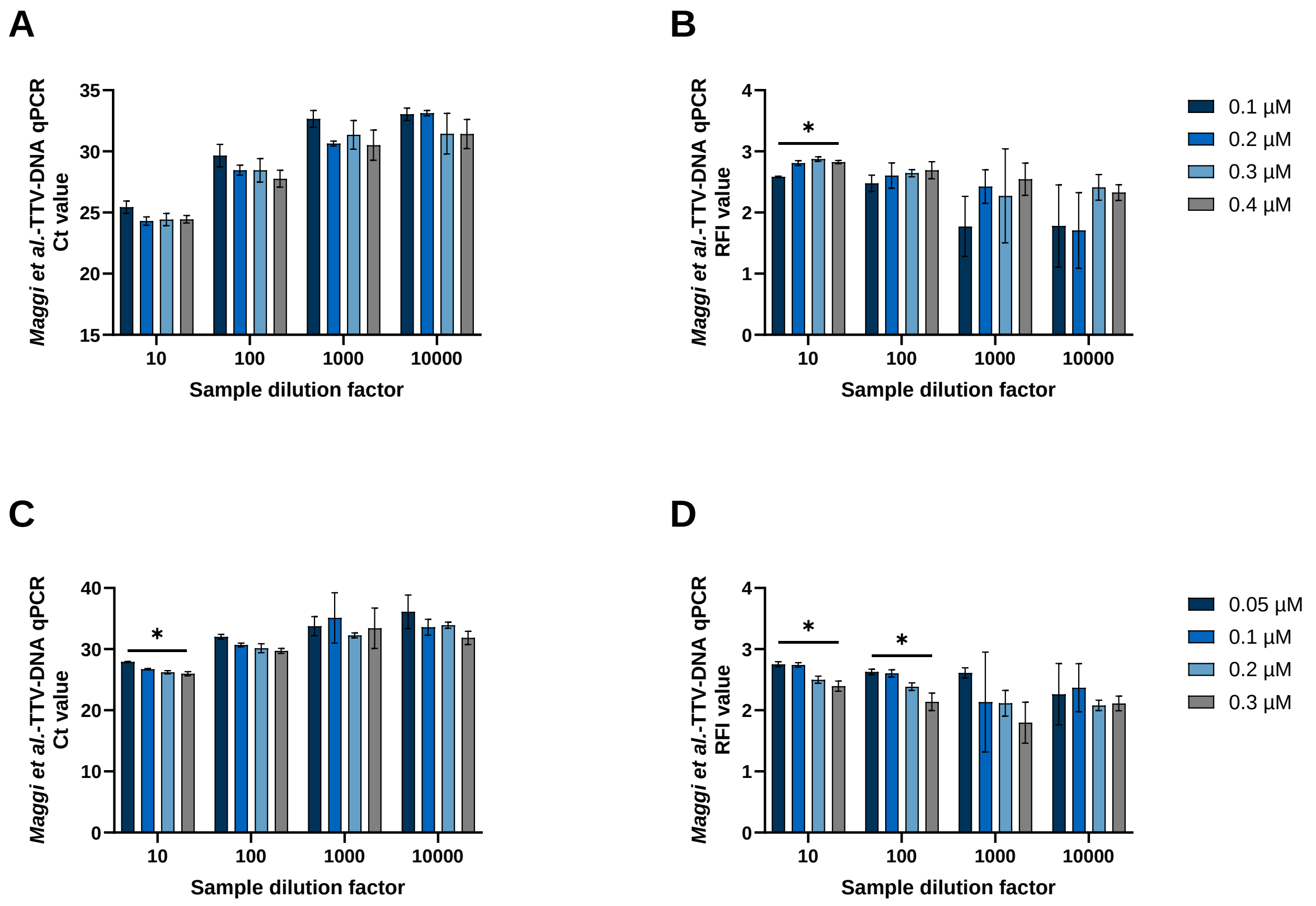

Appendix Figure S 3: Optimization of primer and probe concentration of the *Maggi et al.* n/m primers.

Evaluation of Ct value (A, C) and RFI (B, D) four different primer concentrations (A, B): 0.4 µM, 0.3 µM, 0.2 µM and 0.1 µM and four different probe concentrations (C, D): 0.05 µM, 0.1 µM, 0.2 µM and 0.3 µM. Serial dilution of a TTV positive plasma sample was measured. Data are shown as mean ± SD of triplicate measurements (n=3). Statistical significance was assessed using a paired t-test. $P < 0.05$ is indicated by * and all comparisons not indicated were considered non-significant ($P > 0.05$). Exact p-values are described in Appendix Table S 4.

Appendix Table S 1: All sequences used for generation of the TTV consensus sequence.

gb|MN766098.1|	gb|MN766908.1|	gb|MN765748.1|	gb|MN766618.1|	gb|MN767385.1|
gb|MN766045.1|	gb|MN766923.1|	gb|MN766740.1|	gb|MN766516.1|	gb|MN767418.1|
gb|MN765977.1|	gb|MN766944.1|	gb|MN766742.1|	gb|MN765902.1|	gb|MN767265.1|
gb|MN766031.1|	gb|MN766943.1|	gb|MN766489.1|	gb|MN765866.1|	gb|MN767402.1|
gb|MN766085.1|	gb|MN766882.1|	gb|MN766417.1|	gb|MN765870.1|	gb|MN767508.1|
gb|MN766058.1|	gb|MN766852.1|	gb|MN766390.1|	gb|MN765871.1|	gb|MN767558.1|
gb|MN766002.1|	gb|MN766851.1|	gb|MN766382.1|	gb|MN765911.1|	gb|MN767462.1|
gb|MN766040.1|	gb|MN766916.1|	gb|MN766419.1|	gb|MN765893.1|	gb|MN767323.1|
gb|MN766023.1|	gb|MN766904.1|	gb|MN766413.1|	gb|MN765922.1|	gb|MN767370.1|
gb|MN766001.1|	gb|MN766902.1|	gb|MN766561.1|	gb|MN765934.1|	gb|MN767366.1|
gb|MN766067.1|	gb|MN766891.1|	gb|MN766577.1|	gb|MN765939.1|	gb|MN767435.1|
gb|MN766079.1|	gb|MN766274.1|	gb|MN766608.1|	gb|MN765883.1|	gb|MN767675.1|
gb|MN766100.1|	gb|MN766237.1|	gb|MN766746.1|	gb|MN765915.1|	gb|MN767676.1|
gb|MN766026.1|	gb|MN766173.1|	gb|MN766700.1|	gb|MN765932.1|	gb|MN766969.1|
gb|MN766027.1|	gb|MN766350.1|	gb|MN766725.1|	gb|MN765941.1|	gb|MN766980.1|
gb|MN766077.1|	gb|MN766219.1|	gb|MN766690.1|	gb|MN765873.1|	gb|MN766966.1|
gb|MN766452.1|	gb|MN766247.1|	gb|MN766418.1|	gb|MN765910.1|	gb|MN767476.1|
gb|MN766449.1|	gb|MN766197.1|	gb|MN766430.1|	gb|MN765909.1|	gb|MN767309.1|
gb|MN766476.1|	gb|MN766282.1|	gb|MN766439.1|	gb|MN766101.1|	gb|MN767471.1|
gb|MN766106.1|	gb|MN766236.1|	gb|MN766426.1|	gb|MN765947.1|	gb|MN767473.1|
gb|MN766121.1|	gb|MN766202.1|	gb|MN766603.1|	gb|MN765933.1|	gb|MN767724.1|
gb|MN766111.1|	gb|MN766235.1|	gb|MN766620.1|	gb|MN766147.1|	gb|MN767815.1|
gb|MN766128.1|	gb|MN766183.1|	gb|MN766601.1|	gb|MN766143.1|	gb|MN767243.1|
gb|MN766118.1|	gb|MN766357.1|	gb|MN766610.1|	gb|MN766148.1|	gb|MN767466.1|
gb|MN766114.1|	gb|MN766303.1|	gb|MN766621.1|	gb|MN765775.1|	gb|MN767467.1|
gb|MN766109.1|	gb|MN766228.1|	gb|MN766563.1|	gb|MN765825.1|	gb|MN767465.1|
gb|MN766120.1|	gb|MN766304.1|	gb|MN766622.1|	gb|MN765818.1|	gb|MN767535.1|
gb|MN766129.1|	gb|MN766329.1|	gb|MN765766.1|	gb|MN765772.1|	gb|MN767469.1|
gb|MN766948.1|	gb|MN766180.1|	gb|MN766628.1|	gb|MN765838.1|	gb|MN767464.1|
gb|MN766871.1|	gb|MN766263.1|	gb|MN766612.1|	gb|MN765831.1|	gb|MN767590.1|
gb|MN766869.1|	gb|MN766264.1|	gb|MN765726.1|	gb|MN765837.1|	gb|MN767686.1|
gb|MN766926.1|	gb|MN766187.1|	gb|MN765691.1|	gb|MN765797.1|	gb|MN767446.1|
gb|MN766941.1|	gb|MN766302.1|	gb|MN766578.1|	gb|MN765793.1|	gb|MN767625.1|
gb|MN766937.1|	gb|MN766268.1|	gb|MN766695.1|	gb|MN765804.1|	gb|MN767574.1|
gb|MN766927.1|	gb|MN766233.1|	gb|MN766655.1|	gb|MN765839.1|	gb|MN767665.1|
gb|MN766913.1|	gb|MN766208.1|	gb|MN766682.1|	gb|MN765808.1|	gb|MN767452.1|
gb|MN766896.1|	gb|MN766281.1|	gb|MN766717.1|	gb|MN765796.1|	gb|MN767460.1|
gb|MN766903.1|	gb|MN766825.1|	gb|MN766604.1|	gb|MN765836.1|	gb|MN767602.1|
gb|MN766894.1|	gb|MN766797.1|	gb|MN766564.1|	gb|MN765827.1|	gb|MN767609.1|
gb|MN766865.1|	gb|MN766829.1|	gb|MN766543.1|	gb|MN765794.1|	gb|MN767608.1|
gb|MN766859.1|	gb|MN766788.1|	gb|MN766544.1|	gb|MN767773.1|	gb|MN767286.1|
gb|MN766931.1|	gb|MN766845.1|	gb|MN766565.1|	gb|MN768005.1|	gb|MN767695.1|
gb|MN766942.1|	gb|MN766104.1|	gb|MN766532.1|	gb|MN767511.1|	gb|MN767282.1|
gb|MN766951.1|	gb|MN765686.1|	gb|MN766493.1|	gb|MN767573.1|	gb|MN767632.1|
gb|MN766881.1|	gb|MN765707.1|	gb|MN765736.1|	gb|MN767870.1|	gb|MN767455.1|
gb|MN766932.1|	gb|MN765730.1|	gb|MN766583.1|	gb|MN767871.1|	gb|MN767624.1|
gb|MN766857.1|	gb|MN765768.1|	gb|MN766579.1|	gb|MN767406.1|	gb|MN767651.1|
gb|MN766914.1|	gb|MN765722.1|	gb|MN766495.1|	gb|MN767483.1|	gb|MN767528.1|
gb|MN766892.1|	gb|MN765744.1|	gb|MN766530.1|	gb|MN767380.1|	gb|MN767576.1|

<table>
<tr><td>gb|MN768249.1|</td><td>gb|MN768529.1|</td><td>gb|MN768503.1|</td><td>gb|MN768386.1|</td><td>gb|MN765339.1|</td></tr>
<tr><td>gb|MN768214.1|</td><td>gb|MN768388.1|</td><td>gb|MN768430.1|</td><td>gb|MN768489.1|</td><td>gb|MN765505.1|</td></tr>
<tr><td>gb|MN768180.1|</td><td>gb|MN768501.1|</td><td>gb|MN768512.1|</td><td>gb|MN768365.1|</td><td>gb|MN765512.1|</td></tr>
<tr><td>gb|MN768175.1|</td><td>gb|MN768319.1|</td><td>gb|MN768427.1|</td><td>gb|MN768321.1|</td><td>gb|MN765480.1|</td></tr>
<tr><td>gb|MN768173.1|</td><td>gb|MN768492.1|</td><td>gb|MN768563.1|</td><td>gb|MN768482.1|</td><td>gb|MN765195.1|</td></tr>
<tr><td>gb|MN768183.1|</td><td>gb|MN768305.1|</td><td>gb|MN768446.1|</td><td>gb|MN768347.1|</td><td>gb|MN765250.1|</td></tr>
<tr><td>gb|MN768201.1|</td><td>gb|MN768444.1|</td><td>gb|MN768332.1|</td><td>gb|MN768445.1|</td><td>gb|MN765260.1|</td></tr>
<tr><td>gb|MN768188.1|</td><td>gb|MN768495.1|</td><td>gb|MN768374.1|</td><td>gb|MN768411.1|</td><td>gb|MN765254.1|</td></tr>
<tr><td>gb|MN768199.1|</td><td>gb|MN768461.1|</td><td>gb|MN768435.1|</td><td>gb|MN768339.1|</td><td>gb|MN765255.1|</td></tr>
<tr><td>gb|MN768221.1|</td><td>gb|MN768308.1|</td><td>gb|MN768285.1|</td><td>gb|MN768518.1|</td><td>gb|OP168886.1|</td></tr>
<tr><td>gb|MN768217.1|</td><td>gb|MN768449.1|</td><td>gb|MN768406.1|</td><td>gb|MN768284.1|</td><td>gb|MN765674.1|</td></tr>
<tr><td>gb|MN768229.1|</td><td>gb|MN768434.1|</td><td>gb|MN768404.1|</td><td>gb|MN768487.1|</td><td>gb|MN765675.1|</td></tr>
<tr><td>gb|MN768559.1|</td><td>gb|MN768425.1|</td><td>gb|MN768419.1|</td><td>gb|MN768340.1|</td><td>gb|MN765609.1|</td></tr>
<tr><td>gb|MN768211.1|</td><td>gb|MN768416.1|</td><td>gb|MN768486.1|</td><td>gb|MN768322.1|</td><td>gb|MN765273.1|</td></tr>
<tr><td>gb|MN768186.1|</td><td>gb|MN768307.1|</td><td>gb|MN768465.1|</td><td>gb|MN768371.1|</td><td>gb|MN765272.1|</td></tr>
<tr><td>gb|MN768219.1|</td><td>gb|MN768473.1|</td><td>gb|MN768393.1|</td><td>gb|MN768458.1|</td><td>gb|MN765309.1|</td></tr>
<tr><td>gb|MN768182.1|</td><td>gb|MN768381.1|</td><td>gb|MN768498.1|</td><td>gb|MN768471.1|</td><td>gb|MN765271.1|</td></tr>
<tr><td>gb|MN768560.1|</td><td>gb|MN768499.1|</td><td>gb|MN768323.1|</td><td>gb|OP882564.1|</td><td>gb|MN765305.1|</td></tr>
<tr><td>gb|MN768223.1|</td><td>gb|MN768389.1|</td><td>gb|MN768359.1|</td><td>gb|MN765219.1|</td><td>gb|MN765306.1|</td></tr>
<tr><td>gb|MN768220.1|</td><td>gb|MN768564.1|</td><td>gb|MN768403.1|</td><td>gb|MN765215.1|</td><td>gb|MN765282.1|</td></tr>
<tr><td>gb|MN768192.1|</td><td>gb|MN768440.1|</td><td>gb|MN768521.1|</td><td>gb|MN765205.1|</td><td>gb|MN765276.1|</td></tr>
<tr><td>gb|MN768547.1|</td><td>gb|MN768565.1|</td><td>gb|MN768448.1|</td><td>gb|MN765206.1|</td><td>gb|OL694837.1|</td></tr>
<tr><td>gb|MN768272.1|</td><td>gb|MN768346.1|</td><td>gb|MN768568.1|</td><td>gb|MN765200.1|</td><td>gb|MN765568.1|</td></tr>
<tr><td>gb|MN768276.1|</td><td>gb|MN768327.1|</td><td>gb|MN768401.1|</td><td>gb|MN765541.1|</td><td>gb|MN765657.1|</td></tr>
<tr><td>gb|MN768271.1|</td><td>gb|MN768413.1|</td><td>gb|MN768312.1|</td><td>gb|OL694840.1|</td><td>gb|MN765565.1|</td></tr>
<tr><td>gb|MN768268.1|</td><td>gb|MN768513.1|</td><td>gb|MN768470.1|</td><td>gb|MN765328.1|</td><td>gb|MN765581.1|</td></tr>
<tr><td>gb|MN768253.1|</td><td>gb|MN768383.1|</td><td>gb|MN768447.1|</td><td>gb|MN765527.1|</td><td>gb|MN765592.1|</td></tr>
<tr><td>gb|MN768281.1|</td><td>gb|MN768396.1|</td><td>gb|MN768380.1|</td><td>gb|MN765354.1|</td><td>gb|OP985293.1|</td></tr>
<tr><td>gb|MN768250.1|</td><td>gb|MN768525.1|</td><td>gb|MN768516.1|</td><td>gb|MN765503.1|</td><td>gb|OP985294.1|</td></tr>
<tr><td>gb|MN768241.1|</td><td>gb|MN768316.1|</td><td>gb|MN768519.1|</td><td>gb|MN765407.1|</td><td>gb|MN765654.1|</td></tr>
<tr><td>gb|MN768282.1|</td><td>gb|MN768382.1|</td><td>gb|MN768507.1|</td><td>gb|MN765418.1|</td><td>gb|MN765611.1|</td></tr>
<tr><td>gb|MN768248.1|</td><td>gb|MN768366.1|</td><td>gb|MN768490.1|</td><td>gb|MN765478.1|</td><td>gb|MN765636.1|</td></tr>
<tr><td>gb|MN768254.1|</td><td>gb|MN768420.1|</td><td>gb|MN768474.1|</td><td>gb|MN765515.1|</td><td>gb|MN765673.1|</td></tr>
<tr><td>gb|MN768236.1|</td><td>gb|MN768402.1|</td><td>gb|MN768311.1|</td><td>gb|MN765337.1|</td><td>gb|MN765549.1|</td></tr>
<tr><td>gb|MN768265.1|</td><td>gb|MN768400.1|</td><td>gb|MN768286.1|</td><td>gb|MN765484.1|</td><td>gb|MN765603.1|</td></tr>
<tr><td>gb|MN768245.1|</td><td>gb|MN768506.1|</td><td>gb|MN768415.1|</td><td>gb|MN765392.1|</td><td>gb|MN766102.1|</td></tr>
<tr><td>gb|MN768251.1|</td><td>gb|MN768469.1|</td><td>gb|MN768491.1|</td><td>gb|MN765457.1|</td><td>gb|MN766103.1|</td></tr>
<tr><td>gb|MN768267.1|</td><td>gb|MN768452.1|</td><td>gb|MN768343.1|</td><td>gb|MN765494.1|</td><td>gb|MN765854.1|</td></tr>
<tr><td>gb|MN768554.1|</td><td>gb|MN768497.1|</td><td>gb|MN768384.1|</td><td>gb|MN765421.1|</td><td>gb|MN765856.1|</td></tr>
<tr><td>gb|MN768546.1|</td><td>gb|MN768379.1|</td><td>gb|MN768390.1|</td><td>gb|MN765410.1|</td><td>gb|MN766061.1|</td></tr>
<tr><td>gb|MN768557.1|</td><td>gb|MN768510.1|</td><td>gb|MN768475.1|</td><td>gb|MN765444.1|</td><td>gb|MN766035.1|</td></tr>
<tr><td>gb|MN768556.1|</td><td>gb|MN768526.1|</td><td>gb|MN768456.1|</td><td>gb|MN765500.1|</td><td>gb|MN766030.1|</td></tr>
<tr><td>gb|MN768231.1|</td><td>gb|MN768562.1|</td><td>gb|MN768517.1|</td><td>gb|MN765517.1|</td><td>gb|MN765975.1|</td></tr>
<tr><td>gb|MN768561.1|</td><td>gb|MN768362.1|</td><td>gb|MN768352.1|</td><td>gb|MN765419.1|</td><td>gb|MN766025.1|</td></tr>
<tr><td>gb|MN768196.1|</td><td>gb|MN768567.1|</td><td>gb|MN768514.1|</td><td>gb|MN765495.1|</td><td>gb|MN766059.1|</td></tr>
<tr><td>gb|MN768187.1|</td><td>gb|MN768424.1|</td><td>gb|MN768345.1|</td><td>gb|MN765448.1|</td><td>gb|MN766094.1|</td></tr>
<tr><td>gb|MN768508.1|</td><td>gb|MN768310.1|</td><td>gb|MN768509.1|</td><td>gb|MN765456.1|</td><td>gb|MN766087.1|</td></tr>
<tr><td>gb|MN768476.1|</td><td>gb|MN768398.1|</td><td>gb|MN768426.1|</td><td>gb|MN765530.1|</td><td>gb|MN765984.1|</td></tr>
<tr><td>gb|MN768468.1|</td><td>gb|MN768528.1|</td><td>gb|MN768328.1|</td><td>gb|MN765522.1|</td><td>gb|MN765985.1|</td></tr>
</table>

gb|MN767611.1|
gb|MN767693.1|
gb|MN767640.1|
gb|MN767463.1|
gb|MN767450.1|
gb|MN767658.1|
gb|MN767628.1|
gb|MN767684.1|
gb|MN767637.1|
gb|MN767683.1|
gb|MN767644.1|
gb|MN767128.1|
gb|MN767135.1|
gb|MN767137.1|
gb|MN767688.1|
gb|MN767112.1|
gb|MN767117.1|
gb|MN767102.1|
gb|MN767682.1|
gb|MN767690.1|
gb|MN767666.1|
gb|MN767685.1|
gb|MN767539.1|
gb|MN767493.1|
gb|MN767500.1|
gb|MN767499.1|
gb|MN767540.1|
gb|MN767652.1|
gb|MN768036.1|
gb|MN767497.1|
gb|MN767532.1|
gb|MN767533.1|
gb|MN767537.1|
gb|MN767536.1|
gb|MN767205.1|
gb|MN767052.1|
gb|MN767223.1|
gb|MN767050.1|
gb|MN767054.1|
gb|MN768029.1|
gb|MN767075.1|
gb|MN767227.1|
gb|MN767056.1|
gb|MN767070.1|
gb|MN767024.1|
gb|MN767010.1|
gb|MN767226.1|
gb|MN768010.1|
gb|MN767225.1|

gb|MN767225.1|
gb|MN767088.1|
gb|MN767219.1|
gb|MN767231.1|
gb|MN767032.1|
gb|MN767055.1|
gb|MN767216.1|
gb|MN767222.1|
gb|MN767049.1|
gb|MN767027.1|
gb|MN767069.1|
gb|MN767083.1|
gb|MN767057.1|
gb|MN767228.1|
gb|MN767230.1|
gb|MN768049.1|
gb|MN768019.1|
gb|MN767946.1|
gb|MN767948.1|
gb|MN767995.1|
gb|MN768000.1|
gb|MN768001.1|
gb|MN767999.1|
gb|MN768002.1|
gb|MN767987.1|
gb|MN767827.1|
gb|MN767823.1|
gb|MN768003.1|
gb|MN767972.1|
gb|MN767967.1|
gb|MN768007.1|
gb|MN767759.1|
gb|MN767760.1|
gb|MN767958.1|
gb|MN767961.1|
gb|MN767828.1|
gb|MN767976.1|
gb|MN767977.1|
gb|KP343839.1|
gb|KP036971.1|
gb|KT163896.1|
gb|MN768066.1|
gb|MT783406.1|
gb|MN768092.1|
gb|KP343845.1|
gb|MN768129.1|
gb|MT501648.1|
gb|MT501645.1|
gb|MT501652.1|

gb|KT163879.1|
gb|MK820644.1|
gb|MN768140.1|
gb|MN768128.1|
gb|MN768062.1|
gb|DQ361268.1|
gb|MN768078.1|
gb|MN768117.1|
gb|MN768119.1|
gb|MN768114.1|
gb|MN768153.1|
gb|JN980171.1|
gb|MN768070.1|
gb|MN768138.1|
gb|MN768085.1|
gb|MN768084.1|
gb|MN768159.1|
gb|MN768116.1|
gb|MN768068.1|
gb|KT163875.1|
gb|MN768102.1|
gb|MN768135.1|
gb|MN768136.1|
gb|MN768148.1|
gb|MN768126.1|
gb|MN768090.1|
gb|AY823988.1|
gb|KT163903.1|
gb|MN768131.1|
gb|MT783405.1|
gb|MN768105.1|
gb|MN768059.1|
gb|MN768063.1|
gb|HM449773.1|
gb|MN768161.1|
gb|MN768067.1|
gb|KT163880.1|
gb|KT163886.1|
gb|KT163893.1|
gb|AY823989.1|
gb|KT163915.1|
gb|KJ194502.1|
gb|MN768077.1|
gb|MN768170.1|

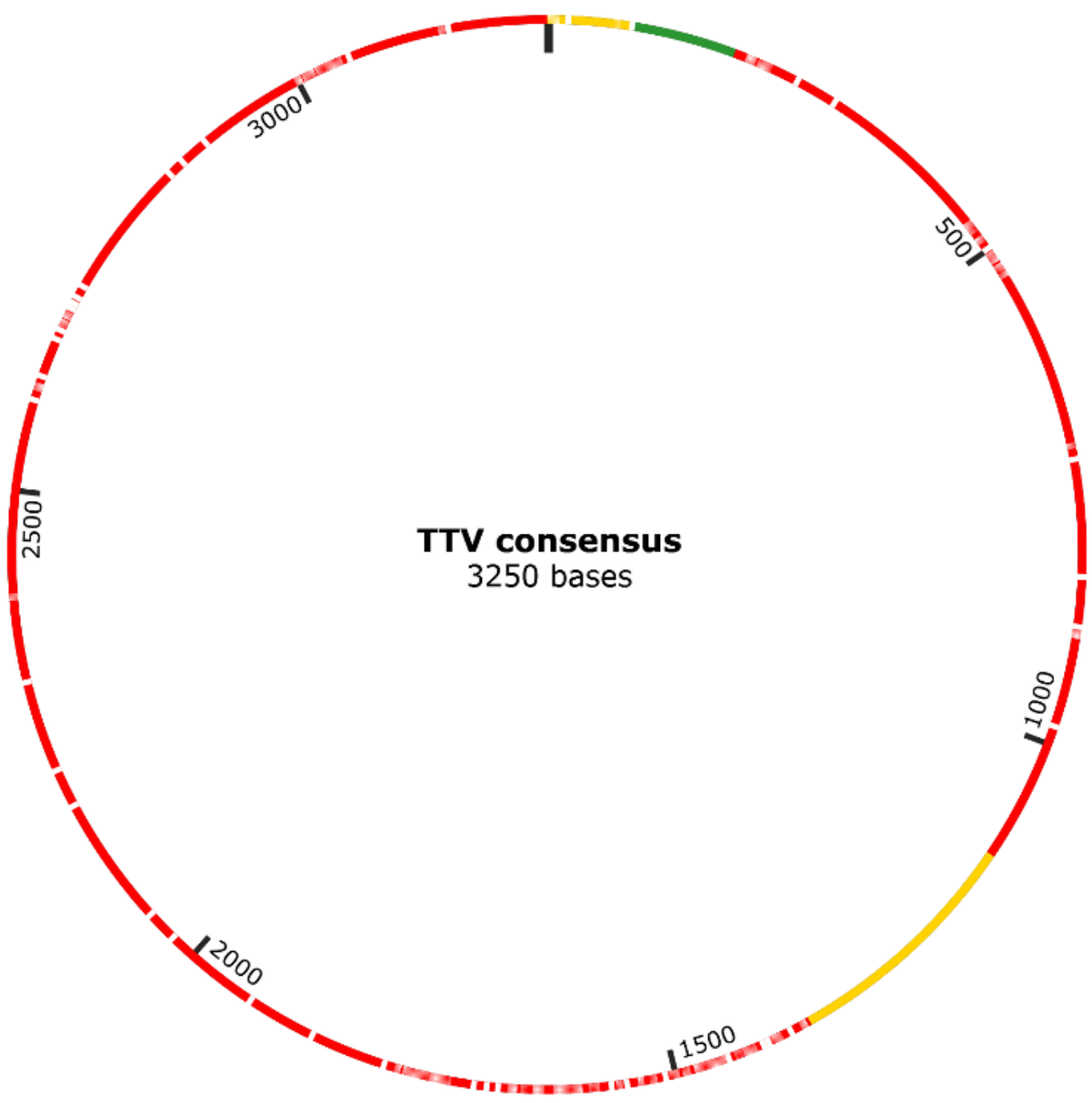

Appendix Figure S 4: Consensus sequence map of the TTV genome.
Showing a circular DNA structure with a length of 3250 bases. Key regions are marked in different colours to represent distinct functional segments within the genome, facilitating comparative analysis and annotation of conserved se-quences across TTV strains. Red: < 20 %, Yellow: < 50 %, Green: > 75 %, White: no alignment possible

A 5′ ATATAAGTAA**S**TGCACTTCCGAATGGCTGAGTTTTCCACGC
CCGTCCGCAGCG**RB**AACGCCAC**V**GAG**SRRNNNNY**C**SM**GCGTCCCG
WGGGCGGGTGCCG**R**AGGTGAGTTTACACACCG**V**AGTCAAGGGGCA
ATTCGGGCTCGGGACTGGCCGGGCTATGGGCAAGGCTCTTA 3′

B 5′ ATATAA**VBMRSN**GC**RS**T**KW**CGAATGG**YW**GAGTTTT**YYMY**GC
CCGTCCGC**R**GCG**RBRDMRMVVSVR**AG**SRRNNNNY**C**SM**GCGTCCCG
WGGGCGGGTGCCG**R**AGGTGAGTTTACACACCG**V**AGTCAAGGGGCA
ATTCGGGC**W**CGGGACTGGCCGGGCT**M**TGGGCAAG**R**CTCTTA 3′

C 5′ ATATAA**VBMRNNVBRS**T**KW**CGAATGG**YW**GAGTTT**WYYMY**GC
CCGTCCGC**R**GCG**RBRDMRMVVSVRMRSRRNNNNYHSM**GCG**W**CCCG
WGGGCGGG**W**GCCG**R**AGGTGAGTTTACACACCG**V**AGTC**W**AGGGGCA
ATTCGGGC**W**CGGG**W**CTGGCCGGGC**YMY**GGGCAAG**RY**TCT**K**A 3′

Appendix Figure S 5: Conserved sequence of 172 bp within the UTR of the TTV genome.
Demonstrating varying levels of sequence similarity: A: 75 % identity, B: 85 % identity, C: 95 % identity. Less conserved or degenerated nucleotides are indi-cated in bold. Remaining regions are of high conservation critical for detection of TTV with qPCR.

Appendix Table S 2: All TTV specific oligos used for PCR.

**Primer/Probe**		**Sequence (5’ → 3’)**	**Source/Design Tool/Manufacturer**
Maggi et al.	fwd*	GTGCCGIAGGTGAGTTTA	Original published: *Maggi et al.*, 2001<br>Manufacturer: IDT
	fwd m	GTGCCGIAGGTGAGTTTmA	
	rev	AGCCCGGCCAGTCC	
	rev m	AGCCCGGCCAGTmCC	
	rev 2m	AGCCCGGCCAGTmCmC	
	probe FAM	[FAM]TCAAGGGGCAATTCGGGCT[BHQ1]	
	probe HEX	[HEX]TCAAGGGGCAATTCGGGCT[BHQ2]	
TUM 1	fwd	TTACACACCGVAGTCAAGGG	Design Tool: TUM 1<br>Manufacturer: IDT
	fwd m	TTACACACCGVAGTCAAGmGG	
	fwd 2m	TTACACACCGVAGTCAAGmGmG	
	rev	TAAGAGCCTTGCCCATAGCC	
	rev m	TAAGAGCCTTGCCCATAGmCC	
	rev 2m	TAAGAGCCTTGCCCATAGmCmC	
	probe FAM	[FAM]CGGCCAGTCCCGAGCCCGAA[BHQ1]	
	probe HEX	[HEX] CGGCCAGTCCCGAGCCCGAA [BHQ2]	
TUM 2	fwd	AGTAASTGCACTTCCGAATGG	Design Tool: TUM 2<br>Manufacturer: IDT
	rev	TGTAAACTCACCTYCGGCAC	
	probe	[FAM]GAGTTTTCCACGCCCGTC[BHQ1]	
TUM 3	fwd	CACTTCCGAATGGCTGAGTTTTCCAC	Design Tool: TUM 3<br>Manufacturer: IDT
	rev	GAGCCCGAATTGCCCCTTGAC	
	probe	[FAM]GTGGCGTTVYCGCTGCGGACGGG[BHQ1]	

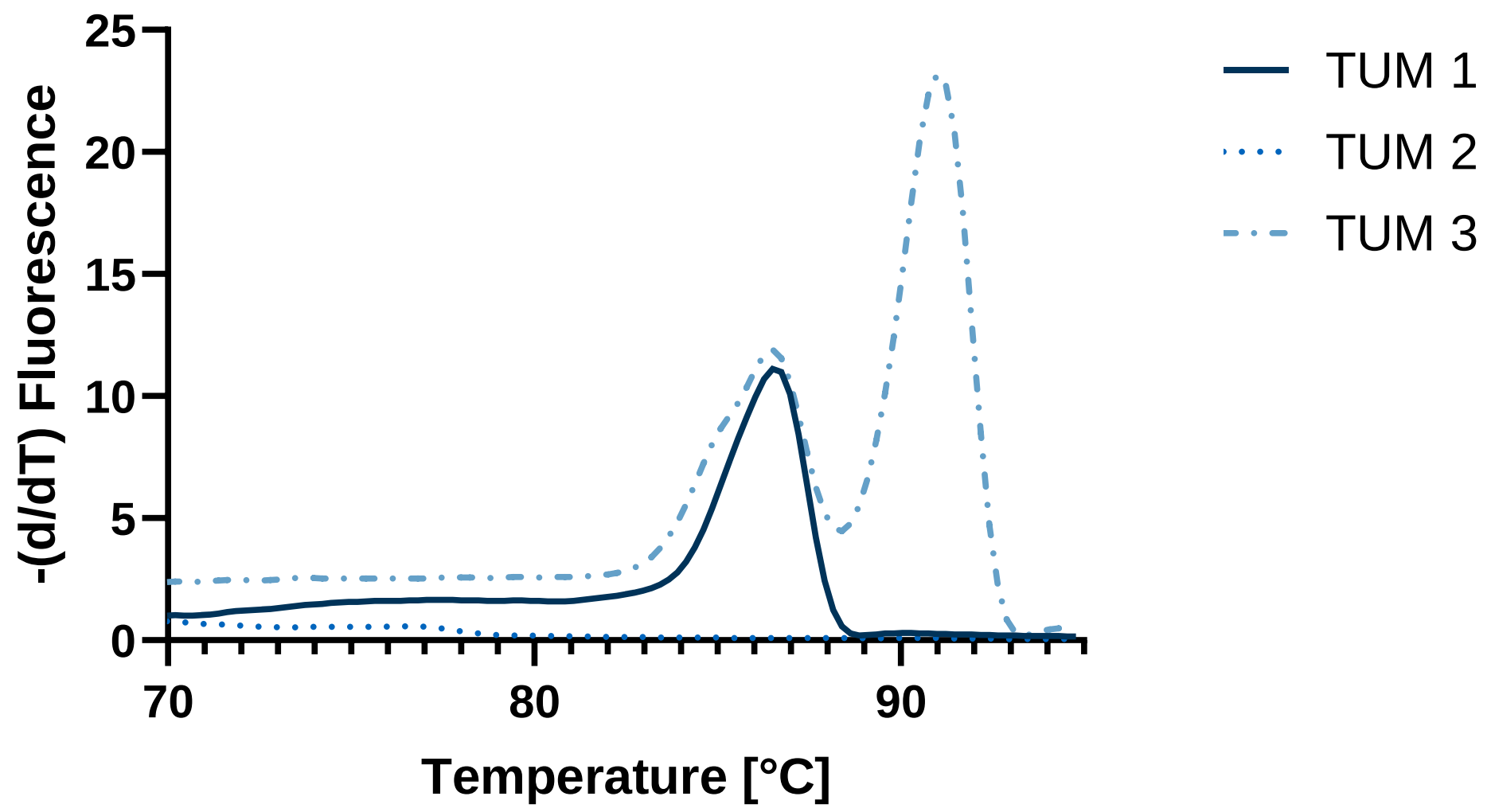

Appendix Figure S 6: HRM analysis comparing TTV-specific primers.
Melting profiles for all primer sets: TUM 1 (Blue), TUM 2 (light blue), and TUM 3 (Orange), showing distinct melting temperatures and peak shapes. Indicating varying levels of specificity and potential binding efficiency, with distinct melting peaks corresponding to each pri-mer set. TUM 3 primer showing peaks indicating unspecific amplification during PCR. TUM 1 primers showing just the specific TTV amplicon peak.

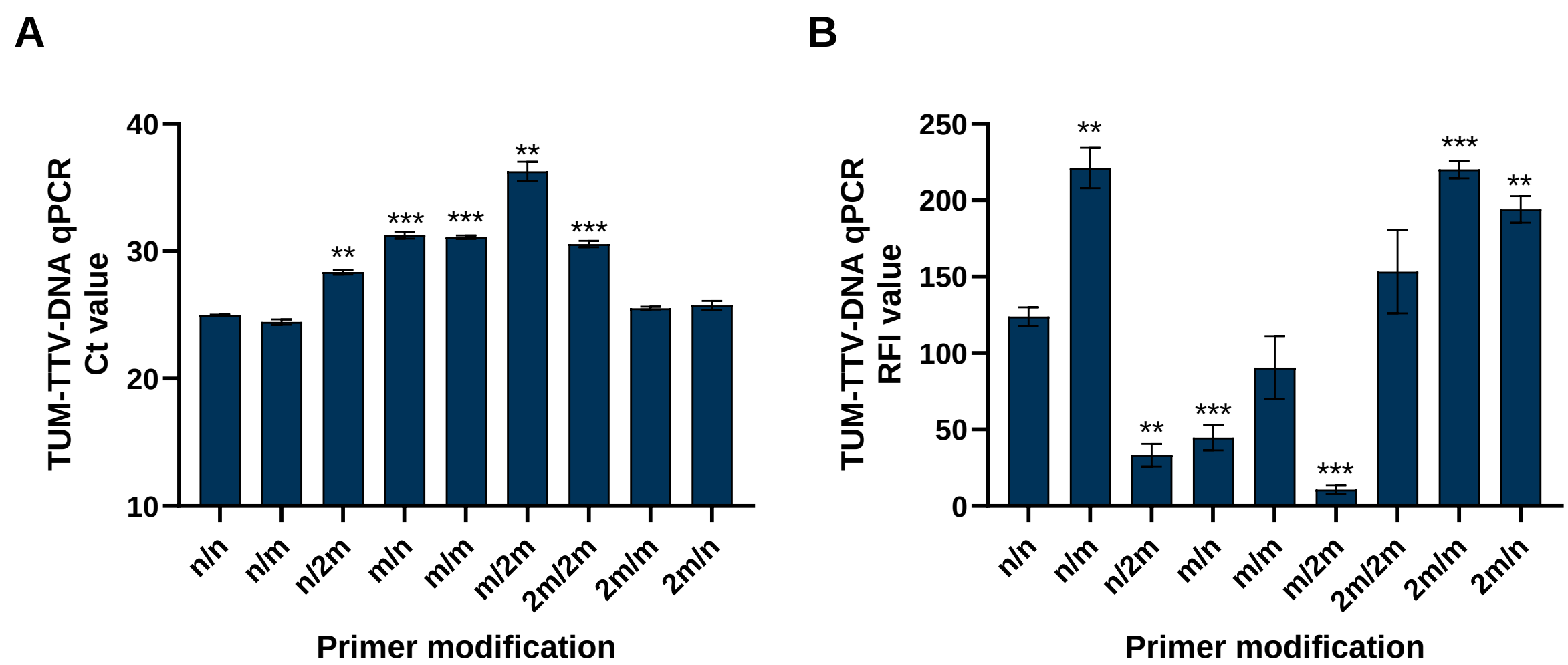

Appendix Figure S 7: Comparison of modified TUM 1 primers for TTV detection.
Forward and reverse primers (forward/reverse) of TUM 1 were 2' O-Methylated at the 3'-End. Methylation is either at the penultimate (m) or at the last and penultimate (2m) base. Unmodified primers are indicated as n. Comparison of Ct values (A) and RFI (B) of different combinations of modified primers. Data are shown as mean ± SD of triplicate measurements (n=3). Statistical significance was assessed using a paired t-test to the unmodified primer set (n/n). $P < 0.01$ is indicated by **, $P < 0.001$ by ***, and all comparisons not indicated were considered non-significant ($P > 0.05$). Exact p-values are described in Appendix Table S 4.

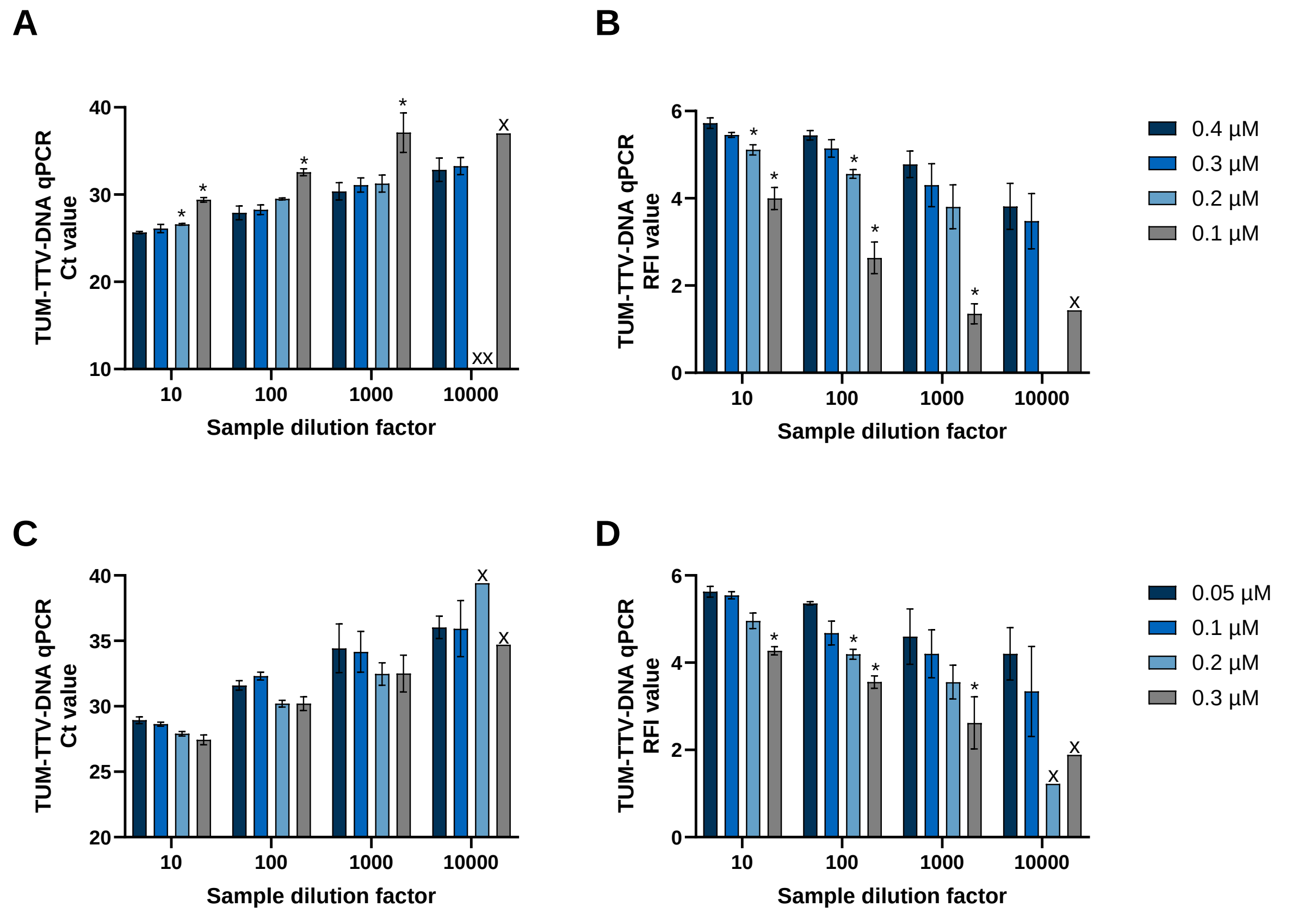

Appendix Figure S 8: Optimization of primer and probe concentration of the TUM 1 2m/m primers. Evaluation of Ct value (A, C) and RFI (B, D) four different primer concentrations (A, B): 0.4 µM, 0.3 µM, 0.2 µM and 0.1 µM and four different probe concentrations (C, D): 0.05 µM, 0.1 µM, 0.2 µM and 0.3 µM. Serial dilution of a TTV positive plasma sample was measured. Data are shown as mean ± SD of triplicate measurements (n=3). Statistical significance was assessed using a paired t-test to the selected concentrations (0.4 µM primer and 0.05 µM probe). P < 0.05 is indicated by *, and all comparisons not indicated were considered non-significant (P > 0.05). x indicates only one amplified replicate and xx indicates all replicates are negative. Exact p-values are described in Appendix Table S 4.

```
5'   ATATAAVBMRNNVBRSTKWCGAATGGYWGAGTTTWYYMYGCCCGTCCGCRGCGRBR

                                                    TTACACAC
DMRMVVSVRMRSRRNNNNYHSMGCGWCCCGWGGGCGGGWGCCGRAGGTGAGTTTACACAC

CGVAGTCAAGGG⇨
CGVAGTCWAGGGGCAATTCGGGCWCGGGWCTGGCCGGGCYMYGGGCAAGRYTCTKA   3'
             AAGCCCGAGCCCTGACCGGC
                                 ⇦CCGATACCCGTTCCGAGAAT
```

Appendix Figure S 9: TTV consensus sequence with 95 % sequence homology and bound TUM 1 primers/probe.
Forward and reverse primers were indicated with arrows (blue), probe was indi-cated in italic (blue). Sequence ambiguities in form of degenerated bases were indicated in bold. Nine mismatches of primer/probe and the consensus sequence were indicated in red.

```
TTV V:      5’ TTACACACCGVAGTCAAGGGGCAATTCGGGCTCGGGACTGGCCGGGCTATGGGCAAGGCTCTTA 3’
TTV A:      5’ TTACACACCGAAGTCAAGGGGCAATTCGGGCTCGGGACTGGCCGGGCTATGGGCAAGGCTCTTA 3’
TTV C:      5’ TTACACACCGCAGTCAAGGGGCAATTCGGGCTCGGGACTGGCCGGGCTATGGGCAAGGCTCTTA 3’
TTV G:      5’ TTACACACCGGAGTCAAGGGGCAATTCGGGCTCGGGACTGGCCGGGCTATGGGCAAGGCTCTTA 3’
Mutation 1: 5’ TTACACACCGCAGTCTAGGGGCAATTCGGGCACGGGTCTGGCCGGGCTATGGGCAAGGCTCTTA 3’
Mutation 2: 5’ TTACACACCGCAGTCAAGGGGCAATTCGGGCTCGGGACTGGCCGGGCTATGGGCAAGGCTCTGA 3’
Mutation 3: 5’ TTACACACCGCAGTCAAGGGGCAATTCGGGCACGGGACTGGCCGGGCTATGGGCAAGATTCTTA 3’
Mutation 4: 5’ TTACACACCGCAGTCAAGGGGCAATTCGGGCTCGGGACTGGCCGGGCTATGGGCAAGACTCTTA 3’
Mutation 5: 5’ TTACACACCGCAGTCAAGGGGCAATTCGGGCTCGGGACTGGCCGGGCCCCGGGCAAGGCTCTTA 3’
Mutation 6: 5’ TTACACACCGCAGTCAAGGGGCAATTCGGGCACGGGACTGGCCGGGCTATGGGCAAGGCTCTTA 3’
```

Appendix Figure S 10: Different constructs of the TTV target sequence of TUM 1 primer/probe. As a reference, TTV V represents the standard target sequence. TTV A, C, and G each represent the individual sequences contained within TTV V. The following six mutations represent the most common mutations, found in more than 10 % of TTV sequences. Forward and reverse primers are indicated in blue, and the probe is indicated in blue and italic. Deviations from the TTV consensus sequence are shown in red.

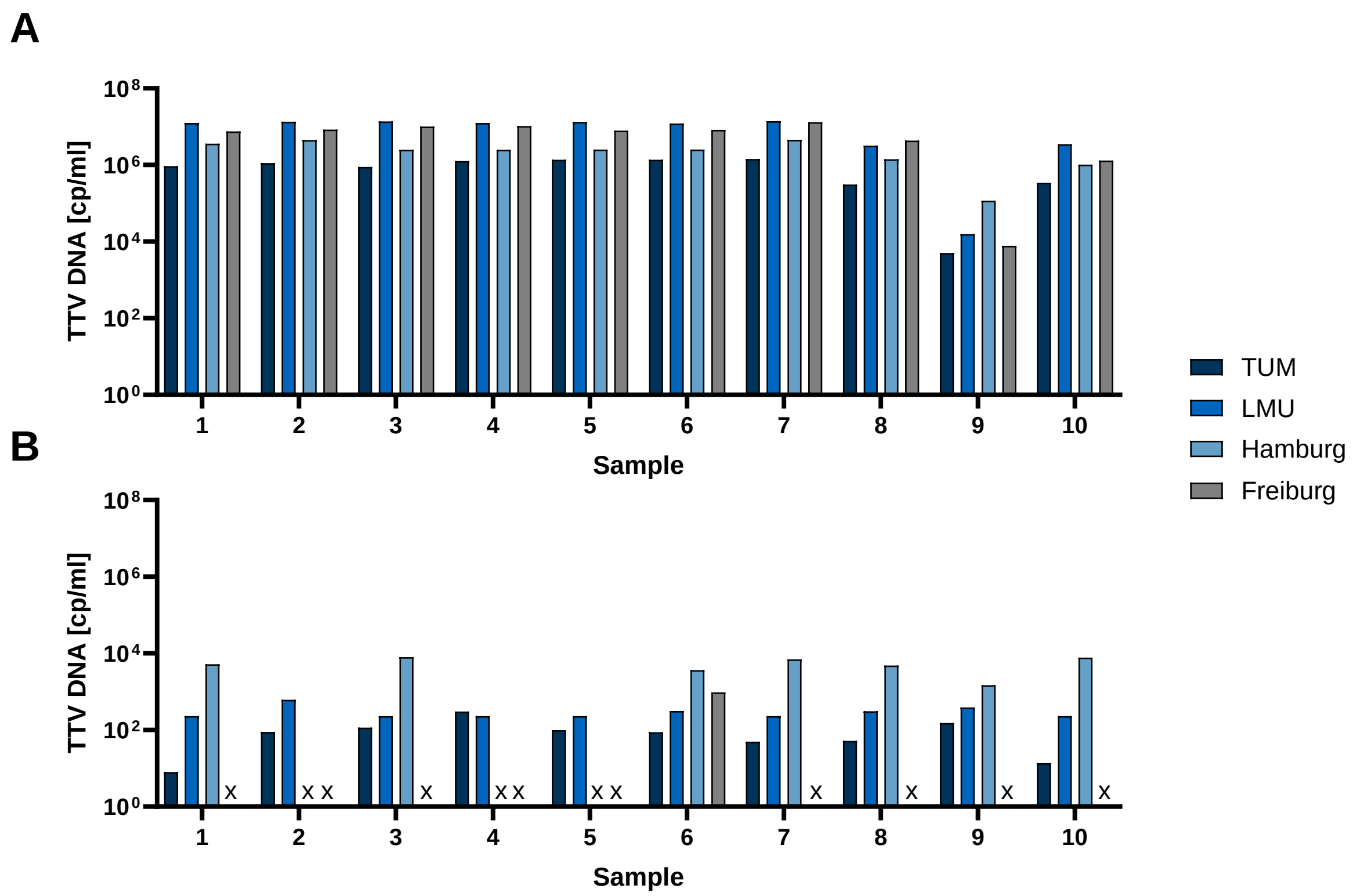

Appendix Figure S 11: Inter-laboratory comparison of TTV PCR assays.
Quantitative and qualitative performance of the TUM-TTV-PCR assay with three independent TTV PCR assays conducted in three different laboratories was assessed using a shared sample panel. Detection results were compared to evaluate assay sensitivity (absence of false-negative results) and specificity (absence of false-positive results in negative samples). Quantitative viral load measurements (copies/mL) were analyzed across the analytical measuring range of high positive (A) and low positive (B) samples to assess agreement between assays and laboratories. x indicates non-detected data points.

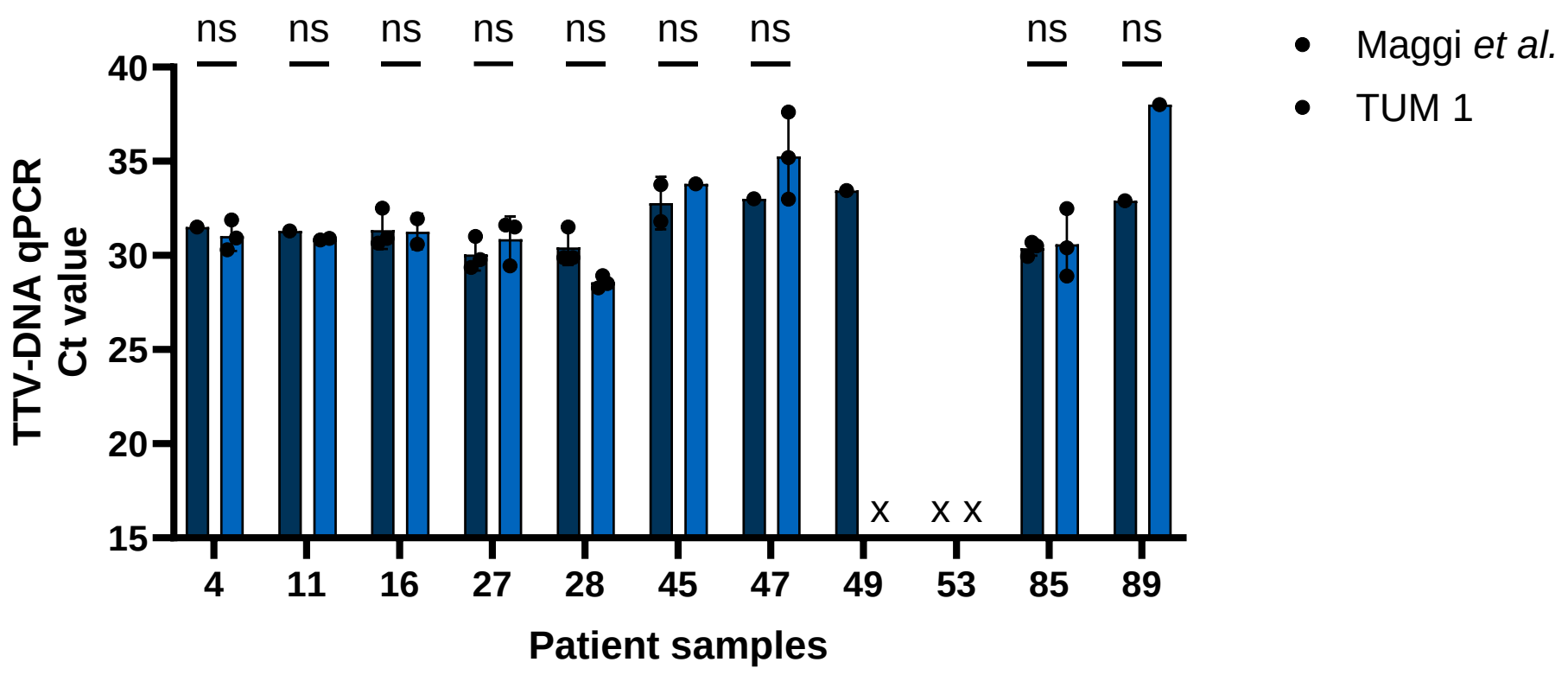

Appendix Figure S 12: Comparison of Ct values for selected samples.
Samples with high discrepancies in Ct values between two TTV primer sets (*Maggi et al.* and TUM 1) from figure 12. The measurement was performed in triplicate, and the mean and standard deviation were then calculated for each sample. Data are shown as individual measurements with mean ± SD of triplicate measurements (n=3). Statistical significance was assessed using a paired t-test. and all comparisons were considered non-significant (ns, P > 0.05). x indicates non-detected data point. Exact p-values are described in Appendix Table S 4.

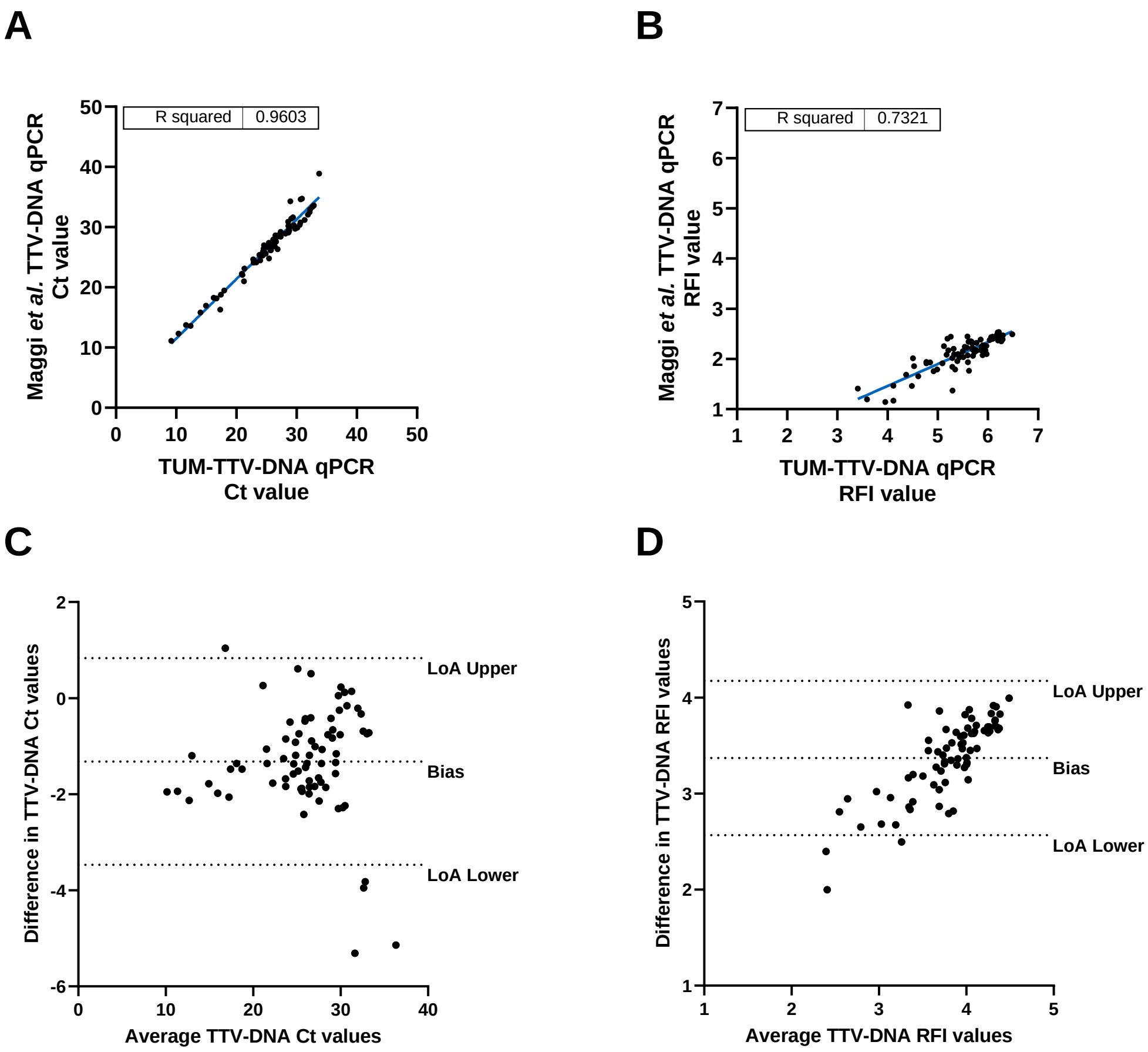

Appendix Figure S 13: Comparison of two TTV-DNA qPCR assays.
(A) Scatter plot of Ct values obtained with the TUM-TTV-DNA qPCR assay versus the *Maggi et al.* assay, including linear regression analysis with coefficient of determination (R^2). (B) Scatter plot of RFI values measured by both assays, also showing linear regression and corresponding R^2. (C) Bland–Altman plot of Ct values comparing the TUM-TTV-DNA qPCR assay to the *Maggi et al.* assay. (D) Bland–Altman plot of RFI values comparing the TUM assay to the *Maggi et al.* assay. In the Bland–Altman analyses, the mean bias and the upper and lower 95% limits of agreement are indicated.

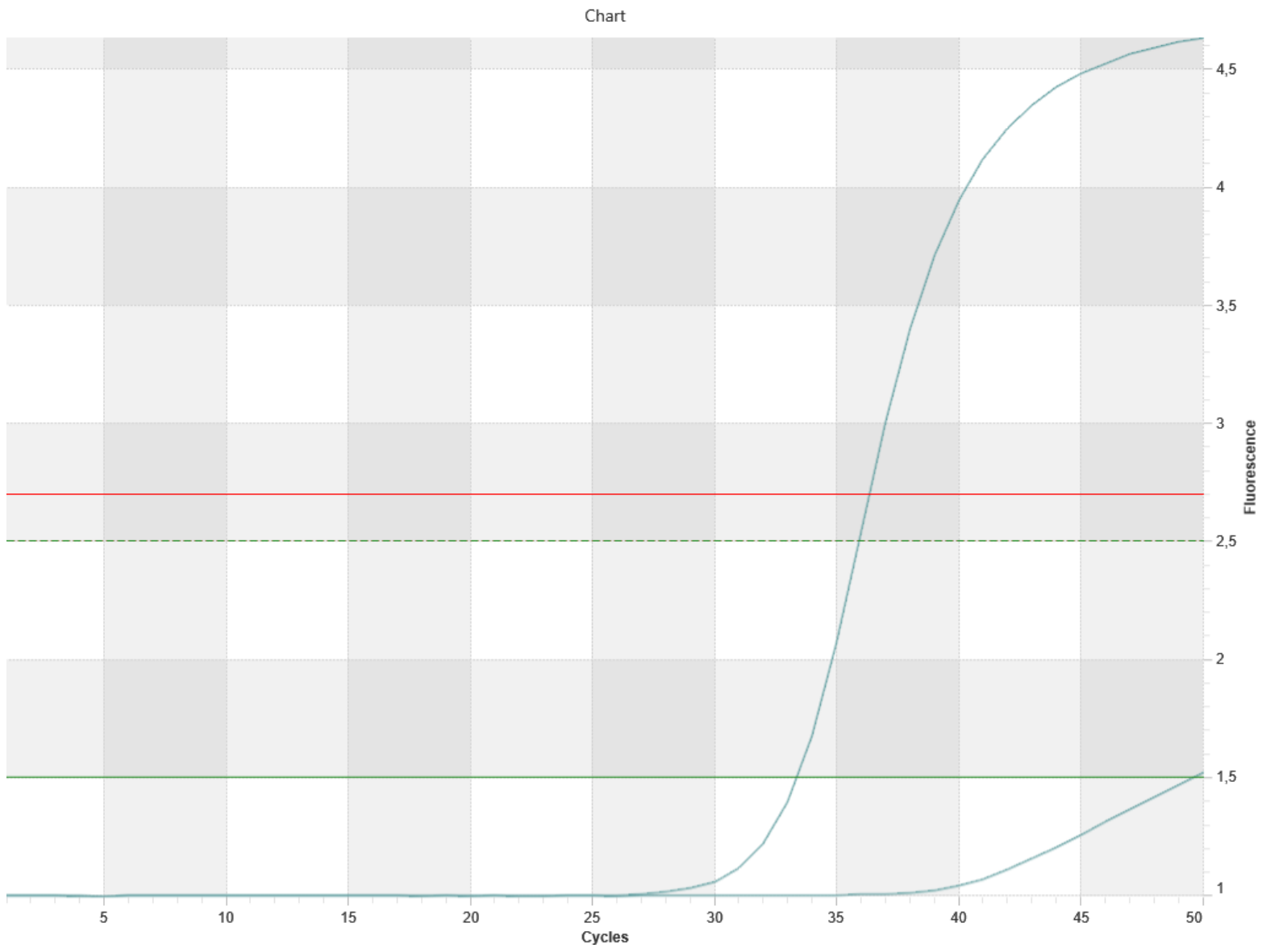

Appendix Figure S 14: Amplification curve of a false positive TTV sample.
Amplification of two plasma samples using the *Maggi et al.* assay on the cobas 5800 system. The first sample shows a typical qPCR amplification curve, consistent with a positive result. The second sample exhibits non-specific amplification unrelated to TTV. Lowering the RFI threshold from 2.7 (red) to 1.5 (green) results in a false-positive classification of this sample.

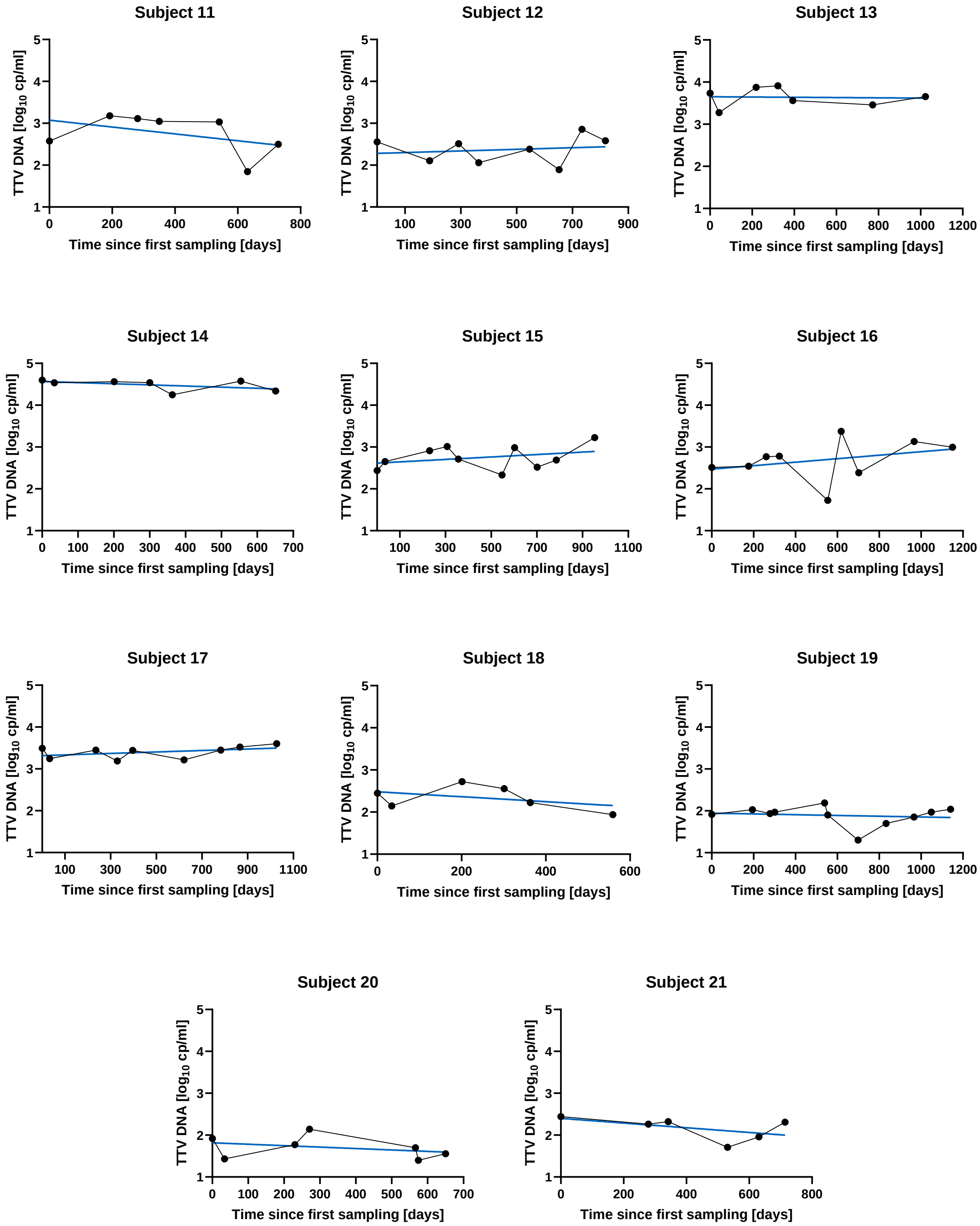

Appendix Figure S 15: Longitudinal TTV-DNA levels in patients without iatrogenic immunosuppression.

TTV-DNA concentrations were measured longitudinally in plasma samples from 11 patients without any iatrogenic immunosuppressive medication. Individual patient time courses are shown (black) to illustrate intra-individual fluctuations of TTV-DNA over 559 to 1150 days. Trend lines of TTV-DNA were generated using linear regression of the transformed data (blue).

Appendix Table S 3: Mathematical characterization of longitudinal variability in TTV-DNA loads in subjects (subject 11-21) with no iatrogenic-immunosuppression.

Name and observation period	Mean TTV-DNA (Min.-Max.) [cp/ml]	Dynamic range Min. to Max. [cp/ml]	Fold change Max/Min	SD	Coefficient of variation	Rate of change [cp/ml per day]	Longitudinal slope
Subject 11 729 days	8.20E+02 (6.98E+01 - 1.50E+03)	1.43E+03	21.5 (1.3 $\log_{10}$)	555.9121	0.52	1.97E+00	-0.000819
Subject 12 818 days	2.93E+02 (7.80E+01 - 7.15E+02)	6.37E+02	9.2 (1 $\log_{10}$)	207.0229	0.73	7.79E-01	0.000194
Subject 13 1023 days	4.86E+03 (1.89E+03 - 8.18E+03)	6.29E+03	4.3 (0.6 $\log_{10}$)	2332.976	0.51	6.15E+00	-3.23E-05
Subject 14 651 days	3.17E+04 (1.77E+04 - 3.96E+04)	2.19E+04	2.2 (0.3 $\log_{10}$)	8419.402	0.24	3.36E+01	-0.000272
Subject 15 953 days	6.76E+02 (2.15E+02 - 1.68E+03)	1.46E+03	7.8 (0.9 $\log_{10}$)	451.8548	0.90	1.54E+00	0.000293
Subject 16 1150 days	7.63E+02 (5.30E+01 - 2.37E+03)	2.31E+03	44.7 (1.6 $\log_{10}$)	721.5907	1.23	2.01E+00	0.000417
Subject 17 1027 days	2.64E+03 (1.54E+03 - 4.01E+03)	2.47E+03	2.6 (0.4 $\log_{10}$)	838.6441	0.30	2.41E+00	0.000175
Subject 18 559 days	2.60E+02 (8.66E+01 - 5.26E+02)	4.39E+02	6.1 (0.8 $\log_{10}$)	163.7948	0.73	7.86E-01	-0.000587
Subject 19 1141 days	8.59E+01 (2.05E+01 - 1.54E+02)	1.34E+02	7.5 (0.9 $\log_{10}$)	33.92133	0.40	1.17E-01	-9.14E-05
Subject 20 650 days	5.98E+01 (2.49E+01 - 1.39E+02)	1.14E+02	5.6 (0.7 $\log_{10}$)	40.30408	0.80	1.75E-01	-0.000341
Subject 21 714 days	1.69E+02 (5.11E+01 - 2.76E+02)	2.25E+02	5.4 (0.7 $\log_{10}$)	83.05023	0.43	3.15E-01	-0.000562

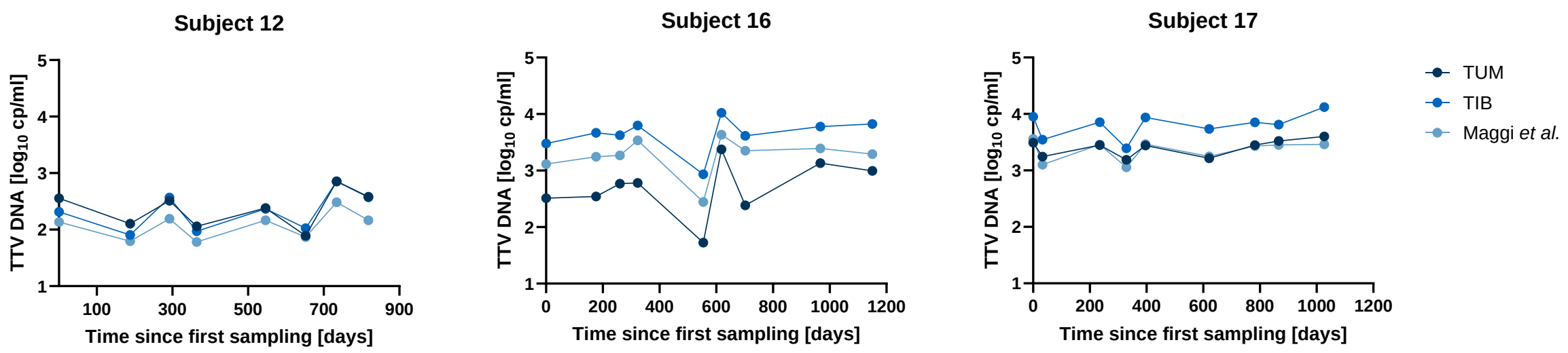

Appendix Figure S 16: Comparison of longitudinal TTV-DNA levels.
TTV-DNA concentrations were measured longitudinally in plasma samples from three patients with three different TTV-DNA qPCR assays (TUM (dark blue). TIB Molbiol (blue). *Maggi et al.* (light blue)) on the cobas 5800 system. Individual patient time courses are shown for each assay to illustrate intra-individual fluctuations of TTV-DNA over 559 to 1150 days.

Appendix Table S 4: Exact p-values obtained from all paired t-tests performed for the statistical comparisons presented in the figures.

Figure	Analysis	p-Value
1 D	Serum vs. Plasma	0,5410 (ns)
	Swab vs. Serum	0,5273 (ns)
	Swab vs. Plasma	0,5448 (ns)
2 D	Serum vs. Plasma	0,2172 (ns)
	Swab vs. Serum	0,7400 (ns)
	Swab vs. Plasma	0,5199 (ns)
EV 1 A	n/m vs. n/2m	0,0332 (*)
EV 1 B	n/2m vs. n/n	0,0262 (*)
	n/n vs. m/m	0,0010 (***)
	m/n vs. m/2m	0,0053 (**)
	n/n vs. m/2m	0,0398 (*)
	m/m vs. n/2m	0,0293 (*)
EV 1 C	n/m vs. m/m	0,0014 (**)
	n/n vs. m/m	0,0264 (*)
EV 1 D	m/m vs. m/n	0,0028 (**)
	m/n vs. n/m	0,0308 (*)
	n/n vs. n/m	0,0359 (*)
EV 1 E	n/m vs. m/m	0,0140 (*)
	m/n vs. m/2m	0,0335 (*)
EV 1 F	m/m vs. m/n	0,0039 (**)
	n/n vs. m/m	0,0236 (*)
	m/n vs. m/2m	0,0023 (**)
	n/n vs. m/2m	0,0099 (**)
	n/m vs. m/n	0,0185 (*)
	n/2m vs. n/m	0,0306 (*)
	n/m vs. n/n	0,0435 (*)
EV 2 B	TUM 1 vs. TUM 2	<0,0001 (****)
	TUM 1 vs. TUM 3	<0,0001 (****)
EV 2 C	TUM 1 vs. TUM 2	<0,0001 (****)
	TUM 1 vs. TUM 3	<0,0001 (****)
EV 3 B	2m/n vs. 2m/m	0,0191 (*)
	n/n vs. 2m/n	0,0350 (*)
	2m/m vs. n/n	0,0311 (*)
EV 3 C	2m/n vs. 2m/m	0,0489 (*)
EV 3 D	2m/n vs. 2m/m	0,0049 (**)
	n/n vs. 2m/m	0,0148 (*)
	2m/n vs. n/n	0,0244 (*)
EV 3 F	2m/m vs. n/n	0,0299 (*)
	2m/m vs. 2m/n	0,0110 (*)
	2m/m vs. n/m	0,0428 (*)
EV 4 B	Mutation 1 vs. Mutation 2	0,0031 (**)
	Mutation 1 vs. Mutation 4	0,0136 (*)
	Mutation 1 vs. Mutation 6	0,0130 (*)
	Mutation 2 vs. Mutation 3	0,0021 (**)
	Mutation 2 vs. Mutation 5	<0,0001 (****)
	Mutation 3 vs. Mutation 4	0,0096 (**)
	Mutation 3 vs. Mutation 5	0,0047 (**)
	Mutation 3 vs. Mutation 6	0,0045 (**)
	Mutation 4 vs. Mutation 5	0,0024 (**)
	Mutation 5 vs. Mutation 6	0,0031 (**)
S 1	1	0,819072 (ns)
	2	0,692398 (ns)
	3	0,086092 (ns)
	4	0,057975 (ns)
S 3 B	Dilution factor 10, 0.1 µM vs 0.4 µM	0,0447 (*)

S 3 C	Dilution factor 10, 0.05 µM vs 0.3 µM	0.0436 (*)
S 3 D	Dilution factor 10, 0.05 µM vs 0.3 µM	0.02572 (*)
	Dilution factor 100, 0.05 µM vs 0.3 µM	0.04801 (*)
S 7 A	n/n vs. n/2m	0,0010 (**)
	n/n vs. m/n	0,0007 (***)
	n/n vs. m/m	0,0002 (***)
	n/n vs. m/2m	0,0015 (**)
	n/n vs. 2m/2m	0,0008 (***)
S 7 B	n/n vs. n/m	0,0019 (**)
	n/n vs. n/2m	0,0030 (**)
	n/n vs. m/n	0,0005 (***)
	n/n vs. m/2m	0,0003 (***)
	n/n vs. 2m/m	0,0001 (***)
	n/n vs. 2m/n	0,0058 (**)
S 8 A	Dilution factor 10, 0.1 µM	0.0455 (*)
	Dilution factor 10, 0.2 µM	0.02187 (*)
	Dilution factor 100, 0.1 µM	0.0264 (*)
	Dilution factor 1000, 0.1 µM	0.0463 (*)
S 8 B	Dilution factor 10, 0.1 µM	0.0247 (*)
	Dilution factor 10, 0.2 µM	0.0463 (*)
	Dilution factor 100, 0.1 µM	0.0343 (*)
	Dilution factor 100, 0.2 µM	0.0161 (*)
	Dilution factor 1000, 0.1 µM	0.0459 (*)
S 8 D	Dilution factor 10, 0.3 µM	0.0459 (*)
	Dilution factor 100, 0.2 µM	0.0138 (*)
	Dilution factor 100, 0.3 µM	0.012 (*)
	Dilution factor 1000, 0.3 µM	0,0161 (*)
S 12	Sample 4	0.666252 (ns)
	Sample 11	0.099425 (ns)
	Sample 16	0.931923 (ns)
	Sample 27	0.399068 (ns)
	Sample 28	0.051973 (ns)
	Sample 45	0.658335 (ns)
	Sample 47	0.485065 (ns)
	Sample 85	0.848176 (ns)
	Sample 89	0.059625 (ns)
