# Supplementary material for: A novel Torque teno virus qPCR assay reveals longitudinal viral load variability among non-immunosuppressed subjects
Source: EMBO Mol Med. 2026 Oct 8;18(11):4618–42. doi: 10.1038/s44321-026-00540-3 (PMC13748663; doi:10.1038/s44321-026-00540-3)
Supplement: Supplementary file 2 [file 44321_2026_540_MOESM2_ESM.pdf]

EMBO Molecular Medicine

A novel Torque teno virus qPCR assay reveals longitudinal viral load variability among non-immunosuppressed subjects

Lara Valder, Thomas Grigore, Christian Schaaf, Quirin Bachmann, Lutz Renders, Samuel Jeske, Marc Lütgehetmann, Sibylle Bierbaum, Hartmut Hengel, Christopher Dächert, Ulrike Protzer, and Jochen Wettengel

Corresponding author(s): Jochen Wettengel (wettengel@tum.de)

Review Timeline:

Submission Date:	25th Mar 26
Editorial Decision:	24th Apr 26
Revision Received:	27th Jul 26
Editorial Decision:	13th Aug 26
Revision Received:	23rd Sep 26
Accepted:	25th Sep 26

Editor: Zeljko Durdevic

Transaction Report:

(Note: With the exception of the correction of typographical or spelling errors that could be a source of ambiguity, letters and reports are not edited. Depending on transfer agreements, referee reports obtained elsewhere may or may not be included in this compilation. Referee reports are anonymous unless the Referee chooses to sign their reports.)

24th Apr 2026

Dear Dr. Wettengel,

Thank you for the submission of your manuscript to EMBO Molecular Medicine. We have now received feedback from the three reviewers who agreed to evaluate your manuscript. All referees recognize interest of the study but also raise serious concerns that should be addressed in a major revision. If you would like to discuss further the points raised by the referees, I am available to do so via email or video. Let me know if you are interested in this option.

Further consideration of a revision that addresses reviewers' concerns in full will entail a second round of review. EMBO Molecular Medicine encourages a single round of revision only and therefore, acceptance or rejection of the manuscript will depend on the completeness of your responses included in the next, final version of the manuscript. For this reason, and to save you from any frustrations in the end, I would strongly advise against returning an incomplete revision.

We would welcome the submission of a revised version within three months for further consideration. Please let us know if you require longer to complete the revision.

I look forward to receiving your revised manuscript.

Yours sincerely,

Zeljko Durdevic

Zeljko Durdevic
Editor
EMBO Molecular Medicine

*****
When preparing your revised manuscript, please refer to our guidelines: https://link.springer.com/journal/44321/submission-guidelines#cms-Revised-submissions. We perform an initial quality control of all revised manuscripts before re-review; failure to include requested items will delay the evaluation of your revision.

We require:

1) A .docx formatted version of the manuscript text (including legends for main figures, EV figures and tables). Please make sure that the changes are highlighted to be clearly visible.

2) Individual production quality figure files as .eps, .tif, .jpg (one file per figure). For guidance, download the 'Figure Guide PDF': https://media.springernature.com/original/springer-cms/rest/v1/content/27825798/data/v1.

3) A .docx formatted letter INCLUDING the reviewers' reports and your detailed point-by-point responses to their comments. As part of the EMBO Press transparent editorial process, the point-by-point response is part of the Review Process File (RPF), which will be published alongside your paper.

4) A complete author checklist, which you can download from our author guidelines. Please insert information in the checklist that is also reflected in the manuscript. The completed author checklist will also be part of the RPF.

5) Please note that all corresponding authors are required to supply an ORCID ID for their name upon submission of a revised manuscript.

6) It is mandatory to include a 'Data Availability' section after the Materials and Methods. Before submitting your revision, primary datasets produced in this study need to be deposited in an appropriate public database, and the accession numbers and database listed under 'Data Availability'. Please remember to provide a reviewer password if the datasets are not yet public.

In case you have no data that requires deposition in a public database, please state so in this section. Note that the Data Availability Section is restricted to new primary data that are part of this study.

7) For data quantification: please specify the name of the statistical test used to generate error bars and P values, the number (n) of independent experiments (specify technical or biological replicates) underlying each data point and the test used to calculate p-values in each figure legend. The figure legends should contain a basic description of n, P and the test applied. Graphs must include a description of the bars and the error bars (s.d., s.e.m.).

8) At EMBO Press we ask authors to provide source data for the main manuscript figures. You will receive a separate email with instructions for providing source data with your revised manuscript, including how to upload and organize the files.

9) Our journal encourages inclusion of *data citations in the reference list* to directly cite datasets that were re-used and obtained from public databases. Data citations in the article text are distinct from normal bibliographical citations and should directly link to the database records from which the data can be accessed. In the main text, data citations are formatted as follows: "Data ref: Smith et al, 2001" or "Data ref: NCBI Sequence Read Archive PRJNA342805, 2017". In the Reference list, data citations must be labeled with "[DATASET]". A data reference must provide the database name, accession number/identifiers and a resolvable link to the landing page from which the data can be accessed at the end of the reference.

10) We replaced Supplementary Information with Expanded View (EV) Figures and Tables that are collapsible/expandable online. A maximum of 5 EV Figures can be typeset. EV Figures should be cited as 'Figure EV1, Figure EV2" etc... in the text and their respective legends should be included in the main text after the legends of regular figures.

- For the figures that you do NOT wish to display as Expanded View figures, they should be bundled together with their legends in a single PDF file called *Appendix*, which should start with a short Table of Content. Appendix figures should be referred to in the main text as: "Appendix Figure S1, Appendix Figure S2" etc.

- Additional Tables/Datasets should be labeled and referred to as Table EV1, Dataset EV1, etc. Legends have to be provided in a separate tab in case of .xls files. Alternatively, the legend can be supplied as a separate text file (README) and zipped together with the Table/Dataset file.

11) The paper explained: EMBO Molecular Medicine articles are accompanied by a summary of the articles to emphasize the major findings in the paper and their medical implications for the non-specialist reader. Please provide a draft summary of your article highlighting

- the medical issue you are addressing,

- the results obtained and

- their clinical impact.

This may be edited to ensure that readers understand the significance and context of the research. Please refer to any of our published articles for an example.

12) Author contributions: You will be asked to provide CRediT (Contributor Role Taxonomy) terms in the submission system. These replace a narrative author contribution section in the manuscript.

13) A Conflict of Interest statement should be provided in the main text.

14) Every published paper includes a 'Synopsis' to further enhance discoverability. Synopses are displayed on the journal webpage and are freely accessible to all readers. They include a short stand first (maximum of 300 characters, including space) as well as 2-5 one-sentences bullet points that summarizes the paper. Please write the bullet points to summarize the key NEW findings. They should be designed to be complementary to the abstract - i.e. not repeat the same text. We encourage inclusion of key acronyms and quantitative information (maximum of 30 words / bullet point). Please use the passive voice. Please attach these in a separate file or send them by email, we will incorporate them accordingly.

Please also suggest a striking image or visual abstract to illustrate your article as a PNG file 550 px wide x 300-600 px high.

15) Include a Reagents and Tools Table as part of the Methods section, which can be downloaded from our author guidelines.

EMBO Molecular Medicine has a "scooping protection" policy, whereby similar findings that are published by others during review or revision are not a criterion for rejection. Should you decide to submit a revised version, I do ask that you get in touch after three months if you have not completed it, to update us on the status.

***** Reviewer's comments *****

Referee #1 (Comments on Novelty/Model System for Author):

While the assay development is technically sound and potentially useful for standardization, the limited cohort size and lack of immunological context restrict the biological and translational insight, preventing the study from reaching the level expected for EMBO Molecular Medicine.

Referee #1 (Remarks for Author):

Lara Valder and collegues report the development of a very robost and interesting high-throughput qPCR assay for TTV detection and apply it to longitudinal monitoring in non-immunosuppressed individuals. The assay is technically solid and represents a useful step toward standardization. However, the biological conclusions are derived from a very limited dataset and lack mechanistic support. As a result, the manuscript remains largely descriptive and does not provide sufficient conceptual or translational advance for EMBO Molecular Medicine.

Major Comments
1. Biological conclusions are not supported by the dataset
The manuscript states:
"Longitudinal analysis... revealed pronounced inter- and intra-individual variability" and "pronounced longitudinal fluctuations in TTV-DNA levels in patients without iatrogenic immunosuppression"
These statements are central to the manuscript but rely on o.nly 10 individuals (Fig. 4). The variability observed-while interesting-cannot be generalized and may reflect sampling noise or uncontrolled confounders. The study therefore supports an observation, not a biological principle.

2. Absence of immunological characterization precludes interpretation
The authors explicitly acknowledge:
"it cannot be conclusively determined whether the observed changes reflect modulation of immune function... or... intrinsic variability"
This is a critical limitation. Without immune profiling or clinical context, the study cannot distinguish between: immune-driven modulation, intercurrent infections and other factors such as intrinsic viral dynamics. As a consequence, the manuscript does not provide mechanistic insight into TTV biology or its use as a biomarker.

3. Overstated implications for TTV as biomarker
The manuscript repeatedly suggests clinical relevance: "important implications for the interpretation of TTV-DNA as a biomarker".
However, no direct link to immune status is demonstrated. In fact, the data undermine interpretability without offering a solution. This creates a disconnect between data and claims.

4. Insufficient consideration of anellovirus complexity
The assay targets a conserved UTR region and is claimed to cover:
"more than 95% of the evaluated TTV sequence variants"
However:
This is based on consensus/in silico design and limited synthetic constructs
No validation is performed on real multi-genotype samples
Given the complexity of the human "anellome," the assay likely captures only part of the viral landscape. This limitation directly affects interpretation of "TTV load" as a biological variable.

5. Lack of essential clinical metadata
The cohort is described as:
"patients without relevant comorbidities"
but no supporting data are provided (age, sex, clinical background, infections, treatments). This severely limits interpretability and reproducibility.

6. Conceptual imbalance: strong methods, weak biology
The assay validation is rigorous (Fig. 1-3), including: precision, linearity, inter-laboratory comparison. However, the biological component (Fig. 4) is: underpowered,
uncontrolled and purely descriptive. This imbalance prevents the manuscript from reaching the level of conceptual advance expected for this journal.

Minor Comments
The statement that TTV is "relatively stable over time" in prior studies is not quantitatively reconciled with the present findings.

The discussion of RFI thresholds (Sections 2.2 vs 2.4) would benefit from clearer rationale.
The claim of "no relationship" between viral load and variability should be supported by statistical analysis.
It would be nice if the authors trued to analyze also full blood rather than plasma.

Suggested Experiments / Analyses to substantially strengthen the manuscript:

Correlate TTV load with immune parameters (even retrospective data would help)
Screen for co-infections (e.g., CMV, EBV) to contextualize fluctuations
Assess genotype composition (NGS or genotype-resolved assays)
Define technical vs biological variability (short-interval replicates)
Validate findings in an independent cohort

Conclusion
This is a well-executed assay development study with clear technical merit. However, the biological component is underdeveloped and does not provide sufficient mechanistic or translational insight. Significant expansion of the cohort and integration of immunological context would be required to support the proposed conclusion and increase the utility of this study.

Referee #2 (Comments on Novelty/Model System for Author):

The work is well executed and analytically robust, with clear added value in standardising and automating TTV quantification. There are minor methodological limitations that can be easily addressed. Deployment of the assay to evaluate viral concentrations in longitudinal plasma samples from patients confirmed previous observations and did not, in itself, introduce any novelties.

Referee #2 (Remarks for Author):

This study describes the development and analytical validation of a high-throughput qPCR assay for Torque teno virus (TTV) implemented on fully automated platforms (Roche Cobas x800). The work is well conducted and methodologically solid, demonstrating robust analytical performance, including good precision, linearity, sensitivity, and lack of matrix effects. The newly developed assay outperforms Maggi's reference TTV assay in terms of signal intensity and low-level detection. Importantly and as previously demonstrated by other groups in independent studies, longitudinal analysis in immunocompetent individuals reveals substantial intra- and inter-individual variability in TTV viral load, highlighting limitations in the interpretation of TTV as a biomarker of immune status.
Overall, the study is interesting and relevant, particularly because it validates a TTV qPCR assay on automated systems such as Cobas supporting its potential use in routine diagnostics and large-scale studies.

Major issues
• The main concern relates to the generation of the consensus sequence used for primer design. The authors state that sequences were retrieved from NCBI Virus and aligned (MAFFT), but do not sufficiently describe:
o the query used to retrieve sequences,
o how they ensured that sequences were truly TTV (and not TTMV/TTMDV, given known annotation ambiguities in Anelloviridae databases),
o whether sequences were filtered (e.g., completeness, redundancy, duplicates),
o whether MAFFT alignment was performed on full genomes or restricted regions (e.g., UTR).
These methodological gaps limit reproducibility and should be clarified.
• Page 6, Section 2.4, second paragraph. Please state the TTV DNA concentrations used for inter- and intra-assay precision
• Page 7, Section 2.6. The sentence describing plasma stability should be modified to clearly state that nine days was the observed period, as the data explicitly show stability "for up to nine days" under different storage conditions.

Minor issues
• Several minor writing and punctuation errors are present throughout the manuscript and should be corrected, e.g. Magi that should be replaced with Maggi in Table 2, Appendix-Suppl. File.

Referee #3 (Comments on Novelty/Model System for Author):

While stated in the text that there are statistically significant differences/no differences the statistics is not always given.
Bland and altman analysis in Fig 3A might be better for comparison.
While it is highly interesting to see the different TTV courses in the 10 subjects there are no further investigations (seq info) to give some potential explanations.

Referee #3 (Remarks for Author):

The study with the title „A high-throughput qPCR for Torque teno virus reveals pronounced longitudinal viral load variability" submitted by Lara Valder and collegues established a high-throughput TTV qPCR assay in comparison to the Maggi et al., TTV assay and tested sensitivity, specificity and applicability on a set of TTV positive samples. While both assays are comparable, the newly established TUM TTV assay is superior with regard to RFI, thus more sensitive for samples with a low TTV load. Intriguingly, the authors included longitudinal samples from 10 non-immunosuppressed subjects in a time frame of up to 51 months (> 4 years).
Apart from the successful establishment of a highly sensitive and robust TTV qPCR the authors demonstrated that the TTV DNA courses in non-immunosuppressed subjects were either relatively stable over time or showed pronounced changes.
Together, this is a very important finding showing that the personalized TTV levels, their individual variations as well as the mechanism and regulation behind the intra- and inter-individual variability is far from being fully understood and should be adressed in future studies.

There are some suggestions which are listed below.

Introduction:
I would suggest to give the reference „Vietzen et al., 2024" after .... without apparent clinical consequences. (L13 from the top)

Results
2.1.
Comparative analysis of the calculated viral loads revealed no significant differences between the two temperature conditions (Appendix Fig. S1).
R: How many replicates have peen performed? Statistics and Error Bars in the Figure are not stated?

2.1.2.
Among the tested combinations, the n/m primer set showed the best overall performance in terms of Ct values and RFIs, particularly in samples with low viral titers, and was used for subsequent experiments.
R: Please specify, why this primer set is used, because others might be similarly well suited?

2.2:
Intra- and inter-assay precision were evaluated using multiple TTV-DNA-positive plasma samples with high, medium, and low TTV-DNA loads, each analyzed in replicate measurements. Hereby, we observed that low-positive samples were classified as negative because their RFI values were below the standard minimum RFI (RFImin) threshold of 2.5. Therefore, the RFImin was set to 2.0, allowing low-positive samples to be classified as positive. However, this adjustment also increased the risk of false-positive results.
R: Since there are no data shown, it might be better to put this paragraph into the Material and Methods section.

Inter-assay precision (Figure 1A) showed low variability across the tested concentration ranges, with coefficients of variation of 1.16%, 0.95%, and 1.49% for samples with high, medium, and low TTV-DNA load, respectively.
R: For each TTV load only one sample is used? Is this sufficient for firm conclusions?

Samples with high, medium, and low TTV-DNA load also show robust intra-assay precision, with a maximum coefficient of variation of 3.59% across technical triplicates (Figure 1B).
R: For me it is not clear what is meant with 1 to 9 on the x axis.

Linearity of the assay was assessed using two TTV-DNA-positive plasma samples representing different viral load ranges. Both samples were analyzed using a four-step serial dilution (Figure 1C).
R: Please clarify why you used a four-step dilution series as it does not cover the entire range of the TTV load that can be found in clinical samples.

To assess the assay's matrix dependencies, three materials (plasma, serum, and swab) were selected. Negative samples were spiked with the same high TTV-DNA-positive plasma sample.
R: Please clarify, why only one sample was used for the matrix dependancy.

2.3.
Evaluation focused on comparison of Ct values and RFI (Figure EV2).
R: In Figure EV2 patient 1 to 3 instead of pools 1 to 3 are stated.

To further investigate the PCR performance of the three primer/probe sets as well as to evaluate assay specificity and exclude primer dimer formation, all primer pairs were assessed by high-resolution melting analysis (Appendix Fig. S6). Specific TTV-DNA amplification only was observed for both TUM 1 and TUM 3 primer sets. For the TUM 3 primers, an additional non-specific amplicon with a higher melting temperature was detected. No melting peaks indicative of primer dimer formation were observed for any of the tested primer sets.
R: Which or how many samples were used for testing the primer sets? Please specify. HRM is shown in Appendix Fig. S7!

2.3.2.
The TUM 1 primer and probe set shows the best overall PCR performance. Therefore, the primers were modified with 2′-O-methyl groups at the 3′ end. All primer variants were subsequently tested by PCR using all possible forward and reverse primer combinations. PCR performance was evaluated based on Ct values and RFI (Appendix Fig. S7). Three primer modifications showed significantly higher RFI values without an associated increase in Ct values. They were therefore selected for further evaluation in a following experiment using a dilution series (Figure EV3). In this analysis, no significant differences in Ct values were observed between the primer modifications, whereas marked differences in RFI were detected (Figure EV3 B, D, F).
R: Also here, it is not clear which samples were used. Statistics is not mentioned.

2.3.3.
In a subsequent step, the primer and probe concentrations of the TUM 1 (2m/m) primer and probe set were optimized under the same conditions as described previously. Primer concentration was evaluated, with 0.4 µM resulting in the most favourable PCR performance in terms of Ct values and RFI and therefore selected for all experiments (Appendix Fig. S10 A, B).
R: S8!

2.3.4.
For this purpose, nine plasmids containing the TTV target sequence with specific point mutations were generated (Appendix Fig. S10) and analyzed using the TUM 1 (2m/m) qPCR assay (Figure EV4). Mutations 1, 3, and 5 resulted in significantly increased Ct values, indicating reduced primer and probe binding efficiency and decreased detection sensitivity.
R: Statistics? It is also not shown in the Figure.

2.4.
After successful establishment of the TUM-TTV-DNA-qPCR assay on the cobas z 480/LC 480 II, the performance of the TTV LDT was validated on the fully automated cobas 5800. Validation included assessment of intra- and inter-assay precision, linearity, matrix dependency, and analytical specificity and sensitivity. and was performed in accordance with the requirements of the EU In Vitro Diagnostic Regulation (IVDR, EU 2017/746) and the German Rili-BÄK guidelines. The assay exhibited sufficient RFI to allow the RFImin to be raised from the standard 2.5 to 2.7, thereby significantly reducing the likelihood of false-positive sample detection.
R: No data are shown, thus it might be put into the Material and Methods section.

Inter-assay and intra-assay precision were evaluated using TTV-DNA-positive plasma samples covering a broad range of high, medium, and low TTV-DNA load levels (Figure 2 A, B). Inter-assay precision remained robust across the tested concentration ranges, with coefficients of variation of 1.28%, 4.98% and 1.16% for high, medium, and low TTV-DNA load samples. All concentration levels also demonstrated robust intra-assay precision, with a maximum coefficient of variation of 4.25%.
R: Were the same samples used as before?

Linearity of the qPCR assay was assessed using two TTV-DNA-positive plasma samples representing different viral load ranges. Both samples were analyzed using a five-step serial dilution (Figure 2 C). Across the tested dilution range, a linear relationship between input viral load and measured signal was observed, with coefficients of determination (R^2) of 0.9888 and 0.9999, respectively.
R: Why is now a five-step dilution series used? And as mentioned before, the dilution series do not cover the entire clinical TTV range?

To determine the 95% limit of detection (LOD95), a serial dilution series of the TTV plasmid in plasma was prepared, and each dilution was tested repeatedly using the assay on the cobas 5800 system (Figure EV5 B).
R: How often was it tested? Also in the figure legend it is stated „multiple replicates"

The inter-laboratory analysis demonstrated consistent detection, high specificity, and concordant quantification across the tested range, supporting the robustness and reliability of the assay (Appendix Fig. S11).
R: Maybe I got it wrong but in Fig. S11B there were a number of samples that were not detected by all laboratories and also the TTV loads substantially differed?

2.5.
As a last step, both adapted assays, the Maggi et al. qPCR assay and the TUM-TTV-DNA-qPCR, were directly compared for performance on the cobas 5800 system. For this purpose, 96 plasma samples from individual donors were analyzed in parallel using both primer and probe sets (Figure 3 A, B).

R: I would suggest to perform a BLAND and Altman analysis, which in my opinion might better visualize the similarities and differences, at least for the Ct value comparison.

2.6.
R: We have seen the same!

2.7.
Given the limited availability of such data, we analyzed TTV-DNA dynamics over time in ten patients without relevant comorbidities or iatrogenic immunosuppression, approximately every three months (Figure 4, Table 1).
R: I highly appreciate this analysis! It might be interesting to know some basic characteristics of these 10 subjects such as age, gender. Are these subjects patients? And if yes, do they have any underlying diseases? Additionally, it might be worth to compare the TTV courses with the Maggi assay. Finally, it would be interesting to know the TTV sequences and how they evolve over time? Can the authors rule out that the variability is not influenced by the TTV sequence variations?

Discussion

In summary, these studies report an increase in TTV load following SOT and iatrogenic immunosuppression, however, the extent of TTV load increase, baseline characterization, and proposed optimal TTV-DNA values differ between studies, so that the definition of an universal threshold proves to be critical.
R: I am wondering if the definition of an universal threshold is feasible as the kind and the strength of the immunosuppression substantially influences the TTV load. In my opinion it might be more reliable to define a threshold for a given clinical setting.

Giving our data, defining this universal threshold for TTV-DNA levels indicative of adequate immunosuppression remains challenging, as these values must always be interpreted in the context of the individual baseline viral load.
R: With regard to the highly suppressed lung transplant patients it has been shown that the TTV levels that are reached upon immunsuppression post Tx are independent from the baseline differences (DOI: 10.1371/journal.pone.0122975). This could be considered in this regard.

Our findings demonstrate that TTV-DNA exhibits both short-term and long-term intra-individual dynamics even in the absence of immunosuppressive therapy. From the data available in this study it cannot be conclusively determined whether the observed changes reflect modulation of immune function by other factors affecting the immune system besides immunosuppressive medication, or whether they instead represent individual intrinsic variability in viral replication independent of immune alterations.
R: In overall, I agree with the authors. However, I would like to remark that in the SOT setting, particularly in LuTx patients, the TTV DNA load exceeds the TTV load found in non-immunsuppressive people even when, due to the fluctuations, the TTV load can reach 10e6 c/mL. Yet, in other clinical settings the observed „normal" fluctuations may not be distinguishable from medication-induced TTV load changes.

This individual "anellome" remains largely stable during adulthood (Timmerman et al., 2024, Kaczorowska et al., 2022), therefore, new infections with different viral variants are unlikely to underlie the observed interindividual fluctuations.
R: I am not sure if there are already enough data to state that new infections are unlikely.

Referee #1 (Comments on Novelty/Model System for Author):

While the assay development is technically sound and potentially useful for standardization, the limited cohort size and lack of immunological context restrict the biological and translational insight, preventing the study from reaching the level expected for EMBO Molecular Medicine.

Referee #1 (Remarks for Author):

Lara Valder and collegues report the development of a very robost and interesting high-throughput qPCR assay for TTV detection and apply it to longitudinal monitoring in non-immunosuppressed individuals. The assay is technically solid and represents a useful step toward standardization. However, the biological conclusions are derived from a very limited dataset and lack mechanistic support. As a result, the manuscript remains largely descriptive and does not provide sufficient conceptual or translational advance for EMBO Molecular Medicine.

Major Comments

1. Biological conclusions are not supported by the dataset

The manuscript states:

"Longitudinal analysis... revealed pronounced inter- and intra-individual variability" and "pronounced longitudinal fluctuations in TTV-DNA levels in patients without iatrogenic immunosuppression"

These statements are central to the manuscript but rely on o.nly 10 individuals (Fig. 4). The variability observed-while interesting-cannot be generalized and may reflect sampling noise or uncontrolled confounders. The study therefore supports an observation, not a biological principle.

We appreciate the reviewer's concern regarding the generalizability of our initial findings and have revised the corresponding sections of the manuscript to clarify that pronounced fluctuations in TTV-DNA levels were observed only in a subset of subjects and not in every individual.

Our results show, that in the original cohort, 3 of 10 individuals (30%) without iatrogenic immunosuppression exhibited changes in TTV-DNA levels exceeding 1 $\log_{10}$ within approximately three months. Specifically, subject 6 and subject 9 showed changes of almost 2.5 $\log_{10}$ and 2 $\log_{10}$, respectively, between months 9 and 12 and between months 27 and 30. In addition, these subjects displayed further undulating changes of more than 1 $\log_{10}$ during longitudinal follow-up. Subject 8 showed a change of approximately 1.5 $\log_{10}$ between months 0 and 3, although this individual also showed an overall tendency toward increasing TTV-DNA levels over the study period, with an overall increase of 2.9 $\log_{10}$. Based on these observations, we used the terms “pronounced inter- and intra-individual variability” and “pronounced longitudinal fluctuations.”

To address the reviewer's concern regarding potential technical explanations for these fluctuations, we demonstrated in Figure 2 the high analytical precision of our assay, with a maximum coefficient of variation of 4.98% for really low positive samples, indicating that the observed variability in TTV-DNA loads in the three individuals are unlikely to be attributable to analytical imprecision. We also systematically assessed potential pre-analytical influences, including storage temperature, storage duration and matrix dependencies, and did not observe any significant effects on measured TTV-DNA levels (Figure EV6). Therefore, we do not consider both assay imprecision and pre-analytical factors to account for the observed longitudinal changes. Rather, the data support that these fluctuations represent true intra-individual changes in TTV-DNA levels in a subset of individuals.

To further strengthen the robustness of our findings, we analyzed an independent longitudinal cohort comprising 11 additional individuals, which is now presented in Appendix Fig. S14. In this cohort, we

again observed TTV-DNA fluctuations exceeding 1 log_{10} within approximately three months in three individuals (subjects 11, 12, and 16), with changes of approximately 1.2 log_{10}, 1.0 log_{10}, and 1.6 log_{10}, respectively. Thus, also in this independent cohort, approximately one third of individuals exhibited TTV-DNA fluctuations exceeding 1 log_{10} within one quarter. These findings support our conclusion that pronounced longitudinal variability in TTV-DNA levels is not restricted to the initially analyzed cohort but can also be observed in an independent subset of individuals without iatrogenic immunosuppression.

Finally, to exclude assay-specific variation as an explanation, we re-tested samples from three selected individuals, subject 12, subject 16, and subject 17, using two additional assays, including a new commercially available assay (Appendix Fig. S16). These analyses showed parallel longitudinal patterns across assays, including the pronounced fluctuations observed in subjects 12 and 16. While the absolute viral loads differed between the assays, the longitudinal dynamics and pronounced variability were consistent.

In summary, the aim of our manuscript is to highlight that, although TTV-DNA levels may remain stable over time in many individuals, relevant fluctuations can occur in some individuals without iatrogenic immunosuppression, which is the clinical setting in which TTV is intended to be used as a biomarker. According to the reviewers suggestion, we have therefore revised the manuscript to emphasize that these fluctuations should not be generalized as a universal observation, but rather represent a finding observed in a subset of the analyzed subjects. This has now been clarified at the positions indicated by the reviewer, particularly in the abstract, results part and discussion.

2. Absence of immunological characterization precludes interpretation

The authors explicitly acknowledge:

"it cannot be conclusively determined whether the observed changes reflect modulation of immune function... or... intrinsic variability"

This is a critical limitation. Without immune profiling or clinical context, the study cannot distinguish between: immune-driven modulation, intercurrent infections and other factors such as intrinsic viral dynamics. As a consequence, the manuscript does not provide mechanistic insight into TTV biology or its use as a biomarker.

We fully agree with the reviewer that identifying the underlying causes of these individual fluctuations would be of great value, especially for the general and, in particular, standardized use of TTV as a biomarker in immunocompromised patients. We also agree that the observed changes may reflect a general modulation of immune function, intrinsic viral variability, other host- or environment-related factors, or a combination of these mechanisms.

However, the present study was designed to develop a fully automated, high-throughput TTV-DNA qPCR assay and to use this method to analyze TTV viral loads in large numbers of cohort and clinical patient samples, including longitudinal samples. This approach was intended to assess TTV viral load dynamics, including abnormal or unexpected TTV-DNA patterns, and thereby support the establishment of TTV-DNA quantification as a standardized biomarker. In our study, we show that pronounced TTV-DNA fluctuations can occur in a subset of individuals without iatrogenic immunosuppression and that this observation has important implications for the use of TTV as a biomarker of iatrogenic immunosuppression.

More detailed molecular analyses, such as comprehensive immune profiling or mechanistic investigations into the biology and regulation of TTV replication, are undoubtedly important. However, such analyses fall beyond the scope and primary objectives of the current study and would require a

dedicated study specifically designed to address these questions. Larger prospective cohort studies including immune profiling, detailed clinical metadata, and assessment of intercurrent infections will be required to determine whether the observed fluctuations reflect immune-driven modulation, intrinsic viral dynamics, or other host- or environment-related factors.

In summary, we acknowledge that this aspect is of high importance. Our study provides the methodological basis for further investigations and highlights this important observation to the scientific community, thereby encouraging further and more broadly designed studies addressing these questions. As discussed in the revised discussion, we now explicitly consider this an important direction for future research.

3. Overstated implications for TTV as biomarker

The manuscript repeatedly suggests clinical relevance: "important implications for the interpretation of TTV-DNA as a biomarker".

However, no direct link to immune status is demonstrated. In fact, the data undermine interpretability without offering a solution. This creates a disconnect between data and claims.

We acknowledge the reviewer's concern, which, as mentioned in our previous response, cannot be fully resolved within the scope of the present study, as it would require a further large-scale, prospective investigation that was not an objective of the current work.

In our view, the clinical relevance of the present findings lies in the observation that substantial intra-individual fluctuations in TTV-DNA levels can occur. The possibility of such variability in a subject of patients should therefore be taken into account in future TTV studies, as well as in the definition of clinically meaningful thresholds for the use of TTV as a biomarker of iatrogenic immunosuppression. Importantly, individuals exhibiting such high intra-individual variability are likely to be present in most cohorts, including patients undergoing or scheduled to undergo iatrogenic immunosuppression. Such variability may therefore influence study outcomes, the interpretation of longitudinal TTV measurements, and, ultimately, individualized adjustment of immunosuppressive therapy, which represents the intended clinical use of TTV as a biomarker in many settings.

Accordingly, we believe that our findings highlight an important aspect that must be considered when using TTV-DNA as a biomarker for immunosuppression and immunosuppressive therapy. In response to the reviewer's comment, we have revised the discussion to more clearly emphasize the clinical implications of our findings. In particular, we now emphasize that, especially in patients with relatively low TTV-DNA fluctuations of approximately 1 $\log_{10}$ should not be automatically attributed to iatrogenic immunosuppression, as has been suggested in some previous studies.

4. Insufficient consideration of anellovirus complexity

The assay targets a conserved UTR region and is claimed to cover:

"more than 95% of the evaluated TTV sequence variants"

However:

This is based on consensus/in silico design and limited synthetic constructs

No validation is performed on real multi-genotype samples

Given the complexity of the human "anellome," the assay likely captures only part of the viral landscape. This limitation directly affects interpretation of "TTV load" as a biological variable.

We thank the reviewer for this comment and agree that the human anellome consists of substantial TTV heterogeneity and cannot be completely captured by a single PCR assay.

However, in our study, we designed the primer and probe sequences to cover a broad range of known sequenced TTV variants. This coverage is based on the alignment of more than 600 TTV sequences comprising diverse genotypes and is therefore expected to represent a substantial proportion of the currently known biological TTV diversity. In addition, the primers and probe target a highly conserved region within the UTR, which has been reported in several studies to exhibit comparatively low sequence variability across TTV strains. This high degree of conservation is further supported by our alignment results.

In the manuscript, we specifically addressed sequence variability within the target region and experimentally analyzed relevant mutations using synthetic constructs. Based on these analyses, we conclude that our PCR assay is expected to detect more than 95% of the fully sequenced TTV genomes available in the NCBI database that were included in our analysis.

We interpret the reviewer's concern as the possibility that fluctuations of individual TTV genotypes or variants may lead to under- or over-quantification of the total anellovirus concentration at individual time points and may thereby explain the observed longitudinal fluctuations. To address this concern, as stated in the previous comment, we selected longitudinal samples from three individuals showing different TTV-DNA dynamics, including low, intermediate, and high variability, and re-analyzed these samples using two additional assays: the *Maggi et al.* PCR assay and a recently available commercial TTV PCR assay. Across all three assays, we observed parallel longitudinal TTV-DNA kinetics, including the relevant fluctuations in subject 12 and subject 16, as well as stable TTV-DNA levels in subject 17. Although the absolute viral loads differed between assays, the longitudinal patterns were consistent.

Overall, these addition results in the revised manuscript indicate that the observed longitudinal changes are unlikely to be explained solely by assay-specific detection of individual TTV genotypes or variants. Instead, they suggest that the overall TTV-DNA concentration changes periodically or over time, rather than only individual variants being differentially detected by a single assay, in particular in adults as included in our study. Therefore, although we agree that a single PCR assay cannot capture the complete complexity of the human anellome, our data support that the use of targeted broad covering TTV qPCR assays, as currently applied in the field, is appropriate for assessing longitudinal TTV-DNA dynamics.

5. Lack of essential clinical metadata

The cohort is described as:

"patients without relevant comorbidities"

but no supporting data are provided (age, sex, clinical background, infections, treatments). This severely limits interpretability and reproducibility.

We agree with the reviewer that the initial clinical characterization of the subjects in our original cohort was not sufficiently detailed in the original manuscript.

In response to the reviewer's comments, we have now included all relevant clinical information that was available within the scope of the ethics approval for the original retrospective cohort. Specifically, we have incorporated information on age, sex, and HIV status, as HIV infection may be associated with immunosuppression.

6. Conceptual imbalance: strong methods, weak biology

The assay validation is rigorous (Fig. 1-3), including: precision, linearity, inter-laboratory comparison. However, the biological component (Fig. 4) is: underpowered, uncontrolled and purely descriptive. This imbalance prevents the manuscript from reaching the level of conceptual advance expected for this journal.

We thank the reviewer for acknowledging the rigorous assay development, validation, and precision analyses presented in our study. We also agree with the reviewer's concern that the initial longitudinal cohort of 10 subjects represents a relatively small cohort.

As stated above, we have now expanded the study by including a second longitudinal cohort of 11 additional subjects. In this second cohort, we again observed relevant TTV-DNA fluctuations exceeding 1 $\log_{10}$ in approximately 30% of individuals. Thus, the extension of the cohort strengthens the power and interpretability of our study and supports the conclusion that such longitudinal fluctuations are not restricted to the initially analyzed subjects.

Minor Comments

The statement that TTV is "relatively stable over time" in prior studies is not quantitatively reconciled with the present findings.

We agree with the reviewer that our data shows that TTV-DNA levels are not uniformly stable in healthy individuals, in contrast to what has been suggested by the limited number of longitudinal studies addressing this question. We have now expanded the discussion to more clearly address the discrepancy between the available literature and our findings.

Importantly, the scarce previously published longitudinal studies were based either on very small cohorts (e.g., only two individuals) or on sampling intervals that were substantially longer, with measurements performed approximately 10 years apart. Consequently, the short-term fluctuations observed in approximately 30% of subjects in our cohort would likely not have been detected or adequately characterized in these studies. It should also be noted that these studies were designed to address different primary research questions. The discrepancy between our findings and the currently available literature has now been explicitly discussed in the revised manuscript.

The discussion of RFI thresholds (Sections 2.2 vs 2.4) would benefit from clearer rationale.

We thank the reviewer for this suggestion. We have now emphasized the rationale for the selected RFI thresholds in the text and in Figure 3. In addition, we have included Appendix Fig. S13, which shows a false-positive sample obtained with the *Maggi et al.* assay at an RFI_{min} of 1.5, likely due to unspecific amplification.

The claim of "no relationship" between viral load and variability should be supported by statistical analysis.

We thank the reviewer for this comment. The statement regarding the relationship between viral load and variability was intended to indicate that variability was observed both in subjects with low average viral loads and in subjects with higher average viral loads. Thus, we did not observe an apparent association between mean TTV-DNA load and the magnitude of intra-individual variability.

We acknowledge the reviewers comment and have now performed statistical analyses for the respective figures and added the corresponding information to the figure legends.

It would be nice if the authors trued to analyze also full blood rather than plasma.

We agree with the reviewer that whole blood, in addition to serum and plasma, would be a relevant sample material for TTV-DNA testing. However, one aim of our study was to establish a high-

throughput assay on a fully automated diagnostic platform. Therefore, we implemented the assay on the cobas x800 system. Unfortunately, there are currently no established and validated manufacturer-provided protocols for whole blood samples on this system.

Suggested Experiments / Analyses to substantially strengthen the manuscript:

Correlate TTV load with immune parameters (even retrospective data would help)

Screen for co-infections (e.g., CMV, EBV) to contextualize fluctuations

Assess genotype composition (NGS or genotype-resolved assays)

Define technical vs biological variability (short-interval replicates)

Validate findings in an independent cohort

We thank the reviewer for suggesting these additional experiments. In the revised manuscript, we have performed the suggested analyses where feasible and appropriate for the aims of the study, in particular the validation of our results in a second independent cohort. Points that could not be experimentally addressed within the scope of the present study have been discussed in the respective responses above. In addition, we have included all relevant clinical information available under the constraints of the ethical approval, including data on co-infections, in particular HBV, HCV, and HIV.

Conclusion

This is a well-executed assay development study with clear technical merit. However, the biological component is underdeveloped and does not provide sufficient mechanistic or translational insight. Significant expansion of the cohort and integration of immunological context would be required to support the proposed conclusion and increase the utility of this study.

We thank the reviewer for the detailed comments, in particular for this concluding assessment. We especially appreciate the reviewer's recognition of the rigorous development and validation of our assay. We hope that we have adequately addressed the concerns about our conclusion raised by the reviewer and answered them to their satisfaction.

Referee #2 (Comments on Novelty/Model System for Author):

The work is well executed and analytically robust, with clear added value in standardising and automating TTV quantification. There are minor methodological limitations that can be easily addressed. Deployment of the assay to evaluate viral concentrations in longitudinal plasma samples from patients confirmed previous observations and did not, in itself, introduce any novelties.

Referee #2 (Remarks for Author):

This study describes the development and analytical validation of a high-throughput qPCR assay for Torque teno virus (TTV) implemented on fully automated platforms (Roche Cobas x800). The work is well conducted and methodologically solid, demonstrating robust analytical performance, including good precision, linearity, sensitivity, and lack of matrix effects. The newly developed assay outperforms Maggi's reference TTV assay in terms of signal intensity and low-level detection. Importantly and as previously demonstrated by other groups in independent studies, longitudinal analysis in immunocompetent individuals reveals substantial intra- and inter-individual variability in TTV viral load, highlighting limitations in the interpretation of TTV as a biomarker of immune status.

Overall, the study is interesting and relevant, particularly because it validates a TTV qPCR assay on automated systems such as Cobas supporting its potential use in routine diagnostics and large-scale studies.

Major issues

• The main concern relates to the generation of the consensus sequence used for primer design. The authors state that sequences were retrieved from NCBI Virus and aligned (MAFFT), but do not sufficiently describe:

o the query used to retrieve sequences,

o how they ensured that sequences were truly TTV (and not TTMV/TTMDV, given known annotation ambiguities in Anelloviridae databases),

o whether sequences were filtered (e.g., completeness, redundancy, duplicates),

o whether MAFFT alignment was performed on full genomes or restricted regions (e.g., UTR).

These methodological gaps limit reproducibility and should be clarified.

We thank the reviewer for this valuable suggestion. We agree that a more detailed description of the consensus sequence generation is important to ensure the transparency and reproducibility of our primer design strategy. Accordingly, we have substantially expanded the corresponding section in the revised manuscript.

Specifically, we have added a detailed description of the sequence retrieval strategy, including the NCBI Virus search query used, the criteria applied to ensure that only TTV sequences (excluding TTMV and TTMDV) were included despite known annotation ambiguities within the Anelloviridae family, the filtering steps performed to remove incomplete and redundant sequences, and the genomic region used for the MAFFT alignment. These additions have been incorporated in the revised manuscript in Section 4.2 of the methods part.

• Page 6, Section 2.4, second paragraph. Please state the TTV DNA concentrations used for inter- and intra-assay precision

We thank the reviewer for this suggestion. Since the assay validation was performed before the quantification study, the patient samples used for assay validation had not been precisely quantified at that time. We have now retrospectively quantified these samples and added the corresponding viral loads to section 4.1 of the Materials and Methods.

• Page 7, Section 2.6. The sentence describing plasma stability should be modified to clearly state that nine days was the observed period, as the data explicitly show stability "for up to nine days" under different storage conditions.

We thank the reviewer for this suggestion and have rewritten the sentence accordingly to state more clearly that a nine-day observation period was analyzed.

Minor issues

• Several minor writing and punctuation errors are present throughout the manuscript and should be corrected, e.g. Magi that should be replaced with Maggi in Table 2, Appendix-Suppl. File.

We thank the reviewer for thoroughly reading the manuscript and for drawing our attention to these errors. We have performed an additional proofreading of the manuscript and corrected the remaining spelling and grammatical issues. We also checked all occurrences of Maggi's name and corrected them where necessary.

Referee #3 (Comments on Novelty/Model System for Author):

While stated in the text that there are statistically significant differences/no differences the statistics is not always given.

Bland and altman analysis in Fig 3A might be better for comparison.

We thank the reviewer for this valuable suggestion. We agree that statistical analyses should be provided wherever statements regarding significant or non-significant differences are made. In response, we have revised the manuscript and added the corresponding statistical analyses to all experiments. The applied statistical tests and significance levels are now included in the figures, figure legends, and the corresponding sections of the results.

In addition, we agree that a Bland–Altman analysis represents a more appropriate method for assessing agreement between measurements than correlation analysis alone. Therefore, we have performed a Bland–Altman analysis for the comparison shown in Figure 3A and included the resulting plot and interpretation in the revised manuscript.

While it is highly interesting to see the different TTV courses in the 10 subjects there are no further investigations (seq info) to give some potential explanations.

We fully agree with the reviewer that our observation has important implications for the standardized use of TTV as a biomarker in immunocompromised patients, and that further investigation of the specific underlying causes or potential contributing factors would be highly valuable. However, the primary objective of the present study was to establish a fully automated, high-throughput TTV-DNA qPCR assay and to apply this method to the analysis of TTV viral loads in large numbers of cohort and clinical patient samples, including longitudinal samples, in which we observed the described fluctuations.

An assessment of potential influencing factors or mechanistic explanations would currently remain speculative without robust supporting data. In addition to infections, comorbidities, dietary changes, or other intrinsic factors, TTV viral load may vary depending on immune status, but it may also fluctuate due to factors independent of immune function. Therefore, we aimed to restrict our conclusions to the factual observation that pronounced TTV-DNA fluctuations can occur in a subset of individuals without iatrogenic immunosuppression. Since such fluctuations in TTV-DNA load may also be present in broader clinical cohorts, including patients undergoing or scheduled to undergo iatrogenic immunosuppression, this finding should be considered when interpreting longitudinal TTV measurements in these patients.

In summary, we agree that more detailed molecular analyses, including sequencing, comprehensive immune profiling, and mechanistic studies on the biology and regulation of TTV replication, would be important to explain these individual longitudinal courses. Such analyses, however, would require a dedicated prospective study specifically designed to address this question. We have now updated the discussion in the revised manuscript to emphasize that further studies with additional parameters are required to elucidate the potential underlying mechanisms.

Referee #3 (Remarks for Author):

The study with the title „A high-throughput qPCR for Torque teno virus reveals pronounced longitudinal viral load variability" submitted by Lara Valder and collegues established a high-throughput TTV qPCR assay in comparison to the Maggi et al., TTV assay and tested sensitivity, specificity and applicability on a set of TTV positive samples. While both assays are comparable, the

newly established TUM TTV assay is superior with regard to RFI, thus more sensitive for samples with a low TTV load. Intriguingly, the authors included longitudinal samples from 10 non-immunosuppressed subjects in a time frame of up to 51 months (> 4 years).

Apart from the successful establishment of a highly sensitive and robust TTV qPCR the authors demonstrated that the TTV DNA courses in non-immunosuppressed subjects were either relatively stable over time or showed pronounced changes.

Together, this is a very important finding showing that the personalized TTV levels, their individual variations as well as the mechanism and regulation behind the intra- and inter-individual variability is far from being fully understood and should be adressed in future studies.

There are some suggestions which are listed below.

Introduction:

I would suggest to give the reference „Vietzen et al., 2024" after .... without apparent clinical consequences. (L13 from the top)

We thank the reviewer for this helpful suggestion and have added the suggested reference following the respective sentences in the revised manuscript.

Results

2.1.

Comparative analysis of the calculated viral loads revealed no significant differences between the two temperature conditions (Appendix Fig. S1).

R: How many replicates have peen performed? Statistics and Error Bars in the Figure are not stated?

We agree with the reviewer that statistical analyses were missing in some parts of the manuscript. The measurements were performed in triplicates, and statistical significance was assessed using a paired t-test. The corresponding details have now been added to the figures, including the figure legends, as well as to the Methods section where appropriate. In addition, we have revised the whole manuscript accordingly to include all requested information consistently throughout the text and figures.

2.1.2.

Among the tested combinations, the n/m primer set showed the best overall performance in terms of Ct values and RFIs, particularly in samples with low viral titers, and was used for subsequent experiments.

R: Please specify, why this primer set is used, because others might be similarly well suited?

We thank the reviewer for this important comment and agree that the rationale for selecting the most suitable primer combination was not clearly described in the original manuscript, as the results were very similar and no significant differences in Ct values or RFI were observed.

Since the n/m primer combination showed a slight trend toward lower Ct and higher RFI values, particularly in samples with low viral loads, we selected this combination for subsequent experiments. Although alternative primer combinations performed similarly and would likely also be suitable for future experiments, the n/m primer set demonstrated the most consistent overall performance across experiments.

To address the reviewer's comment, we have therefore clarified the rationale for the final primer selection in the revised manuscript.

2.2:

Intra- and inter-assay precision were evaluated using multiple TTV-DNA-positive plasma samples with high, medium, and low TTV-DNA loads, each analyzed in replicate measurements. Hereby, we observed that low-positive samples were classified as negative because their RFI values were below the standard minimum RFI (RFImin) threshold of 2.5. Therefore, the RFImin was set to 2.0, allowing low-positive samples to be classified as positive. However, this adjustment also increased the risk of false-positive results.

R: Since there are no data shown, it might be better to put this paragraph into the Material and Methods section.

We thank the reviewer for this comment and have now included the corresponding information in the revised manuscript. Specifically, we have added the range of RFI values observed for the low-positive samples in the relevant section and provided a more detailed explanation.

Inter-assay precision (Figure 1A) showed low variability across the tested concentration ranges, with coefficients of variation of 1.16%, 0.95%, and 1.49% for samples with high, medium, and low TTV-DNA load, respectively.

R: For each TTV load only one sample is used? Is this sufficient for firm conclusions?

We thank the reviewer for raising this important point and would like to emphasize that the validation was performed in accordance with the requirements of the European In Vitro Diagnostic Regulation (IVDR, EU 2017/746) and the German Rili-BÄK guidelines.

According to these guidelines, assessment of inter-assay precision is performed using one representative sample per viral load range measured on three consecutive days. In contrast, a larger number of replicate measurements is required for the assessment of intra-assay precision in order to adequately evaluate variability within a single analytical run.

Samples with high, medium, and low TTV-DNA load also show robust intra-assay precision, with a maximum coefficient of variation of 3.59% across technical triplicates (Figure 1B).

R: For me it is not clear what is meant with 1 to 9 on the x axis.

We thank the reviewer for the constructive feedback and agree that the previous sample nomenclature may have been misleading. To clarify that high-, medium-, and low-positive samples were used for each validation experiment, we have now described this more clearly in the text and figures.

In the *Maggi et al.* assay validation shown in Figure 1, the samples are now labeled as H1-H3, M1-M3, and L1-L3. Since different samples were used for the validation of the TUM assay, these samples are now labeled in the text and in Figure 2 as H4-H6, M4-M6, and L4-L6.

Linearity of the assay was assessed using two TTV-DNA-positive plasma samples representing different viral load ranges. Both samples were analyzed using a four-step serial dilution (Figure 1C).

R: Please clarify why you used a four-step dilution series as it does not cover the entire range of the TTV load that can be found in clinical samples.

We appreciate the reviewer's comment. As described above, the validation was performed in accordance with the European In Vitro Diagnostic Regulation (IVDR, EU 2017/746) and the German

Rili-BÄK guidelines. These guidelines recommend four dilution steps for the assessment of assay linearity.

To assess the assay's matrix dependencies, three materials (plasma, serum, and swab) were selected. Negative samples were spiked with the same high TTV-DNA-positive plasma sample.

R: Please clarify, why only one sample was used for the matrix dependancy.

We thank the reviewer for this comment. This experiment was also conducted in accordance with the requirements of the European In Vitro Diagnostic Regulation (IVDR, EU 2017/746) and the German Rili-BÄK guidelines. For the assessment of matrix dependence, the relevant matrices are selected for comparison and are each spiked once with a high-positive and once with a low-positive sample. According to these guidelines, no additional samples are required for this type of evaluation.

2.3.

Evaluation focused on comparison of Ct values and RFI (Figure EV2).

R: In Figure EV2 patient 1 to 3 instead of pools 1 to 3 are stated.

We completely agree with the reviewer. We have changed the label in the figure to "pool," as pooled samples from different patients were used.

To further investigate the PCR performance of the three primer/probe sets as well as to evaluate assay specificity and exclude primer dimer formation, all primer pairs were assessed by high-resolution melting analysis (Appendix Fig. S6). Specific TTV-DNA amplification only was observed for both TUM 1 and TUM 3 primer sets. For the TUM 3 primers, an additional non-specific amplicon with a higher melting temperature was detected. No melting peaks indicative of primer dimer formation were observed for any of the tested primer sets.

R: Which or how many samples were used for testing the primer sets? Please specify. HRM is shown in Appendix Fig. S7!

We thank the reviewer for raising this point. The figures in the Appendix were mistakenly swapped, and this has now been corrected.

In addition, the samples used are now clearly described in the main text. Specifically, a sample consisting of pooled plasma from 10 subjects was used and measured in triplicate.

2.3.2.

The TUM 1 primer and probe set shows the best overall PCR performance. Therefore, the primers were modified with 2'-O-methyl groups at the 3' end. All primer variants were subsequently tested by PCR using all possible forward and reverse primer combinations. PCR performance was evaluated based on Ct values and RFI (Appendix Fig. S7). Three primer modifications showed significantly higher RFI values without an associated increase in Ct values. They were therefore selected for further evaluation in a following experiment using a dilution series (Figure EV3). In this analysis, no significant differences in Ct values were observed between the primer modifications, whereas marked differences in RFI were detected (Figure EV3 B, D, F).

R: Also here, it is not clear which samples were used. Statistics is not mentioned.

We thank the reviewer for this suggestion and have now also added additional context regarding the samples used in the main text. Statistical analyses are now also presented in the figure and described in the corresponding figure legend. As in previous experiments, a pooled plasma sample derived from

10 TTV-positive individuals was used. For statistical evaluation, a paired t-test was performed to compare the different combinations.

2.3.3.

In a subsequent step, the primer and probe concentrations of the TUM 1 (2m/m) primer and probe set were optimized under the same conditions as described previously. Primer concentration was evaluated, with 0.4 µM resulting in the most favourable PCR performance in terms of Ct values and RFI and therefore selected for all experiments (Appendix Fig. S10 A, B).

R: S8!

We thank the reviewer for carefully reading the manuscript and for this helpful comment. We have revised the manuscript accordingly.

2.3.4.

For this purpose, nine plasmids containing the TTV target sequence with specific point mutations were generated (Appendix Fig. S10) and analyzed using the TUM 1 (2m/m) qPCR assay (Figure EV4). Mutations 1, 3, and 5 resulted in significantly increased Ct values, indicating reduced primer and probe binding efficiency and decreased detection sensitivity.

R: Statistics? It is also not shown in the Figure.

We agree with the reviewer that the manuscript would benefit from more statistical analyses. We have therefore performed statistical analyses where possible. For this figure, we performed a paired t-test and have now included this information in the figure. The figure legend has also been expanded accordingly.

2.4.

After successful establishment of the TUM-TTV-DNA-qPCR assay on the cobas z 480/LC 480 II, the performance of the TTV LDT was validated on the fully automated cobas 5800. Validation included assessment of intra- and inter-assay precision, linearity, matrix dependency, and analytical specificity and sensitivity. and was performed in accordance with the requirements of the EU In Vitro Diagnostic Regulation (IVDR, EU 2017/746) and the German Rili-BÄK guidelines. The assay exhibited sufficient RFI to allow the RFImin to be raised from the standard 2.5 to 2.7, thereby significantly reducing the likelihood of false-positive sample detection.

R: No data are shown, thus it might be put into the Material and Methods section.

We thank the reviewer for this comment. As mentioned above, we have now included the RFI range of the samples and expanded the rationale for the selected RFI_{min} threshold, so that this paragraph is better integrated into the results section.

Inter-assay and intra-assay precision were evaluated using TTV-DNA-positive plasma samples covering a broad range of high, medium, and low TTV-DNA load levels (Figure 2 A, B). Inter-assay precision remained robust across the tested concentration ranges, with coefficients of variation of 1.28%, 4.98% and 1.16% for high, medium, and low TTV-DNA load samples. All concentration levels also demonstrated robust intra-assay precision, with a maximum coefficient of variation of 4.25%.

R: Were the same samples used as before?

We agree with the reviewer that the previous description of the samples was not sufficiently clear and could be misleading. Different samples were used for the respective validation experiments, and, as mentioned above, we have now stated this more clearly in both the text and the figures. In the *Maggi*

et al. assay validation shown in Figure 1, the samples are now labeled as H1-H3, M1-M3, and L1-L3. Since different samples were used for the validation of the TUM assay, these samples are now labeled in the text and in Figure 2 as H4-H6, M4-M6, and L4-L6.

Linearity of the qPCR assay was assessed using two TTV-DNA-positive plasma samples representing different viral load ranges. Both samples were analyzed using a five-step serial dilution (Figure 2 C). Across the tested dilution range, a linear relationship between input viral load and measured signal was observed, with coefficients of determination (R^2) of 0.9888 and 0.9999, respectively.

R: Why is now a five-step dilution series used? And as mentioned before, the dilution series do not cover the entire clinical TTV range?

We thank the reviewer for this comment. As described above, the validation, including the assessment of linearity, was performed in accordance with the European In Vitro Diagnostic Regulation (IVDR, EU 2017/746) and the German Rili-BÄK guidelines. According to these requirements, a four-step dilution series is used for the assessment of assay linearity.

For the TUM assay, we included a fifth dilution step because the high- and low-positive patient samples used for this experiment had higher initial starting concentrations.

To determine the 95% limit of detection (LOD95), a serial dilution series of the TTV plasmid in plasma was prepared, and each dilution was tested repeatedly using the assay on the cobas 5800 system (Figure EV5 B).

R: How often was it tested? Also in the figure legend it is stated „multiple replicates"

We thank the reviewer for raising the point that the number of replicates used in this experiment, 15 replicates, was missing from the original manuscript. We have now added this information to the revised manuscript.

The inter-laboratory analysis demonstrated consistent detection, high specificity, and concordant quantification across the tested range, supporting the robustness and reliability of the assay (Appendix Fig. S11).

R: Maybe I got it wrong but in Fig. S11B there were a number of samples that were not detected by all laboratories and also the TTV loads substantially differed?

We agree with the reviewer that the quantification results of the inter-laboratory comparison are noticeable at first glance.

As described in the manuscript, each laboratory included in the inter-laboratory comparison used its own individually designed and validated assay, with assay-specific quantification procedures. In addition, the samples were extracted using different workflows and analyzed on different instruments with different master mixes, so that variations in the results are expected.

The main objective of this comparison was not to evaluate absolute quantification across the different assays, but rather to assess the analytical sensitivity and specificity of our assay. In this context, high- and low-positive samples were used to assess sensitivity, whereas negative samples were used to assess specificity. Specifically, we aimed to determine whether samples classified as positive by our assay were indeed true positives and whether negative samples were correctly identified as negative.

This inter-laboratory comparison allowed us to confirm both the sensitivity and specificity of our assay. Notably, the assay from Freiburg failed to detect several of the low-positive samples, which may indicate lower analytical sensitivity compared with the other assays evaluated, since the low-positive samples originated from high-positive TTV sample dilutions.

To address the reviewer's point more clearly in the manuscript, we have now included more detailed information and explanation in the revised manuscript.

2.5.

As a last step, both adapted assays, the Maggi et al. qPCR assay and the TUM-TTV-DNA-qPCR, were directly compared for performance on the cobas 5800 system. For this purpose, 96 plasma samples from individual donors were analyzed in parallel using both primer and probe sets (Figure 3 A, B).

R: I would suggest to perform a BLAND and Altman analysis, which in my opinion might better visualize the similarities and differences, at least for the Ct value comparison.

We thank the reviewer for this helpful suggestion. We have implemented the proposed analysis and incorporated the corresponding information into both the manuscript text and the figure.

2.6.

R: We have seen the same!

We thank the reviewer for this comment and for confirming the validity of our data.

2.7.

Given the limited availability of such data, we analyzed TTV-DNA dynamics over time in ten patients without relevant comorbidities or iatrogenic immunosuppression, approximately every three months (Figure 4, Table 1).

R: I highly appreciate this analysis! It might be interesting to know some basic characteristics of these 10 subjects such as age, gender. Are these subjects patients? And if yes, do they have any underlying diseases? Additionally, it might be worth to compare the TTV courses with the Maggi assay. Finally, it would be interesting to know the TTV sequences and how they evolve over time? Can the authors rule out that the variability is not influenced by the TTV sequence variations?

We thank the reviewer for appreciating our findings and their potential impact on the clinical use of TTV as a novel biomarker.

We acknowledge the concern regarding the missing basic characteristics of the study cohort and have now included all available patient-related information that could be obtained from the retrospective samples in accordance with the approved ethics, including demographic parameters such as age and sex.

In addition, we included a second cohort of 11 subjects, now shown in Appendix Fig. S14. In this cohort, we again observed TTV-DNA fluctuations exceeding 1 $\log_{10}$ within approximately three months in 3 individuals: subject 11, subject 12, and subject 16. This supports our statement that longitudinal TTV-DNA variability can occur in individuals without immunosuppressive therapy.

We highly appreciate the suggestion that comparing the longitudinal courses across different assays, especially in subjects with pronounced fluctuations, would substantially strengthen the manuscript and our conclusion. We therefore tested samples from three selected individuals, one without and two with pronounced fluctuations, subject 12, subject 16, and subject 17, using two additional assays: the *Maggi et al.* assay and a newly commercially available assay. These analyses showed parallel longitudinal patterns across assays, in particular the pronounced fluctuations observed in subjects 12 and 16. While the absolute viral loads differed between assays, the longitudinal dynamics were consistent. This indicates that the observed fluctuations and variations are not assay-specific findings, but can be reproduced using independent assays.

We fully agree that sequencing of the underlying TTV variants would be of great value. However, the present study was designed to develop a fully automated, high-throughput TTV-DNA qPCR assay and to use this method to analyze TTV viral loads in large numbers of cohort and clinical patient samples, including longitudinal samples. The evolution of TTV sequences and their potential influence on longitudinal viral load dynamics should be addressed in larger prospective cohort studies with appropriate ethical approval for NGS-based analyses.

Based on our data, however, evolution or variation of TTV sequences is unlikely to be the sole underlying explanation for the observed fluctuations, since all assays showed comparable longitudinal patterns. In addition, all assays target the UTR, which is highly conserved, and the primer/probe binding sites of the different assays are therefore unlikely to be affected in a way that would explain the parallel fluctuations observed across assays.

Discussion

In summary, these studies report an increase in TTV load following SOT and iatrogenic immunosuppression, however, the extent of TTV load increase, baseline characterization, and proposed optimal TTV-DNA values differ between studies, so that the definition of an universal threshold proves to be critical.

R: I am wondering if the definition of an universal threshold is feasible as the kind and the strength of the immunosuppression substantially influences the TTV load. In my opinion it might be more reliable to define a threshold for a given clinical setting.

We agree with the reviewer. Different clinical settings may have distinct effects on TTV-DNA levels. Therefore, a universal threshold may not be appropriate across all patient populations or clinical contexts. We have included this statement following the respective passage in the revised manuscript.

Giving our data, defining this universal threshold for TTV-DNA levels indicative of adequate immunosuppression remains challenging, as these values must always be interpreted in the context of the individual baseline viral load.

R: With regard to the highly suppressed lung transplant patients it has been shown that the TTV levels that are reached upon immunsuppression post Tx are independent from the baseline differences (DOI: 10.1371/journal.pone.0122975). This could be considered in this regard.

We thank the reviewer for their suggestions and have added it to the corresponding paragraph. With the previous statement, we intended to emphasize that an absolute threshold above 10^6 cp/ml may not be universally applicable across all clinical situations, as also discussed in kidney transplant recipients. In particular, patients with already high baseline TTV levels and natural viral fluctuations may fall into this range even in the absence of iatrogenic immunosuppression.

We have revised the text to clarify this point more explicitly and to better delineate the scope of this assumption. In addition, we have excluded highly immunosuppressed lung transplant recipients from this specific interpretation to avoid overgeneralization, and we have cited the study mentioned by the reviewer at the corresponding passage.

Our findings demonstrate that TTV-DNA exhibits both short-term and long-term intra-individual dynamics even in the absence of immunosuppressive therapy. From the data available in this study it cannot be conclusively determined whether the observed changes reflect modulation of immune function by other factors affecting the immune system besides immunosuppressive medication, or whether they instead represent individual intrinsic variability in viral replication independent of immune alterations.

R: In overall, I agree with the authors. However, I would like to remark that in the SOT setting, particularly in LuTx patients, the TTV DNA load exceeds the TTV load found in non-immunsuppressive people even when, due to the fluctuations, the TTV load can reach 10e6 c/mL. Yet, in other clinical settings the observed „normal" fluctuations may not be distinguishable from medication-induced TTV load changes.

We thank the reviewer for this comment. Analogous to the previous comment, we have changed the corresponding passage in the revised manuscript regarding the substantially higher TTV-DNA levels reported in lung transplant recipients. We have also revised the statement to refer more specifically to published studies in kidney and heart transplant recipients, for whom considerably lower TTV thresholds have been proposed.

This individual "anellome" remains largely stable during adulthood (Timmerman et al., 2024, Kaczorowska et al., 2022), therefore, new infections with different viral variants are unlikely to underlie the observed interindividual fluctuations.

R: I am not sure if there are already enough data to state that new infections are unlikely.

We agree with the reviewer and have revised the statement accordingly to better reflect the limited data currently available and to avoid overstating the strength of the existing evidence.

13th Aug 2026

Dear Dr. Wettengel,

Thank you for the submission of your manuscript to EMBO Molecular Medicine. We have now received feedback from the two reviewers who agreed to evaluate your manuscript. As you will see from their reports pasted below both referees support publication of your manuscript. Therefore, I am pleased to inform you that we will be able to accept your manuscript pending the following final amendments:

1) Figures:
- Rename main figure files to Figure 1 etc.
- Please upload EV figure as separate high-resolution files and name them Figure EV1 etc. EV figure legends should be placed in the manuscript file after main figure legends under the heading "Expanded View Figure Legends".
2) Source Data: Please upload source data for the main figures as one zipped file per figure.
3) Title: Please consider revising the title to better emphasize the main findings, e.g. "A high-throughput Torque teno virus assay improves immune monitoring by defining intrinsic longitudinal viral load variability"
4) Abstract: I have gone through your text and revised it (see below). Please review it for accuracy and amend as you see fit:

Torque teno virus (TTV) DNA is an emerging biomarker of immune competence with considerable potential for guiding immunosuppressive therapy. However, widespread clinical implementation has been hampered by the lack of standardized, high-throughput diagnostic assays and limited understanding of the physiological variability of TTV viral loads over time. Here, we developed and analytically validated a fully automated high-throughput quantitative PCR assay for standardized TTV-DNA measurement in routine diagnostics and large-scale clinical studies. The assay demonstrated high analytical sensitivity and specificity, with a limit of detection of 32.3 copies/mL. Using this platform, we performed longitudinal profiling of plasma and serum samples collected over up to 51 months from non-immunosuppressed individuals. We observed substantial inter- and intra-individual fluctuations in TTV-DNA levels in a subset of healthy subjects, revealing intrinsic biological variability independent of immunosuppressive therapy. These findings establish a robust platform for standardized TTV monitoring while demonstrating that longitudinal viral load dynamics must be interpreted in the context of natural biological variation. Our study provides an important framework for the clinical implementation of TTV-guided immune monitoring and the development of evidence-based thresholds for individualized immunosuppressive management.

5) In the main manuscript file, please do the following:
- Please address all comments suggested by our data editors listed below:
o Figure legends:
1. Please note that the legends for figures 1, 3 is not provided in the sequential manner. This needs to be rectified.
2. Please note that the exact p values are not provided in the legends of figures 1A-F; 2B, C; 3B, C, D, F; 4B.
- Correct callout for Appendix Table 2 to Appendix Table S2.
- Rename "Materials and Methods" to "Methods".
- In Methods, provide the statement that informed consent was obtained from all human subjects and that experiments conformed to the principles set out in the WMA Declaration of Helsinki and in the Department of Health and Human Services Belmont Report.
- Please include structured Methods section that includes a Reagents and Tools Table (should be uploaded as a separate file) followed by a Methods and Protocols section. More information on how to adhere to this format as well as downloadable templates (.docx) for the Reagents and Tools Table can be found in our author guidelines:
https://www.embopress.org/page/journal/17574684/authorguide#structuredmethods
An example of a paper with Structured Methods can be found here:
https://www.embopress.org/doi/full/10.1038/s44320-024-00037-6#sec-4
- Indicate in legends exact n and exact p values, not a range, along with the statistical test used. To keep the figures "clear" some authors found providing an Appendix table Sx with all exact p-values preferable. You are welcome to do this if you want to.
- Rename "literature" to References and correct the reference citation in the reference list. Where there are more than 10 authors on a paper, 10 will be listed, followed by "et al.". https://link.springer.com/journal/44321/submission-guidelines#cms-Reference-guidelines
6) Appendix: Please upload it as PDF file with a table of contents incl. page numbers on the title page. Please correct the nomenclature to Appendix Figure S1 etc. and Appendix Table S1, etc.
7) Funding: Please provide information about all sources of funding in the "Acknowledgments" and in our submission system.
8) The Paper Explained: Please provide "The Paper Explained" and add it to the main manuscript text. Please check any of recently published EMBO Mol Med research articles for reference.
9) Synopsis: Every published paper now includes a 'Synopsis' to further enhance discoverability. Synopses are displayed on the journal webpage and are freely accessible to all readers. They include separate synopsis image and synopsis text.
- Synopsis image: Please provide a visual abstract as a high-resolution .jpeg file exactly 550 px-wide x 300 pixels high to illustrate your article.
- Synopsis text: Please provide a short standfirst (maximum of 300 characters, including space) as well as 2-5 one sentence

bullet points that summarize the paper as a .doc file. Please write the bullet points to summarize the key NEW findings. They should be designed to be complementary to the abstract - i.e. not repeat the same text. We encourage inclusion of key acronyms and quantitative information (maximum of 30 words / bullet point). Please use the passive voice.
- Please check your synopsis text and image before submission with your revised manuscript. Please be aware that in the proof stage minor corrections only are allowed (e.g., typos).
10) As part of the EMBO Publications transparent editorial process (see our Editorial at http://embomolmed.embopress.org/content/2/9/329), EMBO Molecular Medicine will publish online a Review Process File (RPF) to accompany accepted manuscripts. This file will be published in conjunction with your paper and will include the anonymous referee reports, your point-by-point response and all pertinent correspondence relating to the manuscript. Let us know if you want to remove or not any figures from it prior to publication. Please note that the Authors checklist will be published at the end of the RPF.
11) Please provide a point-by-point letter INCLUDING my comments as well as the reviewer's reports and your detailed responses (as Word file).

I look forward to reading a new revised version of your manuscript as soon as possible.

Yours sincerely,

Zeljko Durdevic

Zeljko Durdevic
Senior Editor
EMBO Molecular Medicine

*** Instructions to submit your revised manuscript ***

*** PLEASE NOTE *** As part of the EMBO Publications transparent editorial process initiative (see our Editorial at https://www.embopress.org/doi/pdf/10.1002/emmm.201000094), EMBO Molecular Medicine will publish online a Review Process File to accompany accepted manuscripts.

In the event of acceptance, this file will be published in conjunction with your paper and will include the anonymous referee reports, your point-by-point response and all pertinent correspondence relating to the manuscript. If you do NOT want this file to be published, please inform the editorial office at contact@embomolmed.org.

*****

When preparing your revised manuscript, please refer to our guidelines: https://link.springer.com/journal/44321/submission-guidelines#cms-Revised-submissions. We perform an initial quality control of all revised manuscripts before re-review; failure to include requested items will delay the evaluation of your revision.

We require:

1) A .docx formatted version of the manuscript text (including legends for main figures, EV figures and tables). Please make sure that the changes are highlighted to be clearly visible.

2) Individual production quality figure files as .eps, .tif, .jpg (one file per figure). For guidance, download the 'Figure Guide PDF': https://media.springernature.com/original/springer-cms/rest/v1/content/27825798/data/v1.

3) A .docx formatted letter INCLUDING the reviewers' reports and your detailed point-by-point responses to their comments. As part of the EMBO Press transparent editorial process, the point-by-point response is part of the Review Process File (RPF), which will be published alongside your paper.

4) A complete author checklist, which you can download from our author guidelines. Please insert information in the checklist that is also reflected in the manuscript. The completed author checklist will also be part of the RPF.

5) Please note that all corresponding authors are required to supply an ORCID ID for their name upon submission of a revised manuscript.

6) It is mandatory to include a 'Data Availability' section after the Materials and Methods. Before submitting your revision, primary datasets produced in this study need to be deposited in an appropriate public database, and the accession numbers and database listed under 'Data Availability'. Please remember to provide a reviewer password if the datasets are not yet public.

In case you have no data that requires deposition in a public database, please state so in this section. Note that the Data Availability Section is restricted to new primary data that are part of this study.

7) For data quantification: please specify the name of the statistical test used to generate error bars and P values, the number (n) of independent experiments (specify technical or biological replicates) underlying each data point and the test used to calculate p-values in each figure legend. The figure legends should contain a basic description of n, P and the test applied. Graphs must include a description of the bars and the error bars (s.d., s.e.m.).

8) At EMBO Press we ask authors to provide source data for the main manuscript figures. You will receive a separate email with instructions for providing source data with your revised manuscript, including how to upload and organize the files.

9) Our journal encourages inclusion of *data citations in the reference list* to directly cite datasets that were re-used and obtained from public databases. Data citations in the article text are distinct from normal bibliographical citations and should directly link to the database records from which the data can be accessed. In the main text, data citations are formatted as follows: "Data ref: Smith et al, 2001" or "Data ref: NCBI Sequence Read Archive PRJNA342805, 2017". In the Reference list, data citations must be labeled with "[DATASET]". A data reference must provide the database name, accession number/identifiers and a resolvable link to the landing page from which the data can be accessed at the end of the reference.

10) We replaced Supplementary Information with Expanded View (EV) Figures and Tables that are collapsible/expandable online. A maximum of 5 EV Figures can be typeset. EV Figures should be cited as 'Figure EV1, Figure EV2" etc... in the text and their respective legends should be included in the main text after the legends of regular figures.

- For the figures that you do NOT wish to display as Expanded View figures, they should be bundled together with their legends in a single PDF file called *Appendix*, which should start with a short Table of Content. Appendix figures should be referred to in the main text as: "Appendix Figure S1, Appendix Figure S2" etc.

- Additional Tables/Datasets should be labeled and referred to as Table EV1, Dataset EV1, etc. Legends have to be provided in a separate tab in case of .xls files. Alternatively, the legend can be supplied as a separate text file (README) and zipped together with the Table/Dataset file.

11) The paper explained: EMBO Molecular Medicine articles are accompanied by a summary of the articles to emphasize the major findings in the paper and their medical implications for the non-specialist reader. Please provide a draft summary of your article highlighting

- the medical issue you are addressing,

- the results obtained and

- their clinical impact.

This may be edited to ensure that readers understand the significance and context of the research. Please refer to any of our published articles for an example.

12) Author contributions: You will be asked to provide CRediT (Contributor Role Taxonomy) terms in the submission system. These replace a narrative author contribution section in the manuscript.

13) A Conflict of Interest statement should be provided in the main text.

14) Every published paper includes a 'Synopsis' to further enhance discoverability. Synopses are displayed on the journal webpage and are freely accessible to all readers. They include a short stand first (maximum of 300 characters, including space) as well as 2-5 one-sentences bullet points that summarizes the paper. Please write the bullet points to summarize the key NEW findings. They should be designed to be complementary to the abstract - i.e. not repeat the same text. We encourage inclusion of key acronyms and quantitative information (maximum of 30 words / bullet point). Please use the passive voice. Please attach these in a separate file or send them by email, we will incorporate them accordingly.

Please also suggest a striking image or visual abstract to illustrate your article as a PNG file 550 px wide x 300 px high.

15) Include a Reagents and Tools Table as part of the Methods section, which can be downloaded from our author guidelines.

EMBO Molecular Medicine has a "scooping protection" policy, whereby similar findings that are published by others during review or revision are not a criterion for rejection. Should you decide to submit a revised version, I do ask that you get in touch after three months if you have not completed it, to update us on the status.

*Additional important information regarding Figures

Each figure should be given in a separate file and should have the following resolution:
Graphs 800-1,200 DPI
Photos 400-800 DPI
Colour (only CMYK) 300-400 DPI"

Figures are not edited by the production team. All lettering should be the same size and style; figure panels should be indicated by capital letters (A, B, C etc). Gridlines are not allowed except for log plots. Figures should be numbered in the order of their appearance in the text with Arabic numerals. Each Figure must have a separate legend and a caption is needed for each panel.

*Additional important information regarding figures and illustrations can be found at
https://media.springernature.com/original/springer-cms/rest/v1/content/27825798/data/v1

***** Reviewer's comments *****

Referee #2 (Remarks for Author):

The authors have properly addressed all the comments and points raised. The manuscript is now suitable for publication in EMBO Molecular Medicine.

Referee #3 (Comments on Novelty/Model System for Author):

While the data given in this study are very interesting, the study remains largely descriptive. Nevertheless, this study provides important data for further studies on the virus-host interaction.

Referee #3 (Remarks for Author):

All comments and questions are fully addressed.

Dear Senior Editor Dr. Durdevic,

We would like to sincerely thank you for the acceptance of our manuscript for publication in EMBO Molecular Medicine. We greatly appreciate the thorough evaluation of our work throughout the review process and the constructive and valuable comments provided by the reviewers, which have helped us to further improve the manuscript. We believe that our findings provide relevant insights for the field and will be of interest to the scientific community.

We also sincerely thank you and the data editors for the careful assessment of our manuscript and for the detailed comments and recommendations. We have addressed all points raised and revised the manuscript and associated files accordingly.

Below, we provide a point-by-point response to each comment. All corresponding changes have been incorporated into the revised manuscript and supplementary material, as indicated in our responses.

1) Figures:

- Rename main figure files to Figure 1 etc.

- Please upload EV figure as separate high-resolution files and name them Figure EV1 etc. EV figure legends should be placed in the manuscript file after main figure legends under the heading "Expanded View Figure Legends".

All main figure files have been renamed accordingly as Figure 1, Figure 2, etc. We also have uploaded all Expanded View figures as separate high-resolution files and renamed them Figure EV1, Figure EV2, etc., as requested. The corresponding figure legends have been included in the main manuscript following the main figure legends under the heading “Expanded View Figure Legends”.

2) Source Data: Please upload source data for the main figures as one zipped file per figure.

Source data have been compiled and uploaded as separate zipped files for each main figure, as requested.

3) Title: Please consider revising the title to better emphasize the main findings, e.g. "A high-throughput Torque teno virus assay improves immune monitoring by defining intrinsic longitudinal viral load variability"

We thank the Editor for this helpful suggestion. We agree that the proposed formulation better emphasizes both the methodological advance and the main biological finding of our study. In accordance with the journal’s requirements, we have shortened the title while also taking into account the comments raised by the Editor and the reviewers. We have therefore revised the title accordingly to: A novel Torque teno virus qPCR assay reveals longitudinal viral load variability among non-immunosuppressed subjects

4) Abstract: I have gone through your text and revised it (see below). Please review it for accuracy and amend as you see fit:

Torque teno virus (TTV) DNA is an emerging biomarker of immune competence with considerable potential for guiding immunosuppressive therapy. However, widespread clinical implementation has been hampered by the lack of standardized, high-throughput diagnostic assays and limited understanding of the physiological variability of TTV viral loads over time. Here, we developed and analytically validated a fully automated high-throughput quantitative PCR assay for standardized TTV-DNA measurement in routine diagnostics and large-scale clinical studies. The assay demonstrated high analytical sensitivity and specificity, with a limit of detection of 32.3 copies/mL. Using this platform, we performed longitudinal profiling of

plasma and serum samples collected over up to 51 months from non-immunosuppressed individuals. We observed substantial inter- and intra-individual fluctuations in TTV-DNA levels in a subset of healthy subjects, revealing intrinsic biological variability independent of immunosuppressive therapy. These findings establish a robust platform for standardized TTV monitoring while demonstrating that longitudinal viral load dynamics must be interpreted in the context of natural biological variation. Our study provides an important framework for the clinical implementation of TTV-guided immune monitoring and the development of evidence-based thresholds for individualized immunosuppressive management.

We thank the Editor for revising the Abstract. We carefully reviewed the proposed text for accuracy and agree that it provides a clear and concise summary of the rationale, methodological advance, and principal findings of our study. We have incorporated the revised Abstract into the manuscript, with minor amendments where necessary to ensure consistency with the terminology and data presented in the manuscript. Sinsce the word limit is überschritten haben wir wie zuvor abgesprochen das revised abstract in unser manuscript eingefügt und im online formular das noch nicht revised abstract stehen gelassen.

We have changed the wording from “biomarker of immune competence” to “biomarker of the individual immune status” to ensure consistency with the terminology used throughout the manuscript. In addition, we have added “novel” to the description of our qPCR assay and slightly revised the final statement, “…and the need for the development of evidence-based thresholds for individualized immunosuppressive therapies.” to improve consistency and precision.

As the revised Abstract exceeds the word limit of the online submission system, we have, in consultation with the Editor, included the revised version in the manuscript.

5) In the main manuscript file, please do the following:

- Please address all comments suggested by our data editors listed below:

o Figure legends:

1. Please note that the legends for figures 1, 3 is not provided in the sequential manner. This needs to be rectified.

2. Please note that the exact p values are not provided in the legends of figures 1A-F; 2B, C; 3B, C, D, F; 4B.

Thank you for pointing this out. The legends for Figures EV 1 and EV 3 have been corrected and are now presented in the appropriate sequential order. We have also addressed the statistical analysis throughout the manuscript. To provide all statistical information in a comprehensive manner while maintaining the readability of the figure legends, we have added Appendix Table S4, which reports the exact p-values for all paired t-tests performed for the statistical comparisons presented in the figures. The corresponding figure legends now refer to this table where appropriate.

- Correct callout for Appendix Table 2 to Appendix Table S2.

This has been corrected. The table is now consistently referred to as “Appendix Table S2” throughout the manuscript.

- Rename "Materials and Methods" to "Methods".

The section heading “Materials and Methods” has been changed to “Methods” as requested.

- In Methods, provide the statement that informed consent was obtained from all human subjects and that experiments conformed to the principles set out in the WMA Declaration of Helsinki and in the Department of Health and Human Services Belmont Report.

We have revised the ethics statement in the Methods section to explicitly state that the study was conducted in accordance with the ethical principles set out in the WMA Declaration of Helsinki and the Department of Health and Human Services Belmont Report.

Regarding informed consent, our study was retrospective and exclusively used pseudonymized residual serum and EDTA plasma samples obtained during routine diagnostics. The use of these residual samples for the present study was reviewed and approved by the Ethics Committee of the TUM University Hospital (reference number 2024-410-S-CB). Therefore, individual informed consent was not required under the conditions approved by the Ethics Committee. We have clarified this explicitly in the revised Methods section rather than stating that informed consent was obtained, as such a statement would not accurately reflect the study design and ethical approval.

- Please include structured Methods section that includes a Reagents and Tools Table (should be uploaded as a separate file) followed by a Methods and Protocols section. More information on how to adhere to this format as well as downloadable templates (.docx) for the Reagents and Tools Table can be found in our author guidelines: https://www.embopress.org/page/journal/17574684/authorguide#structuredmethods

An example of a paper with Structured Methods can be found here:

https://www.embopress.org/doi/full/10.1038/s44320-024-00037-6#sec-4

We have restructured the Methods section according to the journal guidelines. A Reagents and Tools Table has been prepared and uploaded as a separate file, and the manuscript now contains the corresponding structured Methods section.

- Indicate in legends exact n and exact p values, not a range, along with the statistical test used. To keep the figures "clear" some authors found providing an Appendix table Sx with all exact p-values preferable. You are welcome to do this if you want to.

We thank the Editor for this suggestion. The figure legends have been revised to report the exact sample sizes (n) and the statistical tests used. To maintain clarity and readability of the figure legends, exact p-values for all paired t-tests are provided in a dedicated Appendix Table S4. Appropriate references to this table have been added to the corresponding figure legends.

- Rename "literature" to References and correct the reference citation in the reference list. Where there are more than 10 authors on a paper, 10 will be listed, followed by "et al.". https://link.springer.com/journal/44321/submission-guidelines#cms-Reference-guidelines

The heading “Literature” has been changed to “References”. The reference list has been revised according to the journal guidelines, including correction of the citation format. For publications with more than 10 authors, the first 10 authors are now listed followed by “et al.”.

6) Appendix: Please upload it as PDF file with a table of contents incl. page numbers on the title page. Please correct the nomenclature to Appendix Figure S1 etc. and Appendix Table S1, etc.

The Appendix has been revised accordingly and is provided as a separate PDF file. A table of contents including page numbers has been added to the title page. The nomenclature has been standardized throughout to “Appendix Figure S1”, “Appendix Figure S2”, etc., and “Appendix Table S1”, “Appendix Table S2”, etc.

7) Funding: Please provide information about all sources of funding in the "Acknowledgments" and in our submission system.

Since we did not receive any external funding and all work was financed exclusively through internal funds from the diagnostic and research departments of the TUM Institute of Virology, we are unfortunately unable to specify a direct source of funding for each individual author in the submission system. To clarify this for all parties, we have added a corresponding statement to the Acknowledgements section of the revised manuscript.

8) The Paper Explained: Please provide "The Paper Explained" and add it to the main manuscript text. Please check any of recently published EMBO Mol Med research articles for reference.

A “The Paper Explained” section has been prepared according to the format of recently published EMBO Molecular Medicine research articles and has been added to the main manuscript.

9) Synopsis: Every published paper now includes a 'Synopsis' to further enhance discoverability. Synopses are displayed on the journal webpage and are freely accessible to all readers. They include separate synopsis image and synopsis text.

- Synopsis image: Please provide a visual abstract as a high-resolution .jpeg file exactly 550 px-wide x 300 pixels high to illustrate your article.

- Synopsis text: Please provide a short standfirst (maximum of 300 characters, including space) as well as 2-5 one sentence bullet points that summarize the paper as a .doc file. Please write the bullet points to summarize the key NEW findings. They should be designed to be complementary to the abstract - i.e. not repeat the same text. We encourage inclusion of key acronyms and quantitative information (maximum of 30 words / bullet point). Please use the passive voice.

- Please check your synopsis text and image before submission with your revised manuscript. Please be aware that in the proof stage minor corrections only are allowed (e.g., typos).

We have prepared the requested Synopsis material. A visual abstract has been provided as a high-resolution JPEG file with the requested dimensions of 550 × 300 pixels. In addition, the Synopsis text has been prepared as a separate document and includes a standfirst of no more than 300 characters as well as bullet points summarizing the key new findings of the study. The bullet points have been formulated to complement rather than duplicate the Abstract and comply with the requested length and style requirements.

10) As part of the EMBO Publications transparent editorial process (see our Editorial at http://embomolmed.embopress.org/content/2/9/329), EMBO Molecular Medicine will publish online a Review Process File (RPF) to accompany accepted manuscripts. This file will be published in conjunction with your paper and will include the anonymous referee reports, your point-by-point response and all pertinent correspondence relating to the manuscript. Let us know if you want to remove or not any figures from it prior to publication. Please note that the Authors checklist will be published at the end of the RPF.

Thank you for this information. We acknowledge and agree to the publication of the Review Process File as described. We do not request the removal of any figures from the Review Process File prior to publication.

11) Please provide a point-by-point letter INCLUDING my comments as well as the reviewer's reports and your detailed responses (as Word file).

We have prepared a comprehensive point-by-point response letter that includes the Editor's comments, the reviewers' reports, and our corresponding detailed responses. The document has been provided as a Word file together with the revised submission.

We again thank the Editor, the data editors, and the reviewers for their careful assessment and constructive comments. We believe that the revisions made in response to these suggestions have improved the clarity, transparency, and presentation of our manuscript substantially.

Sincerely,

Jochen Wettengel and Lara Valder on behalf of the other authors

25th Sep 2026

Dear Dr. Wettengel,

We are pleased to inform you that your manuscript is accepted for publication and is now being sent to our publisher to be included in the next available issue of EMBO Molecular Medicine.

Your manuscript will be processed for publication by EMBO Press. It will be copy edited and you will receive page proofs prior to publication. Please note that you will be contacted by Springer Nature Author Services to complete licensing and payment information.

You may qualify for financial assistance for your publication charges - either via a Springer Nature fully open access agreement or an EMBO initiative. Check your eligibility: https://link.springer.com/journal/44321/how-to-publish-with-us

Should you be planning a Press Release on your article, please notify the production team via this form in order to coordinate publication and release dates: https://forms.cloud.microsoft/e/eH4A8JVP6g

If you have any questions, please do not hesitate to contact the Editorial Office. Thank you for your contribution to EMBO Molecular Medicine.

Yours sincerely,
Zeljko Durdevic

Zeljko Durdevic
Senior Editor
EMBO Molecular Medicine

>>> Please note that it is EMBO Molecular Medicine policy for the transcript of the editorial process (containing referee reports and your response letter) to be published as an online supplement to each paper. If you do NOT want this, you will need to inform the Editorial Office via email immediately. More information is available here: https://link.springer.com/partners/embo-press/editorial-policies#Peer%20review

EMBO Press Author Checklist

Corresponding Author Name: Jochen M. Wettengel	
Journal Submitted to: EMBO Molecular Medicine	
Manuscript Number: EMM-2026-23678	

USEFUL LINKS FOR COMPLETING THIS FORM
The EMBO Journal - Author Guidelines
EMBO Reports - Author Guidelines
Molecular Systems Biology - Author Guidelines
EMBO Molecular Medicine - Author Guidelines

Reporting Checklist for Life Science Articles (updated January
This checklist is adapted from Materials Design Analysis Reporting (MDAR) Checklist for Authors. MDAR establishes a minimum set of requirements in transparent reporting in the life sciences (see Statement of Task: 10.31222/osf.io/9sm4x). Please follow the journal's guidelines in preparing your
Please note that a copy of this checklist will be published alongside your article.

Abridged guidelines for figures
1. Data
The data shown in figures should satisfy the following conditions:
- ➞ the data were obtained and processed according to the field's best practice and are presented to reflect the results of the experiments in an accurate and unbiased manner.
- ➞ ideally, figure panels should include only measurements that are directly comparable to each other and obtained with the same assay.
- ➞ plots include clearly labeled error bars for independent experiments and sample sizes. Unless justified, error bars should not be shown for technical
- ➞ if n<5, the individual data points from each experiment should be plotted. Any statistical test employed should be justified.
- ➞ Source Data should be included to report the data underlying figures according to the guidelines set out in the authorship guidelines on Data

2. Captions
Each figure caption should contain the following information, for each panel where they are relevant:
- ➞ a specification of the experimental system investigated (eg cell line, species name).
- ➞ the assay(s) and method(s) used to carry out the reported observations and measurements.
- ➞ an explicit mention of the biological and chemical entity(ies) that are being measured.
- ➞ an explicit mention of the biological and chemical entity(ies) that are altered/varied/perturbed in a controlled manner.
- ➞ the exact sample size (n) for each experimental group/condition, given as a number, not a range;
- ➞ a description of the sample collection allowing the reader to understand whether the samples represent technical or biological replicates (including how many animals, litters, cultures, etc.).
- ➞ a statement of how many times the experiment shown was independently replicated in the laboratory.
- ➞ definitions of statistical methods and measures:
  - common tests, such as t-test (please specify whether paired vs. unpaired), simple χ2 tests, Wilcoxon and Mann-Whitney tests, can be unambiguously identified by name only, but more complex techniques should be described in the methods section;
  - are tests one-sided or two-sided?
  - are there adjustments for multiple comparisons?
  - exact statistical test results, e.g., P values = x but not P values < x;
  - definition of 'center values' as median or average;
  - definition of error bars as s.d. or s.e.m.

Please complete ALL of the questions below.
Select "Not Applicable" only when the requested information is not relevant for your study.

Materials

Newly Created Materials	Information included in the manuscript?	In which section is the information available? (Reagents and Tools Table, Materials and Methods, Figures, Data Availability Section)
New materials and reagents need to be available; do any restrictions apply?	Not Applicable	Material and Methods

Antibodies	Information included in the manuscript?	In which section is the information available? (Reagents and Tools Table, Materials and Methods, Figures, Data Availability Section)
For **antibodies** provide the following information:<br>- Commercial antibodies: RRID (if possible) or supplier name, catalogue number and or/clone number<br>- Non-commercial: RRID or citation	Not Applicable	

DNA and RNA sequences	Information included in the manuscript?	In which section is the information available? (Reagents and Tools Table, Materials and Methods, Figures, Data Availability Section)
**Short novel DNA or RNA including primers, probes**: provide the sequences.	Yes	Appendix Table 2

Cell materials	Information included in the manuscript?	In which section is the information available? (Reagents and Tools Table, Materials and Methods, Figures, Data Availability Section)
**Cell lines:** Provide species information, strain. Provide accession number in repository **OR** supplier name, catalog number, clone number, and/**OR** RRID.	Not Applicable	
**Primary cultures:** Provide species, strain, sex of origin, genetic modification status.	Not Applicable	
Report if the cell lines were recently **authenticated** (e.g., by STR profiling) and tested for mycoplasma contamination.	Not Applicable	

Experimental animals	Information included in the manuscript?	In which section is the information available? (Reagents and Tools Table, Materials and Methods, Figures, Data Availability Section)
**Laboratory animals or Model organisms:** Provide species, strain, sex, age, genetic modification status. Provide accession number in repository **OR** supplier name, catalog number, clone number, **OR** RRID.	Not Applicable	
**Animal observed in or captured from the field:** Provide species, sex, and age where possible.	Not Applicable	
Please detail **housing and husbandry conditions**.	Not Applicable	

Plants and microbes	Information included in the manuscript?	In which section is the information available? (Reagents and Tools Table, Materials and Methods, Figures, Data Availability Section)
**Plants:** provide species and strain, ecotype and cultivar where relevant, unique accession number if available, and source (including location for collected wild specimens).	Not Applicable	
**Microbes:** provide species and strain, unique accession number if available, and source.	Not Applicable	

Human research participants	Information included in the manuscript?	In which section is the information available? (Reagents and Tools Table, Materials and Methods, Figures, Data Availability Section)
If collected and within the bounds of privacy constraints report on age, sex and gender or ethnicity for all study participants.	Yes	Material and Methods

Core facilities	Information included in the manuscript?	In which section is the information available? (Reagents and Tools Table, Materials and Methods, Figures, Data Availability Section)
If your work benefited from core facilities, was their service mentioned in the acknowledgments section?	Not Applicable	

Design

Study protocol	Information included in the manuscript?	In which section is the information available? (Reagents and Tools Table, Materials and Methods, Figures, Data Availability Section)
If study protocol has been **pre-registered, provide DOI in the manuscript**. For clinical trials, provide the trial registration number **OR** cite DOI.	Not Applicable	
Report the **clinical trial registration number** (at ClinicalTrials.gov or equivalent), where applicable.	Not Applicable	

Laboratory protocol	Information included in the manuscript?	In which section is the information available? (Reagents and Tools Table, Materials and Methods, Figures, Data Availability Section)
Provide DOI OR other citation details if **external detailed step-by-step protocols** are available.	Not Applicable	

Experimental study design and statistics	Information included in the manuscript?	In which section is the information available? (Reagents and Tools Table, Materials and Methods, Figures, Data Availability Section)
Include a statement about **sample size** estimate even if no statistical methods were used.	Yes	Material and Methods and Figures
Were any steps taken to minimize the effects of subjective bias when allocating animals/samples to treatment (e.g. **randomization procedure**)? If yes, have they been described?	Yes	Material and Methods
Include a statement about **blinding** even if no blinding was done.	Yes	Material and Methods
Describe **inclusion/exclusion criteria** if samples or animals were excluded from the analysis. Were the criteria pre-established?<br><br>If sample or data points were omitted from analysis, report if this was due to attrition or intentional exclusion and provide justification.	Not Applicable	
For every figure, are **statistical tests** justified as appropriate? Do the data meet the assumptions of the tests (e.g., normal distribution)? Describe any methods used to assess it. Is there an estimate of variation within each group of data? Is the variance similar between the groups that are being statistically compared?	Yes	

Sample definition and in-laboratory replication	Information included in the manuscript?	In which section is the information available? (Reagents and Tools Table, Materials and Methods, Figures, Data Availability Section)
In the figure legends: state number of times the experiment was **replicated** in laboratory.	Yes	Figures
In the figure legends: define whether data describe **technical or biological replicates**.	Yes	Figures

Ethics

Ethics	Information included in the manuscript?	In which section is the information available? (Reagents and Tools Table, Materials and Methods, Figures, Data Availability Section)
Studies involving **human participants**: State details of **authority granting ethics approval** (IRB or equivalent committee(s), provide reference number for approval.	Yes	Material and Methods
Studies involving **human participants**: Include a statement confirming that **informed consent** was obtained from all subjects and that the experiments conformed to the principles set out in the WMA Declaration of Helsinki and the Department of Health and Human Services Belmont Report.	Yes	Material and Methods
Studies involving **human participants:** For publication of **patient photos**, include a statement confirming that consent to publish was obtained.	Not Applicable	
Studies involving experimental **animals**: State details of **authority granting ethics approval** (IRB or equivalent committee(s), provide reference number for approval. Include a statement of compliance with ethical regulations.	Not Applicable	
Studies involving **specimen and field samples:** State if relevant **permits** obtained, provide details of authority approving study; if none were required, explain why.	Not Applicable	

Dual Use Research of Concern (DURC)	Information included in the manuscript?	In which section is the information available? (Reagents and Tools Table, Materials and Methods, Figures, Data Availability Section)
Could your study fall under dual use research restrictions? Please check biosecurity documents and list of **select agents and toxins** (CDC): https://www.selectagents.gov/sat/list.htm	Not Applicable	
If you used a select agent, is the security level of the lab appropriate and reported in the manuscript?	Not Applicable	
If a study is subject to dual use research of concern regulations, is the name of the **authority granting approval and reference number** for the regulatory approval provided in the manuscript?	Not Applicable	

Reporting

The MDAR framework recommends adoption of discipline-specific guidelines, established and endorsed through community initiatives. Journals have their own policy about requiring specific guidelines and recommendations to complement MDAR.

Adherence to community standards	Information included in the manuscript?	In which section is the information available? (Reagents and Tools Table, Materials and Methods, Figures, Data Availability Section)
State if relevant guidelines or checklists (e.g., **ICMJE, MIBBI, ARRIVE, PRISMA**) have been followed or provided.	Not Applicable	
For **tumor marker prognostic studies,** we recommend that you follow the **REMARK** reporting guidelines (see link list at top right). See author guidelines, under 'Reporting Guidelines'. Please confirm you have followed these guidelines.	Not Applicable	
For **phase II and III randomized controlled trials**, please refer to the **CONSORT** flow diagram (see link list at top right) and submit the CONSORT checklist (see link list at top right) with your submission. See author guidelines, under 'Reporting Guidelines'. Please confirm you have submitted this list.	Not Applicable	

Data Availability

Data availability	Information included in the manuscript?	In which section is the information available? (Reagents and Tools Table, Materials and Methods, Figures, Data Availability Section)
Have **primary datasets** been deposited according to the journal's guidelines (see 'Data Deposition' section) and the respective accession numbers provided in the Data Availability Section?	Not Applicable	
Were **human clinical and genomic datasets** deposited in a public access-controlled repository in accordance to ethical obligations to the patients and to the applicable consent agreement?	Not Applicable	
Are **computational models** that are central and integral to a study available without restrictions in a machine-readable form? Were the relevant accession numbers or links provided?	Not Applicable	
If publicly available data were reused, provide the respective **data citations in the reference list**.	Not Applicable	
