# Supplementary material for: A novel Torque teno virus qPCR assay reveals longitudinal viral load variability among non-immunosuppressed subjects
Source: EMBO Mol Med. 2026 Oct 8;18(11):4618–42. doi: 10.1038/s44321-026-00540-3 (PMC13748663; doi:10.1038/s44321-026-00540-3)
Supplement: Supplementary file 3 [file 44321_2026_540_MOESM7_ESM.pdf]

Expanded View Figures

Figure EV1. Comparison of modified primers for TTV detection in a serial dilution.

(**A–C**) Ct values for the Maggi et al primer set and sample dilution. (**D–F**) RFI for each primer set and dilution. Three different sample dilutions were used to cover high and low viral titers: (**A**, **D**) Sample dilution 1:10; (**B**, **E**) Sample dilution 1:100; (**C**, **F**) Sample dilution 1:500. Measurement of a plasma sample serial dilution in triplicate. Data were shown as mean ± SD of triplicate measurements ($n = 3$). Statistical significance was assessed using a paired *t*-test. $P < 0.05$ is indicated by *, $P < 0.01$ by **, $P < 0.001$ by ***, and all comparisons not indicated were considered non-significant ($P > 0.05$). Exact *p* values are described in Appendix Table S4.

© The Author(s)

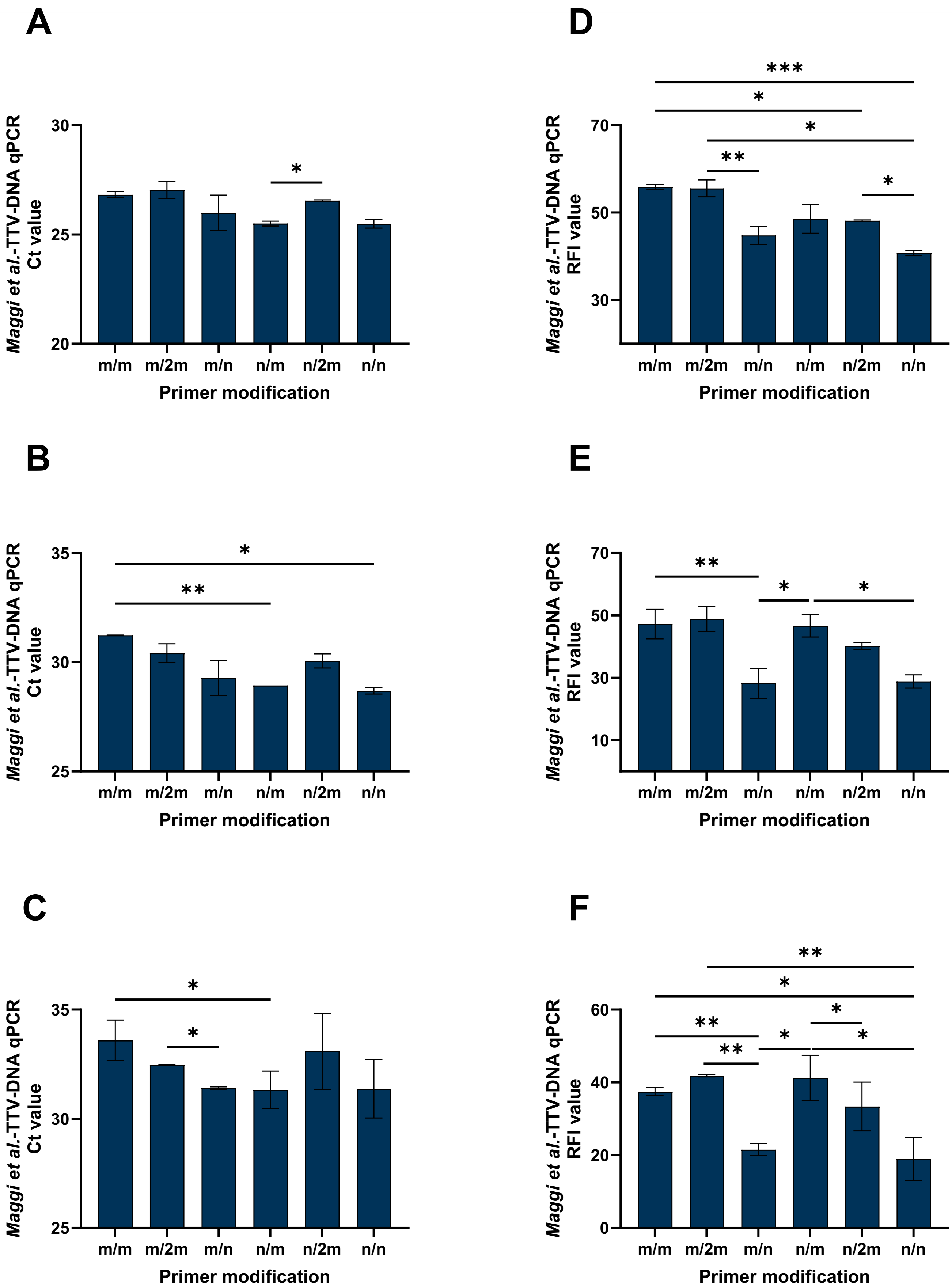
A
B
C
D
E
F
Maggi et al.-TTV-DNA qPCR
Ct value
RFI value
Primer modification
m/m
m/2m
m/n
n/m
n/2m
n/n
30
25
20
35
70
50
10
60
40
0
*
**
***

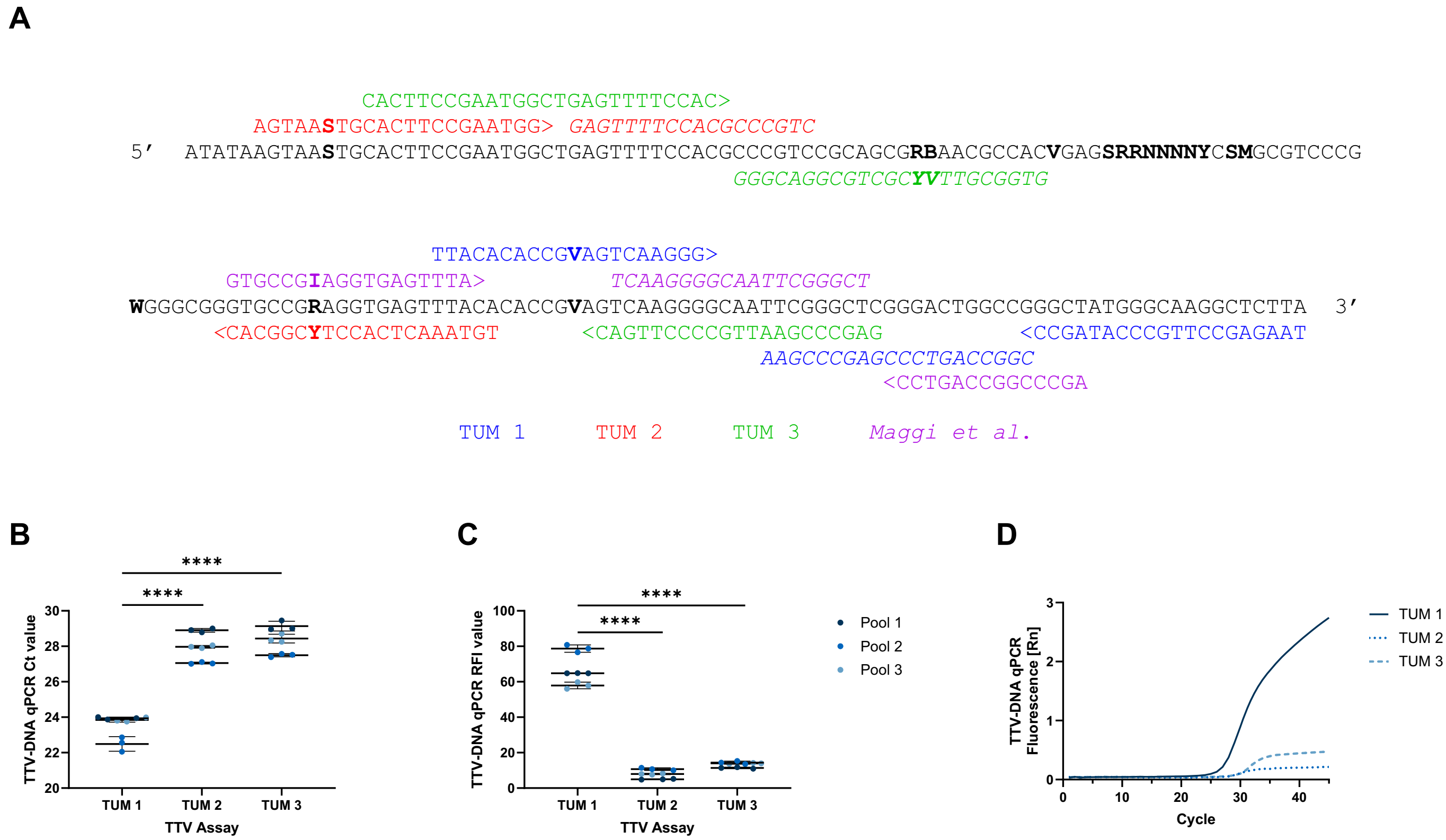

Figure EV2. Selection of TTV-DNA-specific primer and probes.

(**A**) Various primer pairs and probes are mapped across the TTV consensus sequence with 75% sequence similarity to facilitate detection and quantification in diagnostic assays. Degenerated bases are indicated in bold. Forward and reverse primers are marked with arrows in the direction of binding. Probes are indicated in italic. Blue: TUM 1, Red: TUM 2, Green: TUM 3; Purple: Maggi et al. (Maggi et al, 2001). (**B–D**) Comparison of different primer/probe sets for TTV-DNA detection. (**B**) Ct values and (**C**) RFI for each primer/probe set ($n = 9$), showing variation in sensitivity and signal strength. Sets with lower Ct values and higher RFI demonstrate superior sensitivity for TTV-DNA detection. (**D**) qPCR amplification curves demonstrate the fluorescence increase per cycle.(**B**, **C**) Data were shown as single measurements with mean ± SD of triplicate measurements. Statistical significance was assessed using a paired *t*-test. $P < 0.0001$ is indicated by ****, and all comparisons not indicated were considered non-significant ($P > 0.05$). Exact *p* values are described in Appendix Table S4.

 © The Author(s)

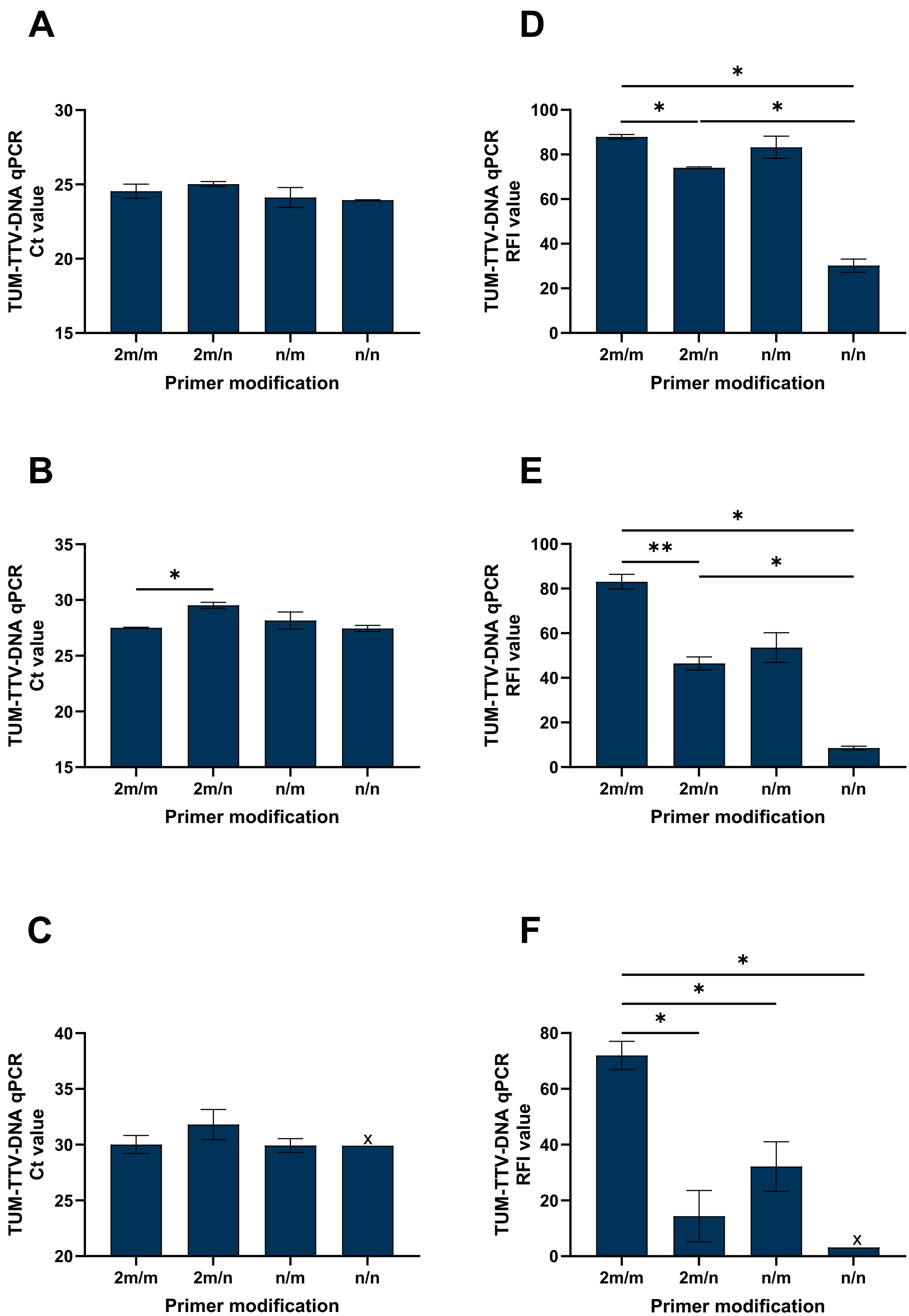
A
TUM-TTV-DNA qPCR Ct value
30
25
20
15
2m/m
2m/n
n/m
n/n
Primer modification
D
TUM-TTV-DNA qPCR RFI value
100
80
60
40
20
0
*
*
*
2m/m
2m/n
n/m
n/n
Primer modification
B
TUM-TTV-DNA qPCR Ct value
35
30
25
20
15
*
2m/m
2m/n
n/m
n/n
Primer modification
E
TUM-TTV-DNA qPCR RFI value
100
80
60
40
20
0
*
**
*
2m/m
2m/n
n/m
n/n
Primer modification
C
TUM-TTV-DNA qPCR Ct value
40
35
30
25
20
x
2m/m
2m/n
n/m
n/n
Primer modification
F
TUM-TTV-DNA qPCR RFI value
80
60
40
20
0
*
*
*
x
2m/m
2m/n
n/m
n/n
Primer modification

Figure EV3. Comparison of modified primers for TTV detection in a serial dilution.

(**A–C**) Ct values for the TUM 1 primer set and sample dilution. (**D–F**) RFI for each primer set and dilution. Three different sample dilutions were used to cover high and low viral titers: (**A**, **D**) Sample dilution 1:10; (**B**, **E**) Sample dilution 1:100; (**C**, **F**) Sample dilution 1:500. Measurement of a plasma sample serial dilution in triplicate. Data were shown as mean ± SD of triplicate measurements ($n = 3$). Statistical significance was assessed using a paired *t*-test. $P < 0.05$ is indicated by *, $P < 0.01$ by **, and all comparisons not indicated were considered non-significant (ns, $P > 0.05$). x indicates that only one replicate could be detected. Exact *p* values are described in Appendix Table S4.

 © The Author(s)

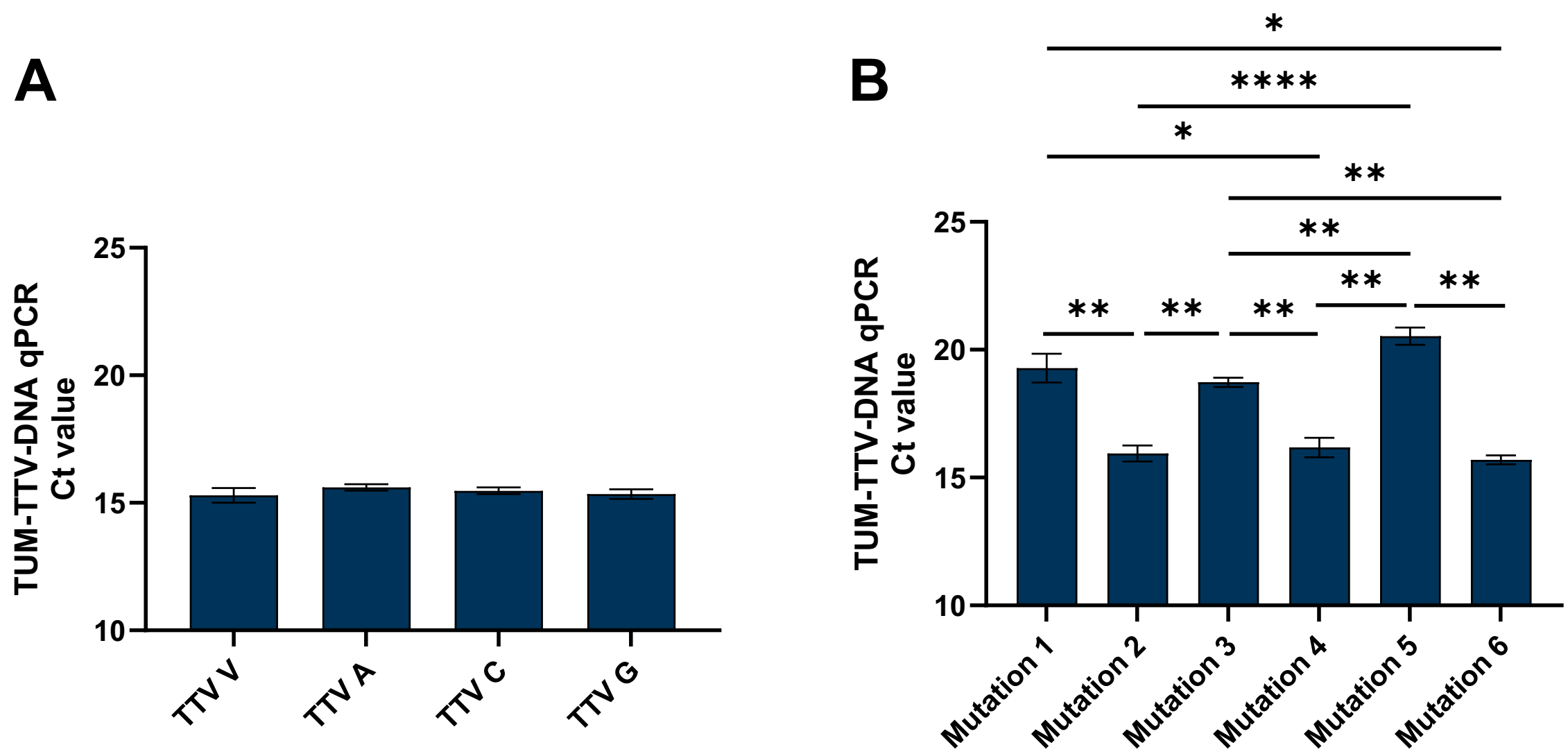

Figure EV4. Comparison of TTV detection of different TTV constructs and mutations.

(**A**) As a reference, TTV V represents the standard target sequence. TTV A, C, and G each represent the individual sequences contained within TTV V. (**B**) The following six mutations represent the most common mutations. Plasmids were measured in triplicate and the mean and standard deviation was calculated. Data were shown as mean ± SD of triplicate measurements ($n = 3$). Statistical significance was assessed using a paired *t*-test. $P < 0.05$ is indicated by *, $P < 0.01$ by **, $P < 0.0001$ by ****, and all comparisons not indicated were considered non-significant ($P > 0.05$). Exact *p* values are described in Appendix Table S4.

© The Author(s)

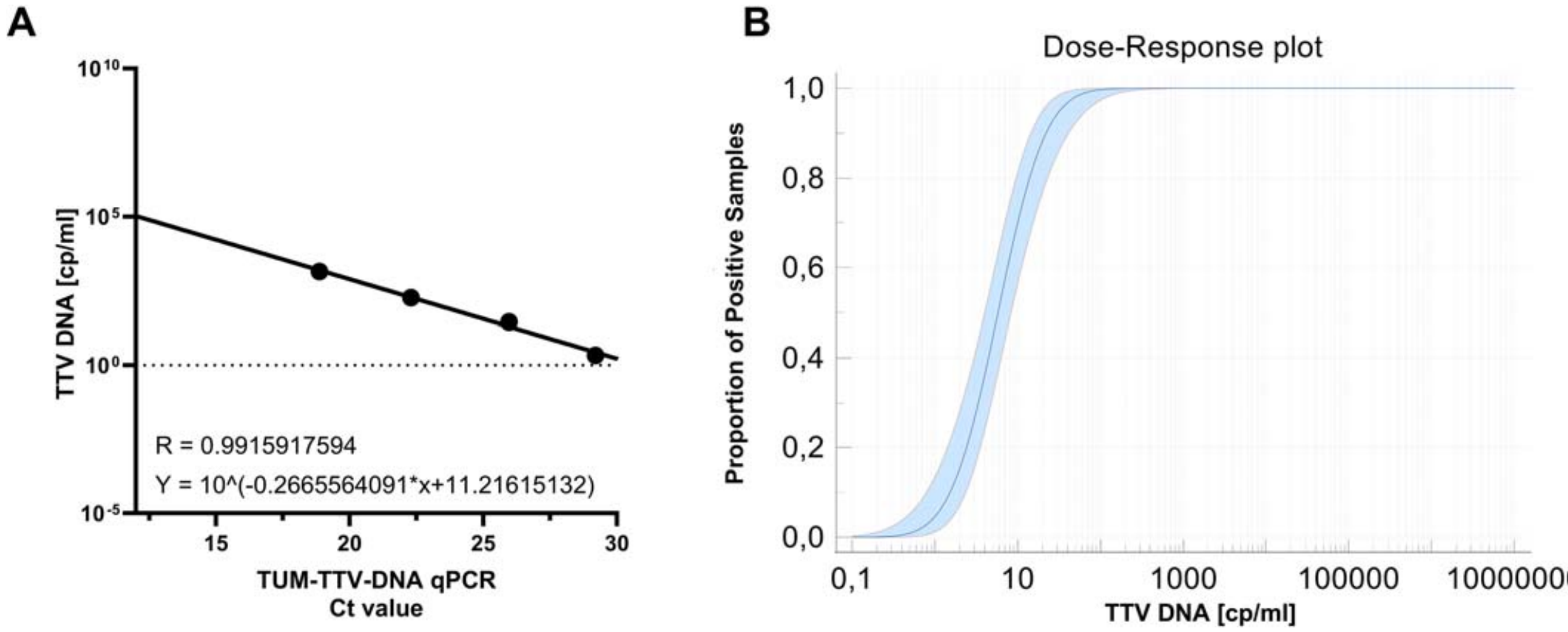

Figure EV5. Analytical performance of the TUM 1 TTV PCR Assay on the cobas 5800.

(**A**) Standard curve for assay quantification. Reference samples quantified by digital PCR (dPCR) and expressed as viral load (copies/mL) were plotted against the corresponding cycle threshold (Ct) values obtained on the cobas 5800. Nonlinear regression analysis was performed to derive the conversion equation used to calculate viral load (cp/ml) from measured Ct values. (**B**) Determination of the limit of detection (LOD). A serial dilution of a positive clinical sample was prepared, and each dilution level was measured 15 times ($n = 15$). The LOD was defined as the lowest concentration at which ≥95% of replicates were detected, based on probit analysis.

 © The Author(s)

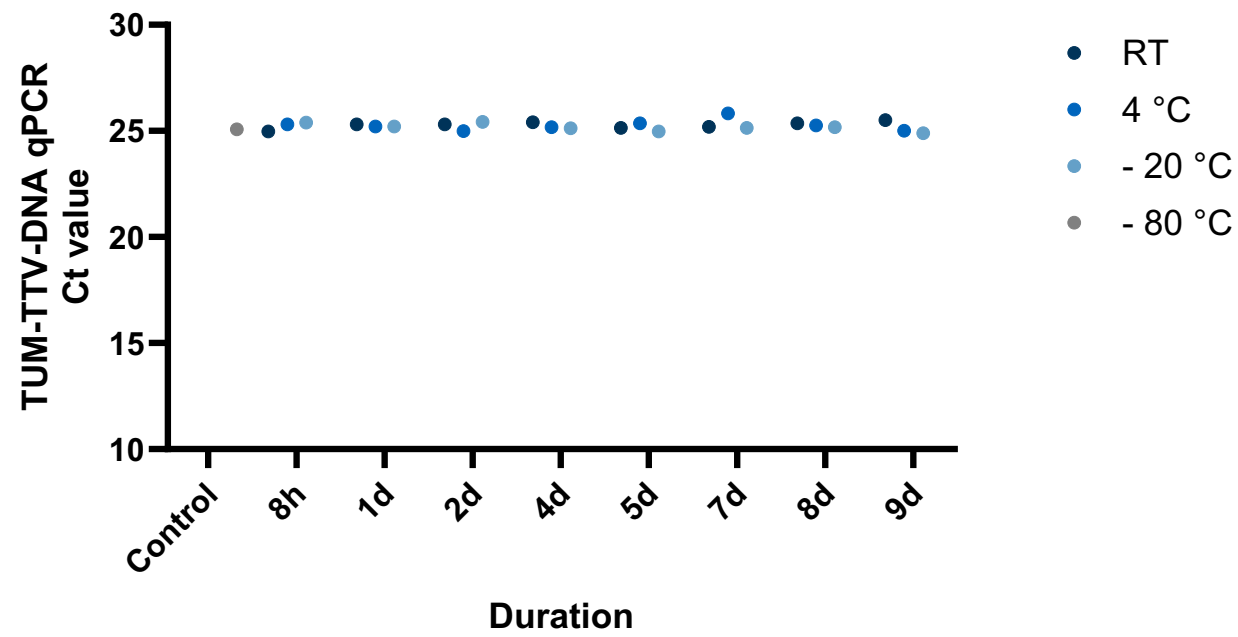

Figure EV6. TTV stability at different temperatures.

The samples were stored for a specific number of days at various temperatures (RT, 4 °C, −20 °C) and subsequently kept at −80 °C. A control sample, which was exclusively stored at −80 °C, was also used for comparison. Data were presented as the mean of triplicate measurements ($n = 3$). Two-way ANOVA revealed no significant effect of temperature (F (3, 21) = 1.897, $P = 0.1611$), and no significant effect of time (F (7, 21) = 0.3323, $P = 0.9302$).
